# *Azolla* as a Safe Food: Suppression of Cyanotoxin-Related Genes and Cyanotoxin Production in Its Symbiont, *Nostoc azollae*

**DOI:** 10.3390/plants13192707

**Published:** 2024-09-27

**Authors:** Jonathan P. Bujak, Ana L. Pereira, Joana Azevedo, Alexandra A. Bujak, Victor Leshyk, Minh Pham Gia, Timo Stadtlander, Vitor Vasconcelos, Daniel J. Winstead

**Affiliations:** 1 The Azolla Foundation, Burbage Lodge, 200 Queens Promenade, Blackpool FY2 9JS, UK; alexandra-bujak@hotmail.com; 2 Centro Interdisciplinar de Investigação Marinha e Ambiental, CIIMAR, University of Porto, Avenida General Norton de Matos, s/n, 4450-208 Matosinhos, Portugal; anapereira271268@yahoo.com (A.L.P.); joana.azevedo@ciimar.up.pt (J.A.); vmvascon@fc.up.pt (V.V.); 3 Azolla Biodesign, 65 Vista Lane, Sedona, AZ 86351, USA; victorleshyk@azollabiodesign.com; 4 Independent Researcher, E4 Building, app.12A07, Hanoi 100000, Vietnam; phamgiaminhvn@gmail.com; 5 The Research Institute of Organic Agriculture (FiBL), Ackerstrasse 113, CH-5070 Frick, Switzerland; timo.stadtlander@fibl.org; 6 Department of Biology, FCUP-Faculty of Sciences, University of Porto, 4169-007 Porto, Portugal; 7 Department of Ecosystem Science and Management, College of Agricultural Sciences, The Pennsylvania State University, University Park, PA 16802, USA; djw5623@psu.edu

**Keywords:** *Azolla*, *Nostoc*, *Anabaena*, symbiosis, BMAA, microcystins, nodularin, anatoxin-a, cylindrospermopsin, saxitoxin

## Abstract

The floating freshwater fern *Azolla* is the only plant that retains an endocyanobiont, *Nostoc azollae* (aka *Anabaena azollae*), during its sexual and asexual reproduction. The increased interest in *Azolla* as a potential source of food and its unique evolutionary history have raised questions about its cyanotoxin content and genome. Cyanotoxins are potent toxins synthesized by cyanobacteria which have an anti-herbivore effect but have also been linked to neurodegenerative disorders including Alzheimer’s and Parkinson’s diseases, liver and kidney failure, muscle paralysis, and other severe health issues. In this study, we investigated 48 accessions of *Azolla–Nostoc* symbiosis for the presence of genes coding microcystin, nodularin, cylindrospermopsin and saxitoxin, and BLAST analysis for anatoxin-a. We also investigated the presence of the neurotoxin β-N-methylamino-L-alanine (BMAA) in *Azolla* and *N. azollae* through LC-MS/MS. The PCR amplification of saxitoxin, cylindrospermospin, microcystin, and nodularin genes showed that *Azolla* and its cyanobiont *N. azollae* do not have the genes to synthesize these cyanotoxins. Additionally, the matching of the anatoxin-a gene to the sequenced *N. azollae* genome does not indicate the presence of the anatoxin-a gene. The LC-MS/MS analysis showed that BMAA and its isomers AEG and DAB are absent from *Azolla* and *Nostoc azollae*. *Azolla* therefore has the potential to safely feed millions of people due to its rapid growth while free-floating on shallow fresh water without the need for nitrogen fertilizers.

## 1. Introduction

*Azolla* Lam. is the only plant with a permanent nitrogen-fixing cyanobacterial symbiont (cyanobiont) that has chains of cells (filaments) comprising photosynthetic vegetative cells and thicker-walled heterocysts that contain the nitrogen-fixing enzyme nitrogenase [1]. The cyanobiont has been assigned to both *Anabaena azollae* and *Nostoc azollae* because its morphology resembles free-living species of both genera, including their change into motile hormogonia and akinetes (resting cells) that ensure survival during stressed conditions [2].

Genetic and paleontological data indicate that the *Azolla–N. azollae* symbiosis originated 80 million years ago in North America following whole-genome duplication (WGD) that increased the genome of *Azolla*’s immediate ancestor [3]. *Nostoc azollae*’s subsequent coevolution with *Azolla* caused extensive changes in the cyanobiont’s genome compared to free-living species of *Anabaena* and *Nostoc* [3,4,5,6,7,8]. Some changes involved the upregulation of genes that enhanced *N. azollae*’s sequestration of atmospheric nitrogen and provision of nitrogen-based compounds to *Azolla*, increasing the plant’s speed of growth while free-floating on fresh water. The downregulation, loss, or conversion to pseudogenes of other genes changed *N. azollae*’s ancestors from independent free-living organisms into obligate endosymbionts, reflecting *N. azollae*’s permanent location inside the leaves and female megasporocarps of *Azolla*. These included genes that previously expressed proteins involved in the synthesis of carotenoid and chlorophyll pigments, so that *A. azollae* is reliant on *Azolla*’s cellular pigments for protection against photooxidative damage [6].

Colonies of *N. azollae* live in specialized cavities in *Azolla*’s dorsal floating leaves, providing nitrogen-based nutrients to the plant that enable it to double its biomass in less than two days while free-floating on fresh water [9,10]. As a result, *Azolla* has been used for hundreds of years in India and the Far East as a nitrogen biofertilizer for paddy rice, reducing mosquito breeding populations by 95% [11,12,13] and emissions of the potent greenhouse gas methane from paddies by 25–50% [14,15,16]. *Azolla* also provides livestock feed, biofuel and biofertilizer for other plants, alleviating shortages of the ‘three Fs’ that increasingly threaten food supplies globally: feed, fuel, and fertilizer. It absorbs and removes phosphates and nitrates from water contaminated by chemical fertilizers, industrial pollutants, and animal and human waste that trigger toxic cyanobacterial (aka blue-green algal) blooms in rivers and lakes. The symbionts’ combined CO_2_ sequestration also increases *Azolla*’s carbon capture so that it can sequester large amounts of atmospheric CO_2_, with the plants being compressed and stored to reduce anthropogenic climate change through carbon capture and storage (CCS). *Azolla* can, therefore, mitigate many of the threats arising from the ‘perfect storm’, as our population increases by more than a million every three days. Its remarkable properties are increasingly recognised, and one study has designated it a unique superorganism [1].

*Azolla* has the potential to help feed millions of people because of its rapid growth, ease of outdoor cultivation in tropical and temperate regions, and global production using the indoor *Azolla* biosystem described by Bujak & Bujak (2020) in ‘The Azolla Story’ [2]. The use of *Azolla* for human consumption was thought to be limited by its high total polyphenolic content (TPC), but Winstead et al. (2024) [17] showed that the TPC of raw *Azolla caroliniana*, which is native in the eastern United States, has only 4.26 g gallic acid equivalent (GAE) kg^−1^ DW and that simple cooking methods can decrease TPC in all *Azolla* species. They also demonstrated that its protein content is 19% DW and its apparent protein digestibility is 78.45%, with a yield of 173 g FW m^−2^ day^−1^ and 5.53 g DW m^−2^ day^−1^, confirming *Azolla*’s potential for cultivation and domestication as a nutritious food. This raises the question as to whether *Azolla* is safe to eat because of the presence of harmful cyanotoxins in many cyanobacteria.

Cyanotoxins are produced by cyanobacteria of the genera *Anabaena* and *Nostoc* among others and include some of the most powerful natural poisons that target the nervous system (neurotoxins such as BMAA, saxitoxin, and anatoxin-a), the liver (hepatotoxins such as microcystins and nodularins), protein synthesis and DNA modification (cylindrospermopsin), and the skin (dermatoxins, such as nodularins). Cyanotoxins are alkaloids (anatoxin-a, saxitoxin, cylindrospermopsin) or peptides (pentapeptide nodularin or the heptapeptide microcystin) [18,19,20,21]. Upon their release in water, they are ingested by zooplankton and animals or absorbed by phytoplankton and plants, which can have acute or chronic effects when eaten by humans. This is a global health issue, owing to bioconcentration and bioaccumulation in the food chain and poisoning through ingestion of contaminated food, so cyanotoxins are now widely analyzed and studied to determine their effects on plants and animals [22]. For example, the World Health Organization (WHO) recommends a value of 1 μg/L for microcystin-LR in drinking water [23].

BMAA (β-N-methylamino-L-alanine) is a non-proteinogenic amino acid produced by free-living cyanobacteria in marine, freshwater, and terrestrial environments [24,25]. It has been detected in plants with endosymbiotic cyanobacteria including lichens, hornworts, the leaf petioles of the tropical flowering plant *Gunnera*, and the cycad *Cycas circinalis* [24,25,26] and linked to the amyotrophic lateral sclerosis/Parkinson–dementia complex (ALS/PDC) detected among Chamorro people living on the Pacific island of Guam [24,25]. BMAA, like other cyanotoxins, can be biomagnified in seafood eaten by people, including fish [27,28], shrimps [29], mussels, oysters, and crabs [30]. BMAA can also be synthetized by eukaryotes such as diatoms [31] and dinoflagellates [32], which are food sources for crustaceans, fish and shellfish [33]. However, the genes related to the BMAA biosynthetic pathway are not known.

These observations raise the question as to whether eating *Azolla* may be harmful to humans due to the possible production of BMAA and other cyanotoxins by *N. azollae*. Unlike free-living *Anabaena* and *Nostoc*, the loss or conversion to pseudogenes of genes involved in cyanotoxin and/or BMAA production may have occurred in *N. azollae* because they were no longer needed by the permanently enclosed cyanobiont. The following analyses were therefore undertaken on all seven extant *Azolla* species and their cyanobionts to determine if they can be safely eaten by people.

- The presence of genes coding for microcystin, nodularin, cylindrospermopsin, and saxitoxin.
- The presence of the anatoxin-a/homoanatoxin-a gene cluster by bioinformatic tools.
- The presence of BMAA.

The seven examined species of *Azolla* are *A. caroliniana*, *A. filiculoides*, *A. mexicana*, *A. microphylla*, *A. nilotica, A. rubra* and *A. pinnata,* including its two subspecies *A. pinnata* subsp. *pinnata* and *A. pinnata* subsp. *imbricata*. Table 1 lists the 48 accessions that provided the *Azolla* species and subspecies used in this study.

## 2. Results

### 2.1. The Cyanotoxins Microcystin, Nodularin, Saxitoxin, Cylindrospermopsin and Anatoxin-a

The PCR amplification of 12 genes that encode for 4 cyanotoxins (cylindrospermopsin, nodularin, saxitoxin and microcystin) was determined for all 7 *Azolla* species and 2 *A. pinnata* subspecies from 48 *Azolla* accessions listed in Table 1. The global distribution of the accessions’ countries of origin is shown in Supplementary Material Figure S25. The results indicate that cyanotoxin genes amplified on *Azolla* accessions were negative when matched with positive and negative controls for 12 genes: *cyl*, *mcy* A, *mcy* B, *mcy* B domain A, *mcy* C, *mcy* C domain A, *mcy* D domain ACP, *mcy* D domain KS, *mcyE/ndaF*, *mcy* E domain GSA-AMT, *mcy* G domain CM, and *sxt*.

The results also showed that same 12 genes in *N. azollae* isolated from the 47 *Azolla* accessions were not amplified compared with positive and negative controls. This indicates that both *Azolla* and *N. azollae* do not have genes that biosynthesize those cyanotoxins. Photographs of all gels from the PCR amplifications are shown in Supplementary Material Figures S1–S24.

The BLAST search for the anatoxin-a and homoanatoxin-a gene cluster showed a query cover of only 3% and percentage identity of 73.04%. Most of these alignments were partial segments comprising less than 250 bp of the *anaH* transposase gene with identities around 70%. The aligned genes in *N. azollae* are only associated with pseudogenes and not with protein coding genes. All other alignments were partial, and none included any of the whole genes associated with the anatoxin-a biosynthesis gene cluster.

### 2.2. Detection of BMAA (β-N-Methylamino-L-Alanine)

The detection of the non-proteinogenic amino acid BMAA by LC-MS/MS with method 1 on all seven *Azolla* species (*A. caroliniana*, *A. filiculoides*, *A. microphylla*, *A. mexicana*, *A. nilotica*, *A. rubra* and the two subspecies of *A. pinnata)* showed that BMAA was absent from both *Azolla* and its cyanobiont *N. azollae,* since the retention time for BMAA and the isomer 2,4-DAB (2,4-diaminobutyric acid) could not be found when compared with their standards.

**Figure 1:**
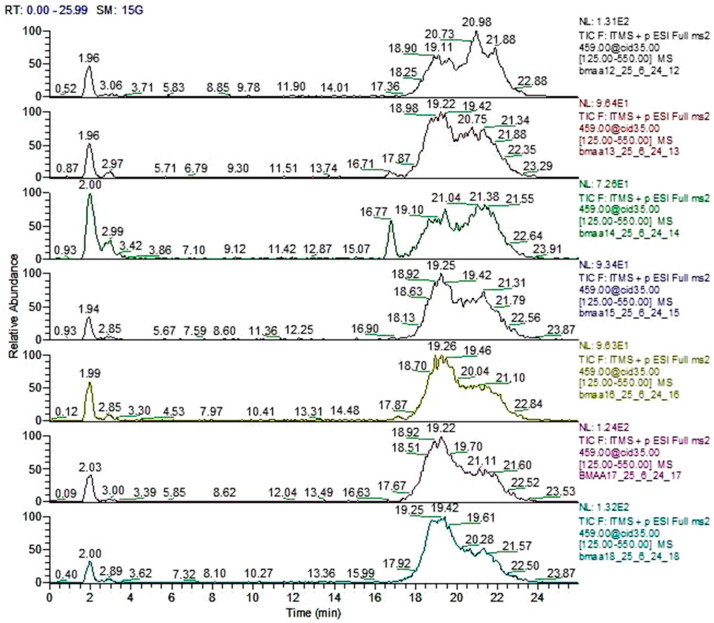
Total ion chromatogram of six *Azolla* species for the detection of BMAA with method 2. From top to bottom: *A. caroliniana* (CA 3001), *A. filiculoides* (FI 1507), *A. pinnata* subsp. *pinnata* (PP 7001), *A. nilotica* (NI 5001), *A. mexicana* (ME 2026), *A. microphylla* (MI 4021), *A. pinnata* subsp. *imbricata* (PI 1).

**Figure 2:**
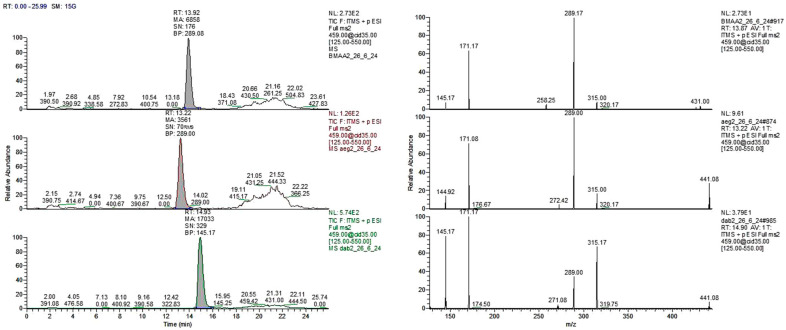
Total ion chromatogram (left) and CID spectra (right) of derivatized standards at 1 ppm. BMAA (top, RT = 13.92 min), AEG (middle, RT = 13.22 min), and 2,4-DAB (bottom, RT = 14.93 min).

Re-analysis of six of the *Azolla* species (excluding *A. rubra*, which was not re-analyzed) using method 2, in which the samples were derivatized, corroborated the results obtained with method 1. This indicates that the *Azolla* species shown in Figure 1 did not show the retention times for BMAA (RT = 13.92 min) and the two isomers 2,4-DAB (RT = 14.93 min) and AEG (N-(2-aminoethyl)-glycine) (RT = 13.22 min) when compared with their standards (Figure 2).

## 3. Discussion

Most genera of free-living cyanobacteria synthesize cyanotoxins, but their expression depends on environmental factors such as nutrients, light and temperature [22]. Some cyanobacteria also have temporary symbioses with plants, so that the host plant has the potential to assimilate and bioaccumulate the cyanotoxins, discussed above. There are few published studies documenting genetic and chromatographic detection of cyanotoxins in cyanobionts. Cyanobacteria from lichens have been analysed and contain genes that encode nodularin and microcystin and can translate the peptides nodularin and microcystins [34,35,36]. The synthesis of those two cyanotoxins may be linked to the temperature and humidity in which the lichens grow and may be important for the maintenance of lichens in diverse ecological habitats [37].

Unlike lichens, the fern *Azolla* has a permanent symbiosis with the cyanobacteria *N. azollae,* giving this symbiosis a unique evolution pattern and, ultimately, the loss of genes by the cyanobiont [4,5,6,7]. Our genetic analyses show, for or the first time, that all seven *Azolla* species and their cyanobiont, *N. azollae*, do not possess genes associated with the synthesis of microcystin, nodularin, saxitoxin, cylindrospermopsin, anatoxin-a, and homoanatoxin-a. The biosynthetic pathways of microcystins [38] and anatoxin-a/homoanatoxin-a [39] are a multi-step process that requires several genes to synthesize both cyanotoxins. All genes associated with microcystin synthesis were, therefore, amplified by specific primers with *Azolla* and *N. azollae* DNA, and the complete anatoxin-a gene cluster was BLAST-searched against the *N. azollae* genome. There were no matches, thereby supporting the hypothesis that *N. azollae* lost the ability to synthesise microcystin, nodularin, saxitoxin, cylindrospermopsin, anatoxin-a, and homoanatoxin-a due to downregulation of cyanotoxin biosynthesis genes or loss of the genes during the co-evolution of *Azolla* and *N. azollae*.

BMAA was isolated in 1967 from seeds of *Cycas circinalis* (cycad) [24,25,26] and identified as the primary cause of amyotrophic lateral sclerosis/Parkinson–dementia complex (ALS/PDC) in the Chamorro people on the Pacific island of Guam [40,41], with the high levels of the neurotoxin resulting from biomagnification through the food chain [27,28,33,42,43]. BMAA is a cyanotoxin that can cross the blood–brain barrier where it forms a reservoir [44]) and can be inserted into proteins instead of the amino acid L-serine, causing protein misfolding and aggregation [45,46,47]. BMAA can induce changes in the expression of genes in brain cells, thus resulting in a wide range of other neurodegenerative disorders [19]. Alzheimer’s, Parkinson’s and other neurological diseases including amyotrophic lateral sclerosis (ALS), progressive supranuclear palsy (PSP), and dementia with Lewy bodies (DLB) [48] may therefore be partially caused or facilitated by BMAA. However, the gene/genes for the codification of BMAA are not known in any cyanobacteria and plant, so that their presence can only be detected by analytical methods, including those used in this study.

BMAA was detected in examples of plant–cyanobacteria symbiosis such as hornworts, liverwort, lichens, cycads, and *Gunnera* [24], and also in *A. filiculoides* with 2 μg/g [42]. Some analytical methods of detecting this non-proteinogenic molecule can result in erroneous interpretations due to structural isomers DAB (2,4-diaminobutyric) and AEG (N-(2-aminoethyl)-glycine, which can co-elute and be mis-identified as BMAA [49,50]. For the present study, two methods were therefore used to detect BMAA, DAB, and AEG; we did not detect BMAA, AEG, or DAB in any of the analyzed *Azolla* and *N. azollae*. These data indicate that the previous reported detection of 2 μg/g BMAA in *Azolla* [42] is incorrect. It is possible that environmental factors affect the syntheses of cyanotoxins, including BMAA. Future studies could therefore analyze plant–cyanobacteria symbiosis collected from a variety of locations to determine if environments conditions influence the synthesis of cyanotoxins, including BMAA.

Harmful algal blooms (HABs) of other cyanobacteria species also release cyanotoxins upon cell necrosis. The uptake and bioaccumulation of cyanotoxins from irrigated water for crop and non-crop plants therefore also need to be evaluated to determine if *Azolla* species may bioaccumulate cyanotoxins. Previous studies have shown that *A. filiculoides* does now uptake or bioaccumulate microcystin [51] or cylindrospermospin [52], confirming that *Azolla* can be safely eaten.

## 4. Materials and Methods

### 4.1. Azolla Accessions and Culturing

Seven *Azolla* species, including two *A. pinnata* subspecies from the germplasm collection at IRRI (International Rice Research Institute, Laguna, Philippines) and two *A. filiculoides* accessions from Portugal (FI-BGLU and FI-BGM), were used to detect the cyanotoxin genes of microcystin, nodularin, cylindrospermospin, and saxitoxin (by PCR) and BMAA (by LC-MS/MS) (Table 1). The 48 *Azolla* accessions have a global distribution throughout 33 countries (Supplementary Material, Figure S25). The *Azolla* species were cultured in Hoagland medium (H-40), pH 6.1–6.2, with a controlled temperature (23–24 °C), photoperiod (16 h light/8 h dark), and light intensity (6.0 Wm^−2^) [53]. After 28 days, the biomass of fully developed *Azolla* was collected, washed in distilled water, frozen at −80 °C, lyophilized, and weighed.

### 4.2. Isolation of Nostoc azollae from Azolla Accessions

*Nostoc azollae* cyanobionts were isolated from the dorsal foliar cavities of 48 *Azolla* accessions (Table 1) using the gentle roller method [54,55] with the following modifications. Roots were cut off, and sporophytes were disinfected in aqueous sodium hypochlorite (1 mL NaClO:10 mL distilled water, *v*:*v*) for 20 min, followed by three washes in ultrapure water (Millipore, Madrid, Spain). Sporophytes were sectioned and squeezed with a roller to separate the cyanobiont from *Azolla* cavities. The extract (*Azolla* + water + *N. azollae*) was centrifuged twice at 3000× *g* for 3 min to settle fern debris. The recovered supernatant with *N. azollae* filaments was centrifuged twice at 1000× *g* for 1 min to free cyanobionts from the cellular debris. The recovered dark green pellet was centrifuged at 11,000× *g* for 10 min, stored at −20 °C, frozen at −80°C, lyophilized, and weighted.

### 4.3. Detection and Analysis of BMAA (β-N-Methylamino-L-Alanine)

#### 4.3.1. Method 1

The methodology, including reagents and materials, described by Baptista et al., (2015) [56] was used., with extraction of BMAA and quantification by LC-MS/MS using validated analytical methods [50,56]. Lyophilized *Azolla* biomass and *N. azollae* isolated from *Azolla* (10 mg each sample) were acid-digested in 6 M HCl at 90 °C for 20 min, using a high-pressure microwave system (Milestone-Ethos 1, Sorisole, Italy). After evaporation with nitrogen, 20 mM of HCl was added to samples and filtered (0.22 μm Millipore, Burlington, MA, USA).

Analyses of BMAA by LC-MS/MS were performed in a Thermo LCQ Fleet Ion Trap LC/MSn system (Thermo Scientific, Waltham, MA, USA) using a 2.1 × 100 mm, 5 μm diameter ZIC-HILIC column (SeQuant, Geneva, Switzerland) and a 14 × 1 mm, 5 μm guard column (SeQuant). The mobile phase was acetonitrile (0.1% formic acid) and deionized water (0.1% formic acid). A linear gradient of 90% acetonitrile for 20 min was followed by 60% acetonitrile for 15 min and 90% acetonitrile for 5 min. The flow rate was 0.5 mL min^−1^, and the injection volume was 10 μL; the column temperature was 40 °C, and we used the positive mode in electrospray ionization (ESI). Nitrogen was the sheath gas at a rate of 45 (unitless) and the auxiliary gas at a rate of 20 (unitless). The capillary temperature was held at 250 °C. A mass-to-charge ratio (*m*/*z*) scan was performed from 50 to 150, and the ion *m*/*z* 119 was monitored to assess 2,4-DAB (2,4-diaminobutyric acid). The occurrence of the product ions *m*/*z* 102, 88 and 76 was verified at a collision energy of 14 V for the presence of BMAA.

#### 4.3.2. Method 2

Method 2 followed that described by Pravadali-Cekic S. et al., (2023) [49] with some modifications for the amount of starting material, chromatographic column, eluents and mode of mass detection. Lyophilized *Azolla* biomass (100 mg) was dissolved in 3 mL of trichloacetic acid (TCA) 10% (*v*/*v*) and sonicated on ice (5 min, 70% amplitude, 20 Hz), followed by overnight precipitation at 4 °C. The mixture was then centrifuged (5000× *g*, 15 min, 4 °C), the supernatant reserved, and the pellet submitted to a second extraction cycle. The third extraction step used 10% TCA/acetone. The pellet as the bound fraction was transferred to a glass vial with acetone (100%), centrifuged, and the supernatant added to the free fraction. The pooled free fraction was then evaporated to dry in a SpeedVac (Büchi, Maia, Portugal) and kept at −80°C. Pellets were also dried using the SpeedVac, and acid hydrolysis was carried out by adding 3 mL of 6 M HCl overnight at 110 °C. The hydrolyzed pellet was re-suspended in 1 mL ultrapure water and added to the free fraction. Samples were then filtered. This was carried out with a 20 µL standard mix solution or sample extract, 20 µL of derivatizing reagent, and 60 µL of borate buffer. Using an AccQ-Tag Ultra Derivatisation Kit in accordance with the manufacturer’s guidelines, the mixture was vortexed for several seconds and placed in a thermocycler at 55 °C for 10 min. The final extract was then transferred to a 1.5 mL vial for LC/MS/MS analysis.

Samples were injected into a liquid chromatograph Thermo Finnigan Surveyor HPLC System (Thermo Scientific, Waltham, MA, USA), coupled with a mass spectrometry LCQ Fleet™ Ion Trap Mass Spectrometer (Thermo Scientific, Waltham, MA, USA). We optimized the parameters of the XcaliburTM version 2 mass spectrometer tune method for data acquisition and processing using direct injection of BMMA and co-occurring isomers in a solution of 1 ppm in LCMS-grade water (Table 2). The mass spectrometer operated in electrospray positive polarity mode using collision ionisation dissociation (CID) corresponding to the [M + H]^+^ BMAA, AEG (N-(2-aminoethyl)-glycine) and 2,4-DAB molecules ion precursors and respective diagnostic fragments. The spray voltage was maintained at 3.5 kV, capillary temperature at 350 °C, and capillary voltage at 20 kV and tube lens at 120 kV. Nitrogen was used as the sheath and auxiliary gas, with collision energy at 20 eV in collision-induced dissociation mode. Separation was achieved on an ACE Excel C18 (50 × 2.1 mm I.D., 1.7 μm, Batch: V17-1253, Avantor^®^, ACE^®^, VWR, PT) at 18 °C, with a flow rate of 0.3 mL/min injected at a volume of 10 μL in no-waste mode. The eluents used were methanol (A) and water (B), both acidified with formic acid at 0.1% (*v*/*v*). The gradient program started at 13% A, increasing to 90% A in 20 min before turning back to initial conditions in 5 min and equilibrating for an additional 10 min with 20% A. See Table 2 for the chromatographic and mass parameters.

### 4.4. Cyanotoxin Genes in Azolla Accessions and Nostoc Azollae

#### DNA Extraction

DNA from *N. azollae* isolated from *Azolla* accessions was extracted with a PureLink^®^ Genomic DNA MiniKit (Invitrogen, Carlsbad, CA, USA), and DNA from *Azolla* accessions was extracted with Genomic DNA from Plant NucleoSpin^®^ Plant II (Macherey-Nagel, Düren, Germany) according to the manufacturer’s instructions. DNA was stored at −20 °C. The DNA was quantified in a Qubit fluorometer (Invitrogen) using the Quant-iT^®^ dsDNA HS assay following the manufacturer’s instructions. A working DNA concentration of 0.1 μg/μL was made with sterile ultrapure water, and saxitoxin, nodularin, and microcystin were assessed by specific primers (Table 3). A Biometra TProfessional (Goettingen, Germany) thermocycler was used for PCR amplification using the conditions listed in Table 4 for each gene, with a hold at 4 °C for all the programs. Each 20 μL reaction contained 1 μL of 0.5 µM of each primer (Invitrogen, Waltham, MA, USA), 2 μL of 0.1 μg/μL DNA, 9 μL Supreme NZYTaq 2x Green Master Mix (NZYTech, Lisbon, Portugal), and 7 μL of ultrapure sterile water. Negative (with sterile ultrapure water) and positive (*Microcystis aeruginosa* LEGE91094 for microcystin and microcystin/nodularin genes, *Aphanizomenon ovalisporum* for the cylindrospermopsin gene, and *Aphanizomenon gracillaris* LMECYA 40 from INSA for the saxitoxin gene) controls were included. The amplification products were separated via 1.5% agarose gel electrophoresis running in TAE 1× at 150 V for 25–30 min and stained with 0.2 μg/mL ethidium bromide (BioRad, Hercules, CA, USA). The 1 Kb Plus DNA ladder (Invitrogen) was used as a molecular size marker.

**Table 3:** Primers used to amplify cyanotoxic genes in *Azolla* and *N. azollae* DNA.

Gene	Primer	Sequence Primer (5′ → 3′)	Size (bp)	Reference
Saxitoxin *(sxt)*	SXT683F	GGATCTCAAACATGATCCCA	195	[57]
	SXT877R	GCCAAACGCAGTACCACTT		
Cylindrospermopsin (*cyl*) (poliketide synthase)	K18F	CCTCGCACATAGCCATTTGC	422	[58]
	M4R	GAAGCTCTGGAATCCGGTAA		
Cylindrospermopsin (*cyl*) (peptide synthase)	M13	GGCAAATTGTGATAGCCACGAGC	597	[59]
	M14	GATGGAACATCGCTCACTGGTG		[58]
Microcystin/Nodularin synthetase (*mcyE*/*ndaF*)	HepF	TTTGGGGTTAACTTTTTTGGCCATAGTC	472	[60]
	HepR	AATTCTTGAGGCTGTAAATCGGGTTT		
Microcystin synthetase (*mcy A*)	mcyA-Cd1F	AAAATTAAAAGCCGTATCAAA	297	[61]
	mcyA-Cd1R	AAAAGTGTTTTATTAGCGGCTCAT		
Microcystin synthetase (*mcy B*)	2959F	TGGGAAGATGTTCTTCAGGTATCCAA	350	[62]
	3278R	AGAGTGGAAACAATATGATAAGCTAC		
Microcystin (*mcy C*)	FAA	CTATGTTATTTATACATCAGG	758	[63]
	RAA	CTCAGCTTAACTTGATTATC		
Microcystin (*mcy B*, domain A)	2156F	ATCACTTCAATCTAACGACT	955	[64]
	3111R	GTTGCTGCTGTAAGAAA		
Microcystin (*mcy C*, domain A)	PSCF1	GCAACATCCCAAGAGCAAAG	674	[65]
	PSCR1	CCGACAACATCACAAAGGC		
Microcystin (*mcy D*, domain ACP)	PKDF1	GACGCTCAAATGATGAAACT	647	[65]
	PKDR1	GCAACCGATAAAAACTCCC		
Microcystin (*mcy D*, domain KS)	PKDF2	AGTTATTCTCCTCAAGCC	859	[65]
	PKDR2	CATTCGTTCCACTAAATCC		
Microcystin (*mcy E*, domain GSA-AMT)	PKEF1	CGCAAACCCGATTTACAG	755	[65]
	PKER1	CCCCTACCATCTTCATCTTC		
Microcystin (*mcy G*, domain CM)	PKGF1	ACTCTCAAGTTATCCTCCCTC	425	[65]
	PKGR1	AATCGCTAAAACGCCACC		

**Table 4:** Amplification conditions for the cyanotoxic genes in *Azolla* and *N. azollae* DNA.

Gene	Initial Denaturation	Denaturation	Annealing	Extension	Final Extension	Reference
*sxt*	94 °C; 3 min	35 cycles			72 °C; 7 min	[57]
		94 °C; 10 s	52 °C; 20 s	72 °C; 1 min		
*cyl*	94 °C; 10 min	30 cycles			72 °C; 7 min	[59]
		94 °C; 30 s	55 °C; 30 s	72 °C; 7 min		
*mcyE/ndaF*	92 °C; 2 min	35 cycles			72 °C; 5 min	[60]
		92 °C; 20 s	56 °C; 30 s	72 °C; 1 min		
*mcy A*	95 °C; 2 min	35 cycles			72 °C; 7 min	[61]
		95 °C; 90 s	56 °C; 30 s	72 °C; 50 s		
*mcy B*	94 °C; 2 min	35 cycles			72 °C; 5 min	[62]
		94 °C; 30 s	59 °C; 45 s	72 °C; 1 min		
*mcy C*	94 °C; 2 min	35 cycles			72 °C; 7 min	[63]
		94 °C; 10 s	50 °C; 20 s	72 °C; 1 min		
*mcy B*, domain A	94 °C; 4 min	30 cycles			72 °C; 7 min	[64]
		95 °C; 30 s	52 °C; 30 s	72 °C; 1 min		
*mcy C*, domain A	94 °C; 5 min	35 cycles			72 °C; 7 min	[65]
		95 °C; 1 min	52 °C; 30 s	72 °C; 1 min		
*mcy D*, domain ACP	94 °C; 5 min	35 cycles			72 °C; 7 min	[65]
		95 °C; 1 min	52 °C; 30 s	72 °C; 1 min		
*mcy D,* domain KS	94 °C; 5 min	35 cycles			72 °C; 7 min	[65]
		95 °C; 1 min	52 °C; 30 s	72 °C; 1 min		
*mcy E*, domain GST-AMT	94 °C; 5 min	35 cycles			72 °C; 7 min	[65]
		95 °C; 1 min	52 °C; 30 s	72 °C; 1 min		
*mcy G*, domain CM	94 °C; 5 min	35 cycles			72 °C; 7 min	[65]
		95 °C; 1 min	52 °C; 30 s	72 °C; 1 min		

### 4.5. BLAST of Anatoxin-a Genes against Nostoc azollae

To determine if *N. azollae* produces anatoxin-a, a nucleotide BLAST (BLASTN) search was performed for anatoxin-a coding genes. Since the anatoxin-a gene cluster was discovered after the PCR and gel analysis of the other cyanotoxins performed in this study, analysis of its presence was carried out separately through BLAST rather than as the query sequence [66]. This was a 34,682 bp sequence encoding for proteins associated with the biosynthesis of these toxins. The nucleotide query was applied to the full genome of *Nostoc azollae* 0708 (taxid: 551115). Matches with E-values from the BLASTN of less than 0.01 were investigated and analyzed.

## 5. Conclusions

Our LC-MS/MS results show the *Azolla–Nostoc azollae* superorganism does not contain BMAA or their isomers DAB and AEG and that *Azolla* and *N. azollae* do not synthesize other common cyanotoxins, indicating that *Azolla* is a nutritious food that can be safely eaten.

## Figures and Tables

**Table 1:** List of *Azolla* accessions from countries worldwide.

Accession ^a^	Species Name	Origin and Harvest Year	Source ^b^/ Collector
PI1 *^,$^	*A. pinnata* subsp. *imbricata*	Philippines, Sto Domingo, Albay, 1975	IRRI
**PI2**		Malaysia, Bumbong Lima, Butterworth, 1977	IRRI
**PI23**		India, Cuttack, Orissa, 1978	CRRI
**PI68**		Sri Lanka, Tissa, 1984	S. Kulasooriya
**PI102**		Japan, Okinawa, 1987	O. Mochida
**PI503**		Australia, Murdoch, 1978	M. Dilworth
**PI531**		Indonesia, Bali, 1983	-
**PI540**		China, Putian, 1989	C. van Hove
FI1001 *	*A. filiculoides*	East Germany (ex-GDR), 1979	IB China
**FI1008**		USA, Cranmore Road, Sutter Co., California, 1981	D. Rains
**FI1010**		Peru, PUFFI, Lima, 1982	CIAT
**FI1042**		Brazil, Parana, 1987	I. Watanabe
**FI1052**		South of France, North of Lyon, 1989	P. Roger
**FI1090**		Japan, Tanabe-cho, 1992	S. Kitoh
**FI1501**		Belgian, Harchies, 1987	A. Lawalree
**FI1505**		South Africa, Verwoerd dam, 1987	D. Toerien
**FI1507 ^$^**		Colombia, Zipaquira, 1987	Y. Lopez
**FI1522**		Switzerland, Zurich Botanical Garden, 1987	-
**FI-BGLU**		Botanical Garden of Lisbon University, 2009	A.L. Pereira
**FI-BGM**		Botanical Garden of Madeira, Funchal, 2010	C. Lobo
ME2001 *	*A. mexicana*	USA, Graylodge, California, 1978	D. Rains
**ME2008**		Colombia, CIAT, Cali, 1982	CIAT
**ME2011**		Japan, Osaka, 1984	T. Lumpkin
**ME2026 ^$^**		Brazil, Solimoes river, Pacencia Island, Iranduba, Amazonas (BLCC 18), 1984	T. Lumpkin
CA3001 *^,$^	*A. caroliniana*	USA, Ohio, 1978	D. Rains
**CA3017**		Brazil, Rio Grande do Sul, 1987	I. Watanabe
**CA3502**		Egypt, Moshtohr University, 1987	C. Myttenaere
**CA3507**		Suriname, Boxel, 1987	H. Lardinois
**CA3513**		Zimbabwe, Causeway Botanical Garden, 1987	T. Muller
**CA3524**		Holland, 1987	E. Ohoto
**CA3525**		Ruanda, Cyili Rice Research Center, 1987	C. van Hove
MI4018 *	*A. microphylla*	Paraguay, 1981	D. Rains
**MI4021 ^$^**		Equator, Santa Cruz Island, Galapagos, 1982	T. Lumpkin
**MI4028**		Philippines, hybrid (MI4018xFI1001), 1985	Do Van Cat
**MI4054**		Brazil, Baía, 1987	I. Watanabe
**MI4510**		Philippines, Los Baños, IRRI, 1987	C. van Hove
NI5001 *^,$^	*A. nilotica*	Sudan, Kosti, 1982	T. Lumpkin
**NI5002 ^#^**		Sudan, Kosti, 1989	T. Lumpkin
**NI5501**		Burundi, Bujumbura, 1987	J. Bouharmont
RU6010 *	*A. rubra*	New Zealand, Nouville, 1986	C. van Hove
**RU6502**		Australia, Victoria (37.40 S–144.40 E), 1985	-
**RU6503**		New Zealand, between Lumdsen and Kingston, 1986	C. van Hove
PP7001 *^,$^	*A. pinnata* subsp. *pinnata*	Australia, Kakadu Northern Park Northern Territory, 1982	Yatazawa
**PP7506**		Sierra Leone, 1982	C Dixon
**PP7509**		Nigeria, Moor plantation, 987	C. van Hove
**PP7511**		Guinea-Bissau, Contuboel, 1987	H. Diara
**PP7512**		Zaire, Kisantu, 1987	B. Bruyneel
**PP7546**		Madagascar, Antsahavory, East zone, 1991	C. van Hove

^a^ Accession numbers are listed according to IRRI code number except for Portuguese accessions (FI-BGLU and FI-BGM) from an unknown collector or germplasm source. ^b^ CIAT: International Centre for Tropical Agriculture, Colombia; CRRI: Cyili Rice Research Center; IB China: Institute of Botany, Academia Sinica, Beijing, China; IRRI: International Rice Research Institute. ^#^ *N. azollae* was not isolated from this *Azolla* accession. * BMAA extracted from *Azolla* and *Nostoc azollae* (isolated from *Azolla*, see Section 4.2) with method 1 (see Section 4.3.1). ^$^ BMAA extracted from *Azolla* accessions with method 2 (see Section 4.3.2).

**Table 2:** Chromatographic and mass parameters for the BMAA and the detection of isomers AEG and DAB.

Target	Retention Time (min)	Derivatized Ion Precursor (*m*/*z*)	CID Fragments (*m*/*z*)	CID Colission Energy (V)	Calibration: Curve and Linear Interval (µg/L)	LOD (µg/L)	LOQ (µg/L)
**BMAA**	13.92 ± 0.08	459.00	320, 315, 289, **258**, 171, 145	35	y = 1377.4x − 2154.1 r^2^ = 0.9947 3-250	7.99	24.2
**AEG**	13.22 ± 0.11	459.00	320, 315, 289, **272**, 171, 145	35	y = 889.91x + 4972.2 r^2^ = 0.9993 3-150	57.4	174
**2,4-DAB**	14.93 ± 0.15	459.00	320, 315, 289, **271**, 171, 145	35	y = 2785x + 5538.1 r^2^ = 0.9934 7-250	70	212

LOD—limit of detection; LOQ—limit of quantification; CID—collision-induced dissociation.

## Data Availability

The original contributions presented in the study are included in the article/supplementary material.

## Supplementary Materials

The following supporting information can be downloaded at https://www.mdpi.com/article/10.3390/plants13192707/s1, Supplementary Materials 1, Figures S1–S24: Agarose gel from the PCR amplification of genes in *Azolla* and *N. azollae*. Figure S25: Map showing the countries of origin of *Azolla* accessions used in the present study. Figure S1: Agarose gel from the PCR amplification of the gene microcystin/nodularin synthetase for nodularin in 48 *Azolla* accessions (see Table 1 from the manuscript). C+: positive control (*M. aeruginosa* LEGE 91094), C−: negative control. The first line is the ladder. Figure S2: Agarose gel from the PCR amplification of the gene saxitoxin in 48 *Azolla* accessions (see Table 1 from the manuscript). C+: positive control (*A. gracillaris* LMECYA 40), C−: negative control; the first line is the ladder. Figure S3: Agarose gel from the multiplex PCR amplification of the genes poliketide synthase and peptide synthase for cylindrospermopsin in 48 *Azolla* accessions (see Table 1 from the manuscript). C+: positive control (*A. ovalisporum*), C−: negative control; the first line is the ladder. Figure S4: Agarose gel from the PCR amplification of the gene microcystin synthetase (mcy A) for microcystin in 48 *Azolla* accessions (see Table 1 from the manuscript). C+: positive control (*M. aeruginosa* LEGE 91094), C−: negative control; the first line is the ladder. Figure S5: Agarose gel from the PCR amplification of the gene microcystin synthetase (mcy B) for microcystin in 48 *Azolla* accessions (see Table 1 from the manuscript). C+: positive control (*M. aeruginosa* LEGE 91094), C−: negative control; the first line is the ladder. Figure S6: Agarose gel from the PCR amplification of the gene microcystin C for microcystin in 48 *Azolla* accessions (see Table 1 from the manuscript). C+: positive control (*M. aeruginosa* LEGE 91094), C−: negative control; the first line is the ladder. Figure S7: Agarose gel from the PCR amplification of the gene microcystin B A-domain for microcystin in 48 *Azolla* accessions (see Table 1 from the manuscript). C+: positive control (*M. aeruginosa* LEGE 91094), C−: negative control; the first line is the ladder. Figure S8: Agarose gel from the PCR amplification of the gene microcystin C A-domain for microcystin in 48 *Azolla* accessions (see Table 1 from the manuscript). C+: positive control (*M. aeruginosa* LEGE 91094), C−: negative control; the first line is the ladder. Figure S9: Agarose gel from the PCR amplification of the gene microcystin D ACP-domain for microcystin in 48 *Azolla* accessions (see Table 1 from the manuscript). C+: positive control (*M. aeruginosa* LEGE 91094), C−: negative control; the first line is the ladder. Figure S10: Agarose gel from the PCR amplification of the gene microcystin D KS-domain for microcystin in 48 *Azolla* accessions (see Table 1 from the manuscript). C+: positive control (*M. aeruginosa* LEGE 91094), C−: negative control; the first line is the ladder. Figure S11: Agarose gel from the PCR amplification of the gene microcystin E GSA-AMT-domain for microcystin in 48 *Azolla* accessions (see Table 1 from the manuscript). C+: positive control (*M. aeruginosa* LEGE 91094), C−: negative control; the first line is the ladder. Figure S12: Agarose gel from the PCR amplification of the gene microcystin G CM-domain for microcystin in 48 *Azolla* accessions (see Table 1 from the manuscript). C+: positive control (*M. aeruginosa* LEGE 91094), C−: negative control; the first line is the ladder. Figure S13: Agarose gel from the PCR amplification of the gene microcystin/nodularin synthetase for nodularin in 47 *Nostoc azollae* isolated from *Azolla* accessions (see Table 1 from the manuscript). C+: positive control (*M. aeruginosa* LEGE 91094), C−: negative control; the first line is the ladder. Figure S14: Agarose gel from the PCR amplification of the gene saxitoxin in 47 *Nostoc azollae* isolated from *Azolla* accessions (see Table 1 from the manuscript). C+: positive control (*A. gracillaris* LMECYA 40), C−: negative control; the first line is the ladder. Figure S15: Agarose gel from the multiplex PCR amplification of the genes poliketide synthase and peptide synthase for cylindrospermopsin in 47 *Nostoc azollae* isolated from *Azolla* accessions (see Table 1 from the manuscript). C+: positive control (*A. ovalisporum*), C−: negative control; the first line is the ladder. Figure S16: Agarose gel from the PCR amplification of the gene microcystin synthetase (mcy A) for microcystin in 47 *Nostoc azollae* isolated from *Azolla* accessions (see Table 1 from the manuscript). C+: positive control (*M. aeruginosa* LEGE 91094), C−: negative control; the first line is the ladder. Figure S17: Agarose gel from the PCR amplification of the gene microcystin synthetase (mcy B) for microcystin in 47 *Nostoc azollae* isolated from *Azolla* accessions (see Table 1 from the manuscript). C+: positive control (*M. aeruginosa* LEGE 91094), C−: negative control; the first line is the ladder. Figure S18: Agarose gel from the PCR amplification of the gene microcystin C for microcystin in 47 *Nostoc azollae* isolated from *Azolla* accessions (see Table 1 from the manuscript). C+: positive control (*M. aeruginosa* LEGE 91094), C−: negative control; the first line is the ladder. Figure S19: Agarose gel from the PCR amplification of the gene microcystin B A-domain for microcystin in 47 *Nostoc azollae* isolated from *Azolla* accessions (see Table 1 from the manuscript). C+: positive control (*M. aeruginosa* LEGE 91094), C−: negative control; the first line is the ladder. Figure S20: Agarose gel from the PCR amplification of the gene microcystin C A-domain for microcystin in 47 *Nostoc azollae* isolated from *Azolla* accessions (see Table 1 from the manuscript). C+: positive control (*M. aeruginosa* LEGE 91094), C−: negative control; the first line is the ladder. Figure S21: Agarose gel from the PCR amplification of the gene microcystin D ACP-domain for microcystin in 47 *Nostoc azollae* isolated from *Azolla* accessions (see Table 1 from the manuscript). C+: positive control (*M. aeruginosa* LEGE 91094), C−: negative control; the first line is the ladder. Figure S22: Agarose gel from the PCR amplification of the gene microcystin D KS-domain for microcystin in 47 *Nostoc azollae* isolated from *Azolla* accessions (see Table 1 from the manuscript). C+: positive control (*M. aeruginosa* LEGE 91094), C−: negative control; the first line is the ladder. Figure S23: Agarose gel from the PCR amplification of the gene microcystin E GSA-AMT-domain for microcystin in 47 *Nostoc azollae* isolated from *Azolla* accessions (see Table 1 from the manuscript). C+: positive control (*M. aeruginosa* LEGE 91094), C−: negative control; the first line is the ladder. Figure S24: Agarose gel from the PCR amplification of the gene microcystin G CM-domain for microcystin in 47 *Nostoc azollae* isolated from *Azolla* accessions (see Table 1 from the manuscript). C+: positive control (*M. aeruginosa* LEGE 91094), C−: negative control; the first line is the ladder. Figure S25: Map showing the countries of origin of *Azolla* accessions used in the present study. Legend: a—*A. pinnata* subsp. *imbricata*; b—*A. filiculoides*; c—*A. mexicana*; d—*A. caroliniana*; e—*A. microphylla*; f—*A. nilotica*; g—*A. rubra*; h—*A. pinnata* subsp. *pinnata.* Supplementary Materials 2: Sequence alignment from the BLASTN query between the Anatoxin-a gene cluster and the *Nostoc azollae* genome.
