# Supplementary material for: Azolla as a Safe Food: Suppression of Cyanotoxin-Related Genes and Cyanotoxin Production in Its Symbiont, Nostoc azollae
Source: Plants (Basel). 2024 Sep 27;13(19):2707. doi: 10.3390/plants13192707 (PMC11479175; doi:10.3390/plants13192707)
Supplement: Supplementary file 1 [file plants-13-02707-s001.zip › plants-3195843-supplementary.pdf]

Supplementary Materials

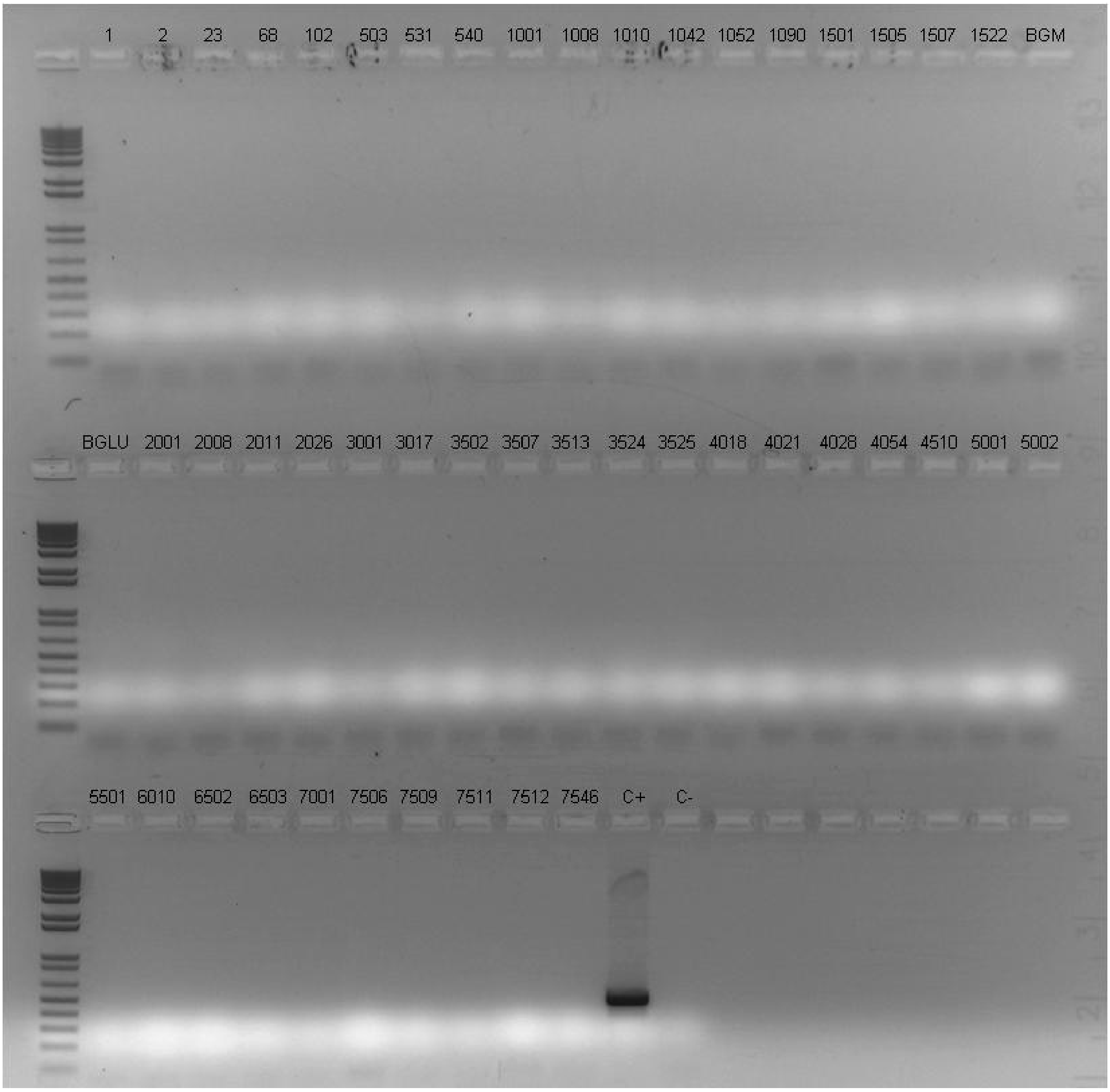

Figure S1: Agarose gel from the PCR amplification of the gene microcystin/nodularin synthetase for nodularin in 48 *Azolla* accessions (see table 3 from the manuscript). C+ -: positive control (*M. aeruginosa* LEGE 91094), C- : negative control, 1st line is Ladder

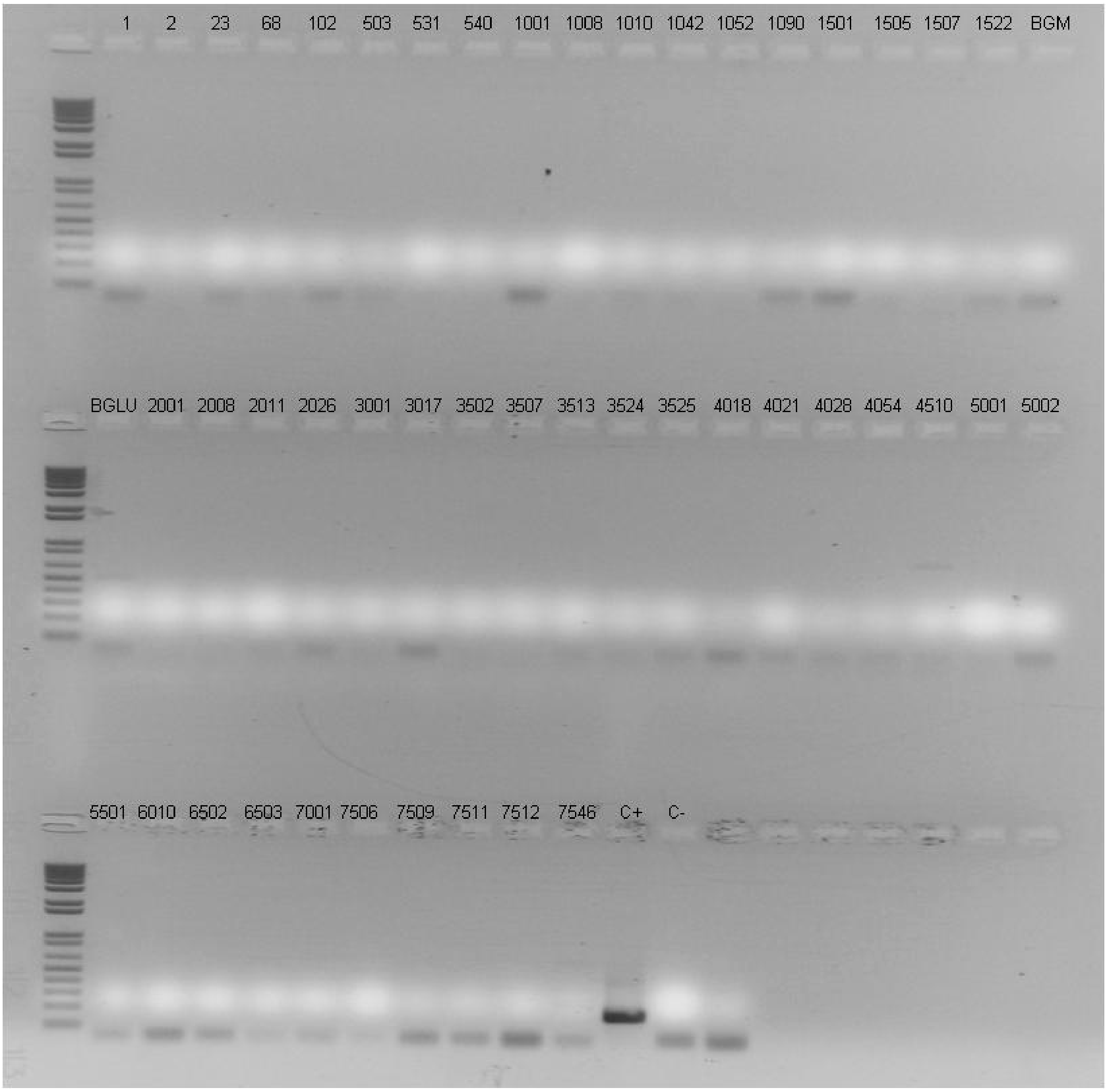

Figure S2: Agarose gel from the PCR amplification of the gene saxitoxin in 48 *Azolla* accessions (see table 3 from the manuscript). C+ -: positive control (*A. gracillaris* LMECYA 40), C- : negative control, 1st line is Ladder

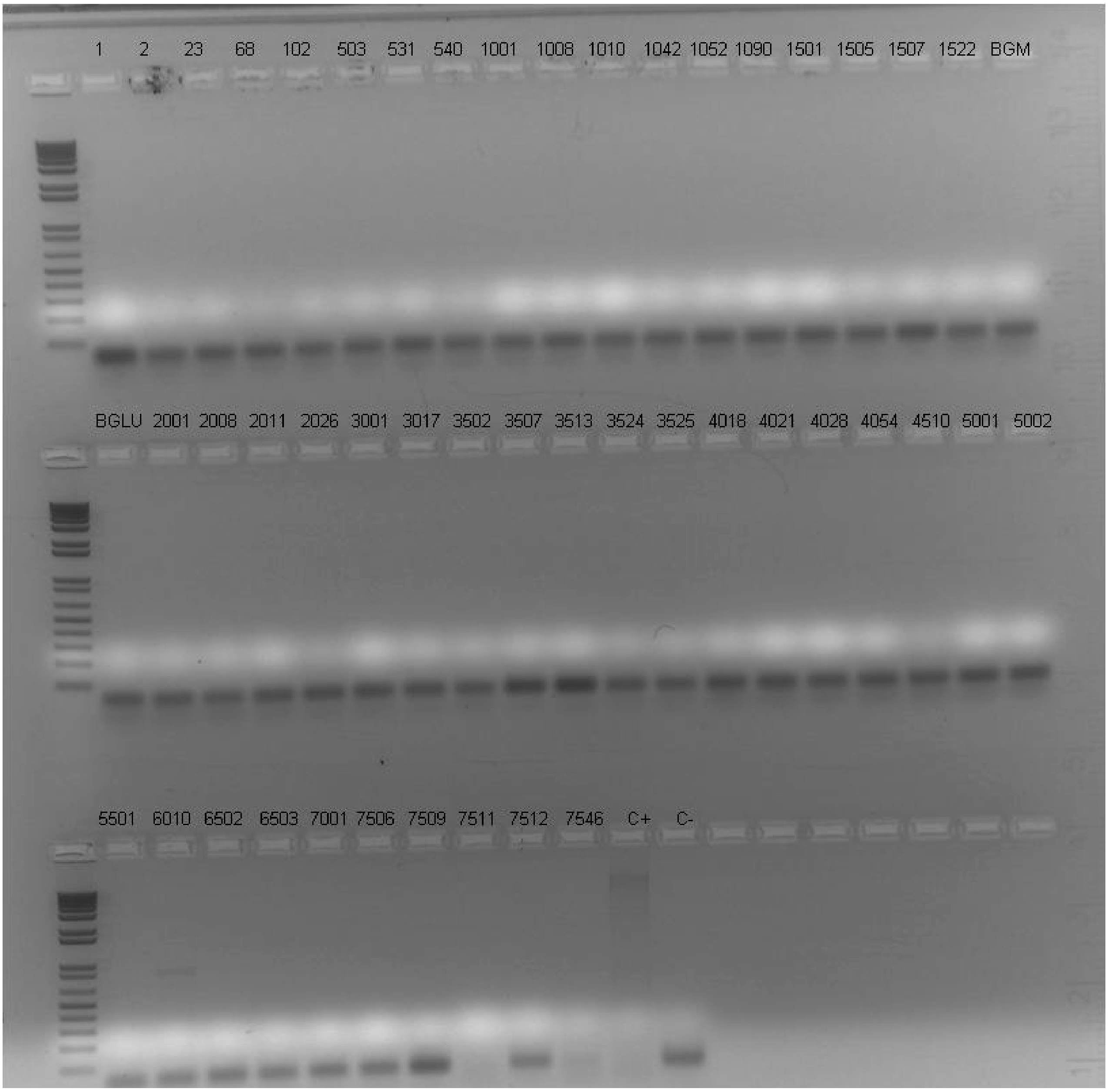

Figure S3: Agarose gel from the multiplex PCR amplification of the genes poliketide synthase and peptide synthase for cylindrospermopsin in 48 *Azolla* accessions (see table 3 from the manuscript). C+ -: positive control (*A. ovalisporum*), C- : negative control, 1st line is Ladder

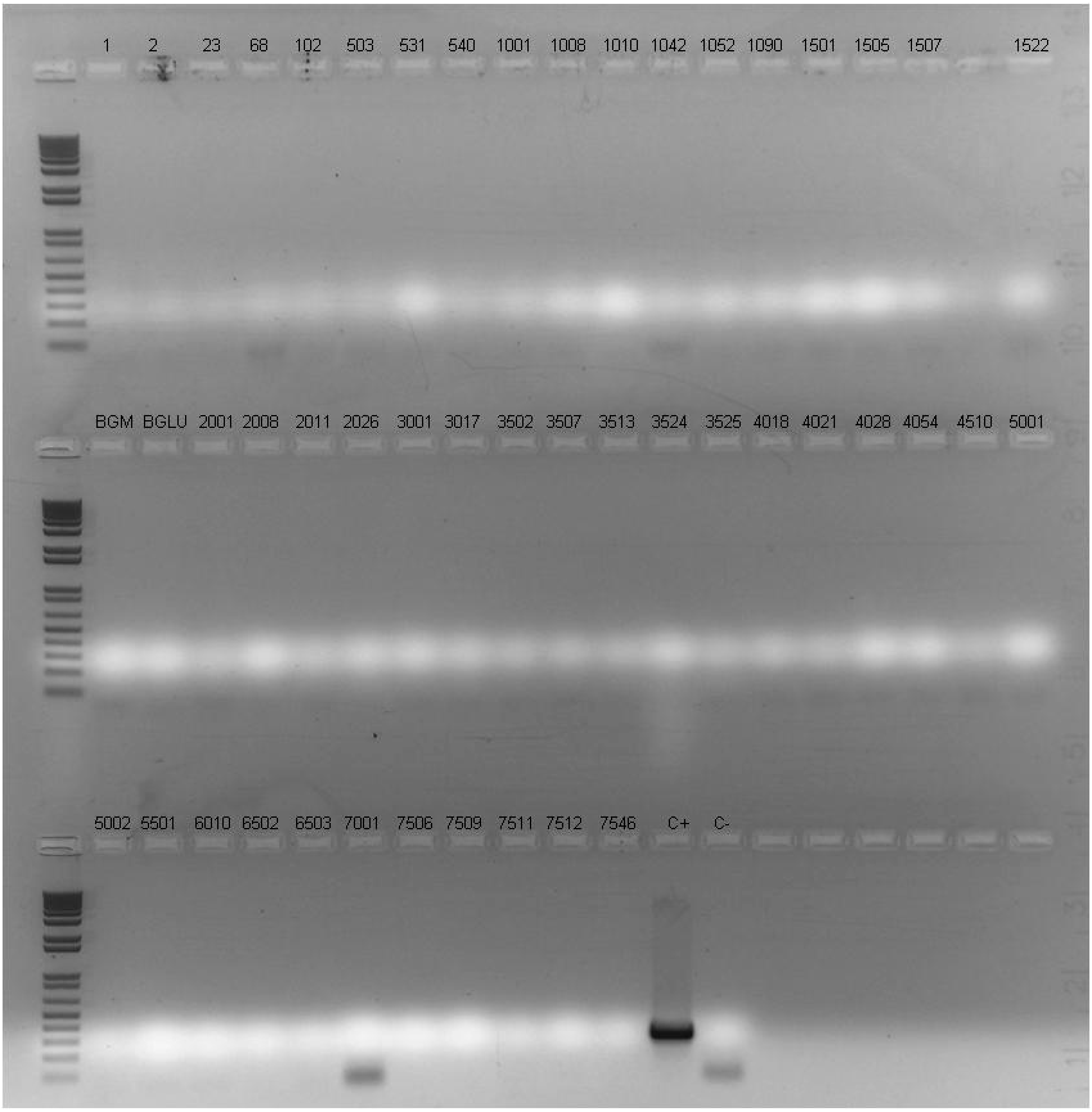

Figure S4: Agarose gel from the PCR amplification of the gene microcystin synthetase (mcy A) for microcystin in 48 *Azolla* accessions (see table 3 from the manuscript). C+ -: positive control (*M. aeruginosa* LEGE 91094), C- : negative control, 1st line is Ladder

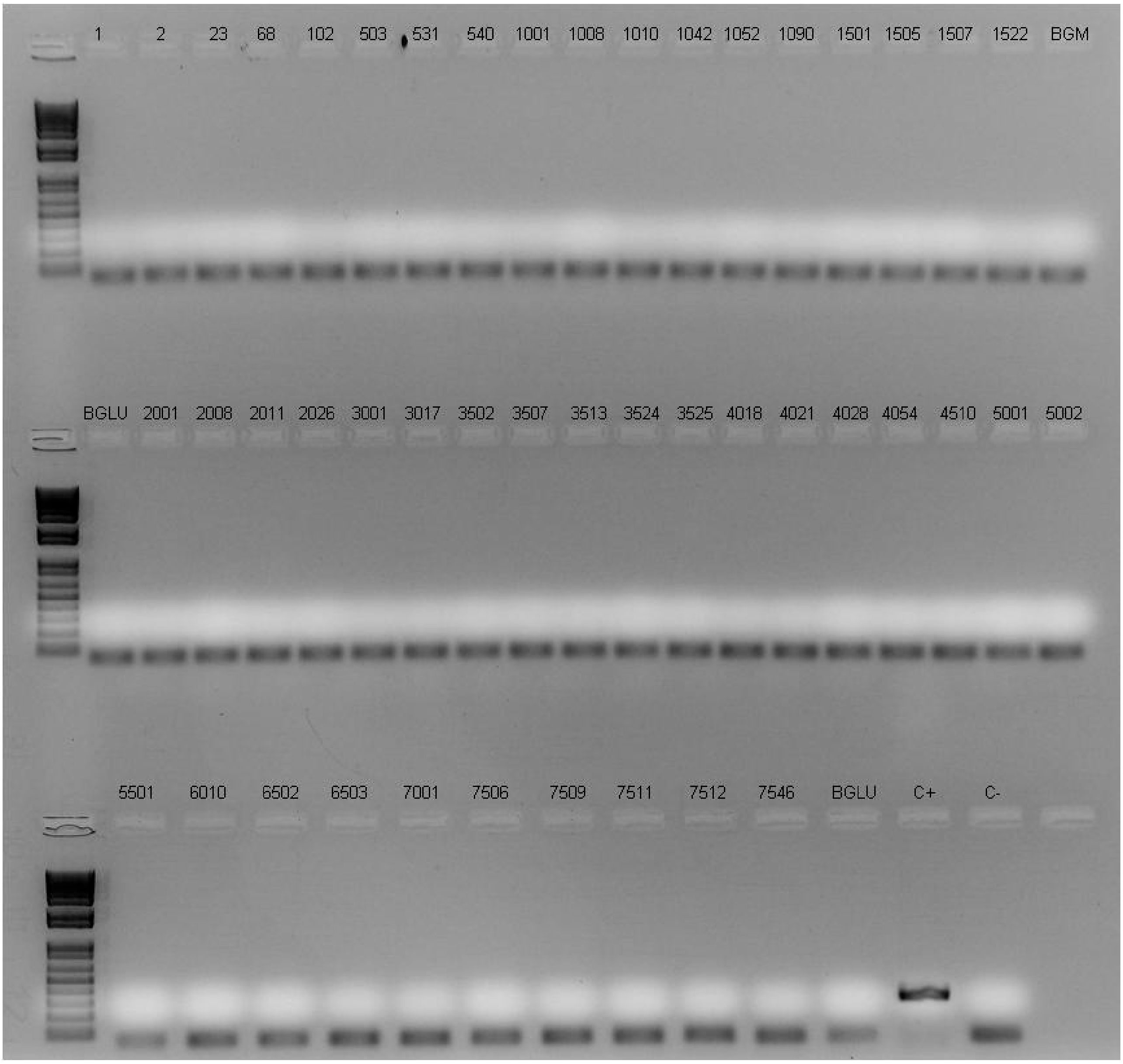

Figure S5: Agarose gel from the PCR amplification of the gene microcystin synthetase (mcy B) for microcystin in 48 *Azolla* accessions (see table 3 from the manuscript). C+ -: positive control (*M. aeruginosa* LEGE 91094), C- : negative control, 1st line is Ladder

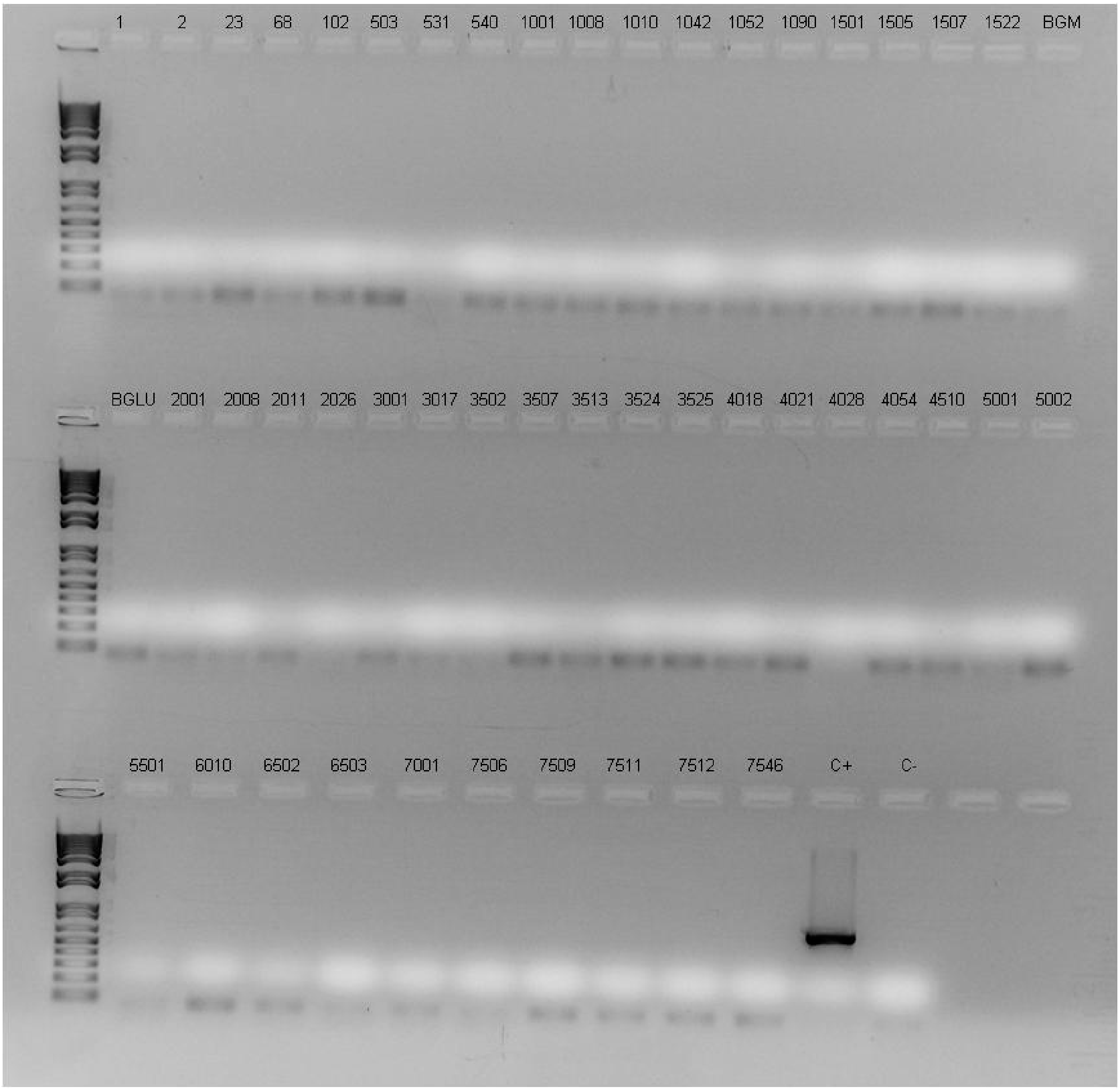

Figure S6: Agarose gel from the PCR amplification of the gene microcystin C for microcystin in 48 *Azolla* accessions (see table 3 from the manuscript). C+ -: positive control (*M. aeruginosa* LEGE 91094), C- : negative control, 1st line is Ladder

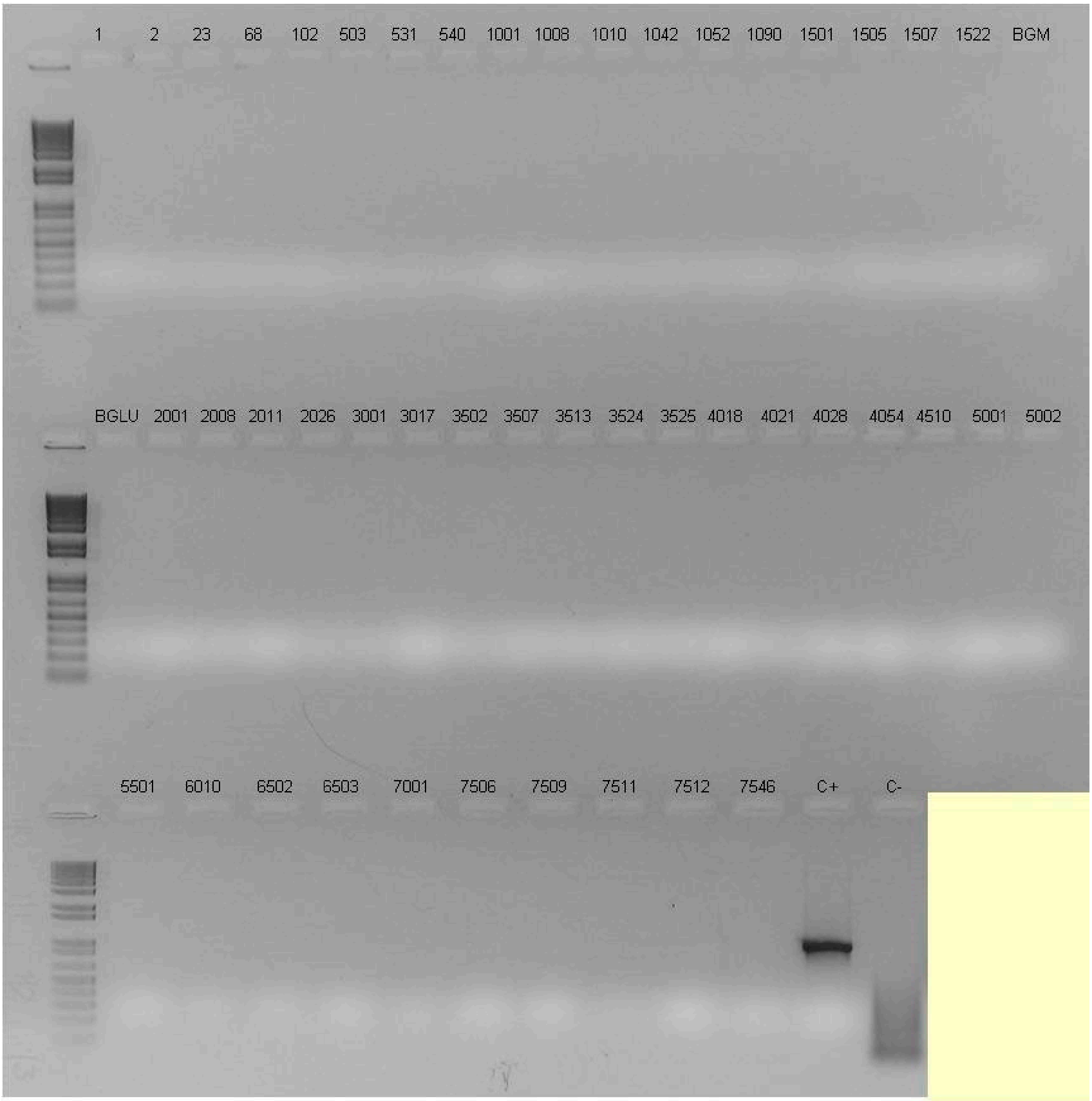

Figure S7: Agarose gel from the PCR amplification of the gene microcystin B A-domain for microcystin in 48 *Azolla* accessions (see table 3 from the manuscript). C+ -: positive control (*M. aeruginosa* LEGE 91094), C- : negative control, 1st line is Ladder

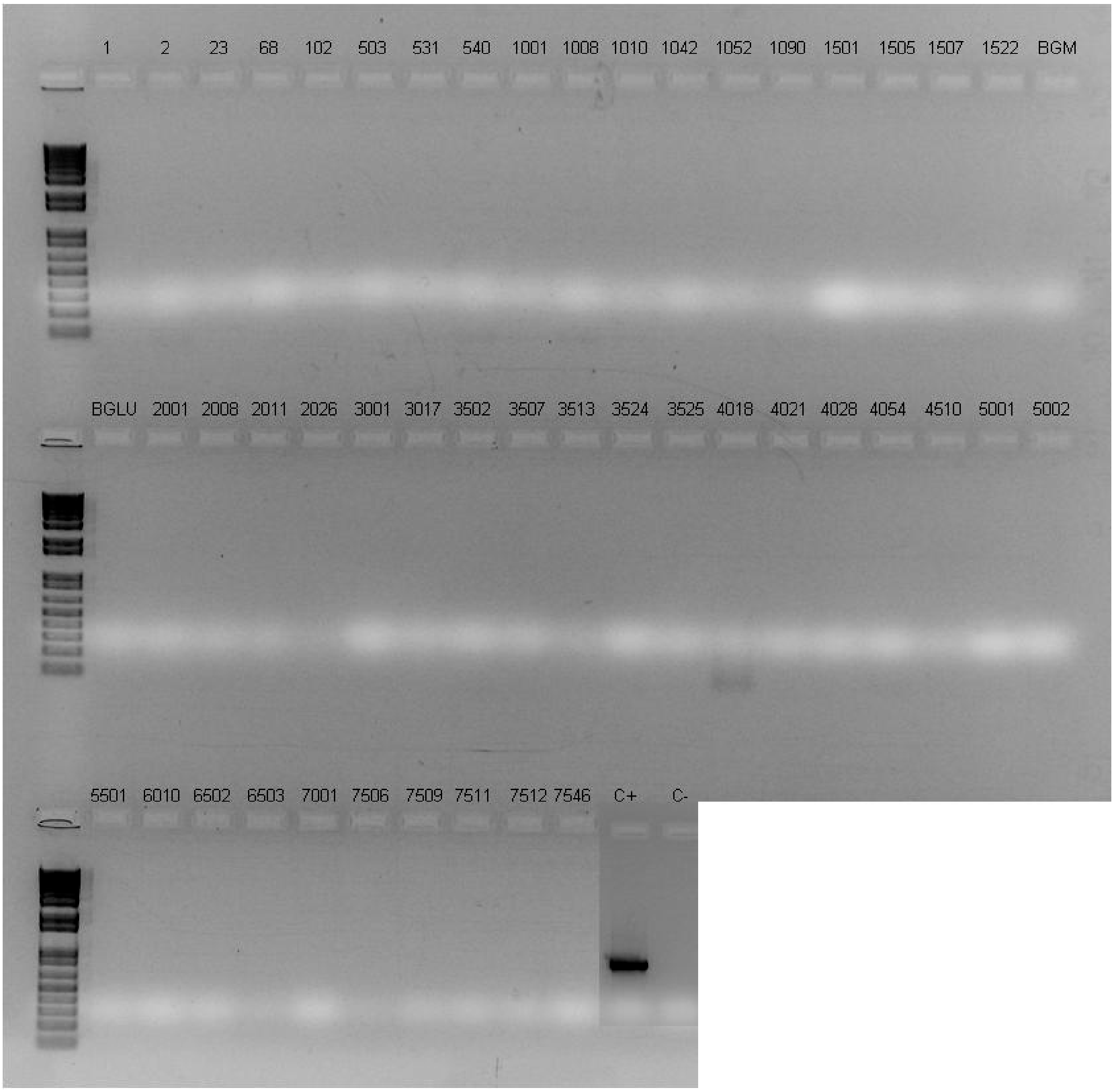

Figure S8: Agarose gel from the PCR amplification of the gene microcystin C A-domain for microcystin in 48 *Azolla* accessions (see table 3 from the manuscript). C+ -: positive control (*M. aeruginosa* LEGE 91094), C- : negative control, 1st line is Ladder

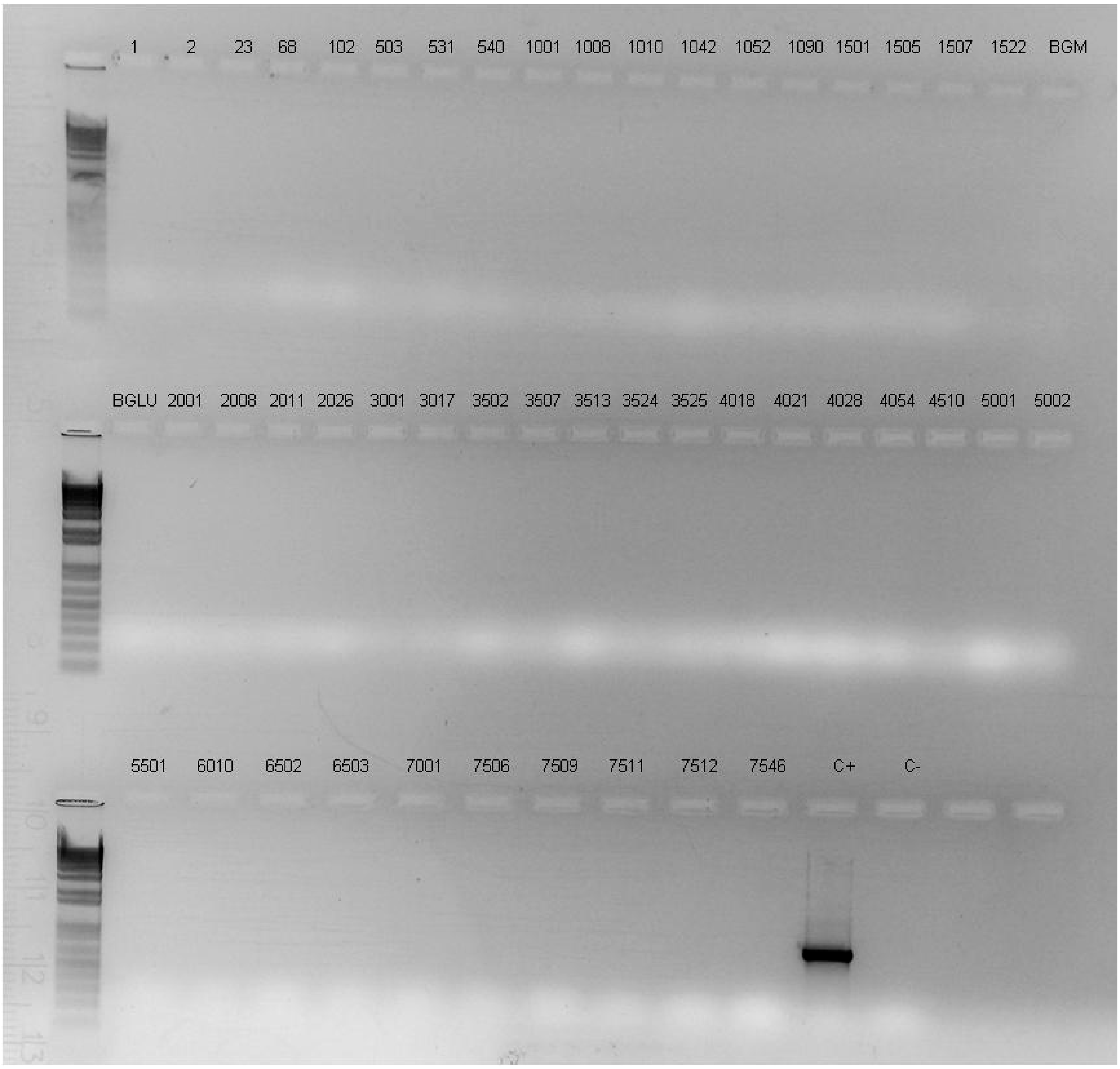

Figure S9: Agarose gel from the PCR amplification of the gene microcystin D ACP-domain for microcystin in 48 *Azolla* accessions (see table 3 from the manuscript). C+ -: positive control (*M. aeruginosa* LEGE 91094), C-: negative control, 1st line is Ladder

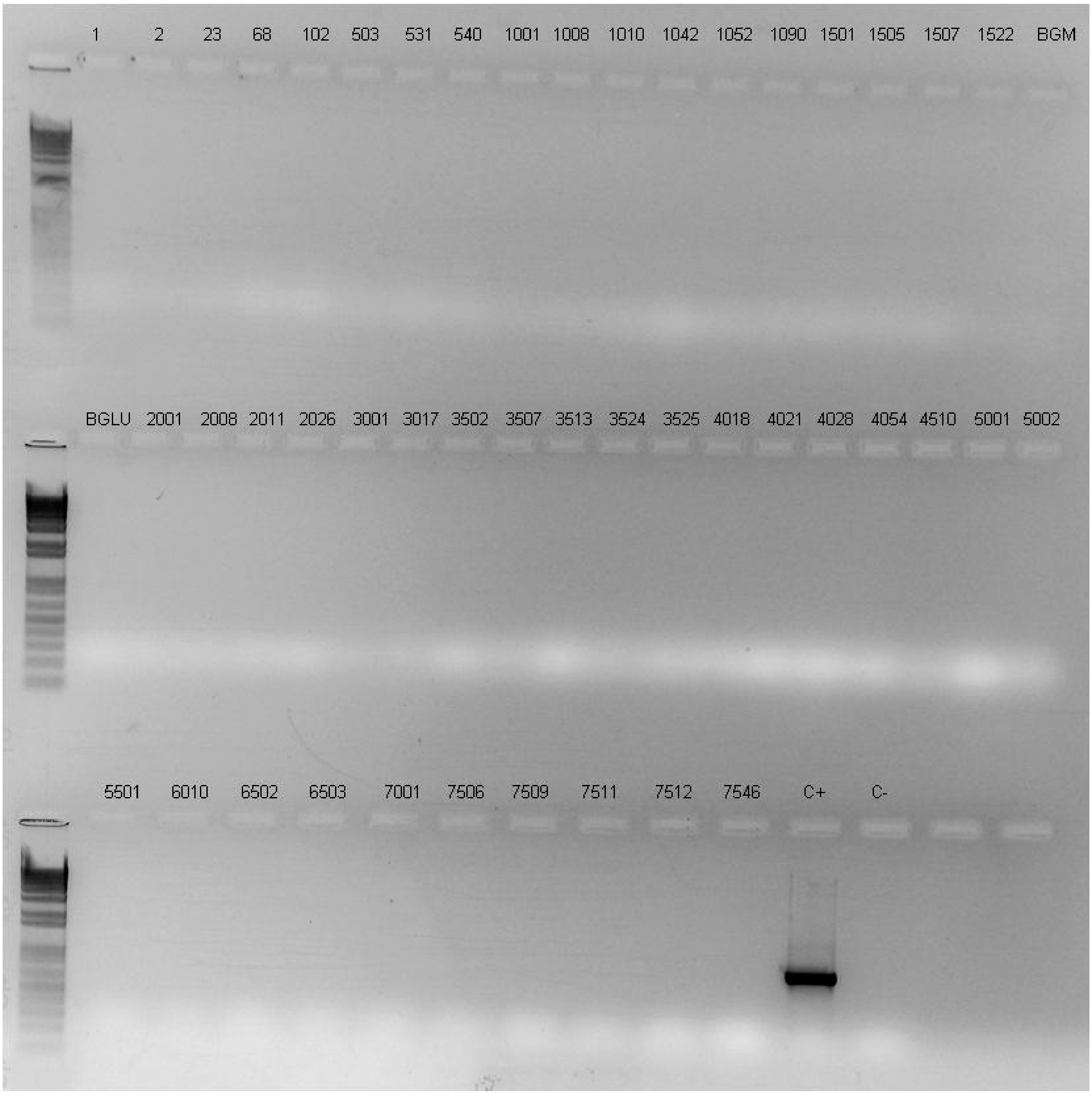

Figure S10: Agarose gel from the PCR amplification of the gene microcystin D KS-domain for microcystin in 48 *Azolla* accessions (see table 3 from the manuscript). C+ -: positive control (*M. aeruginosa* LEGE 91094), C- : negative control, 1st line is Ladder

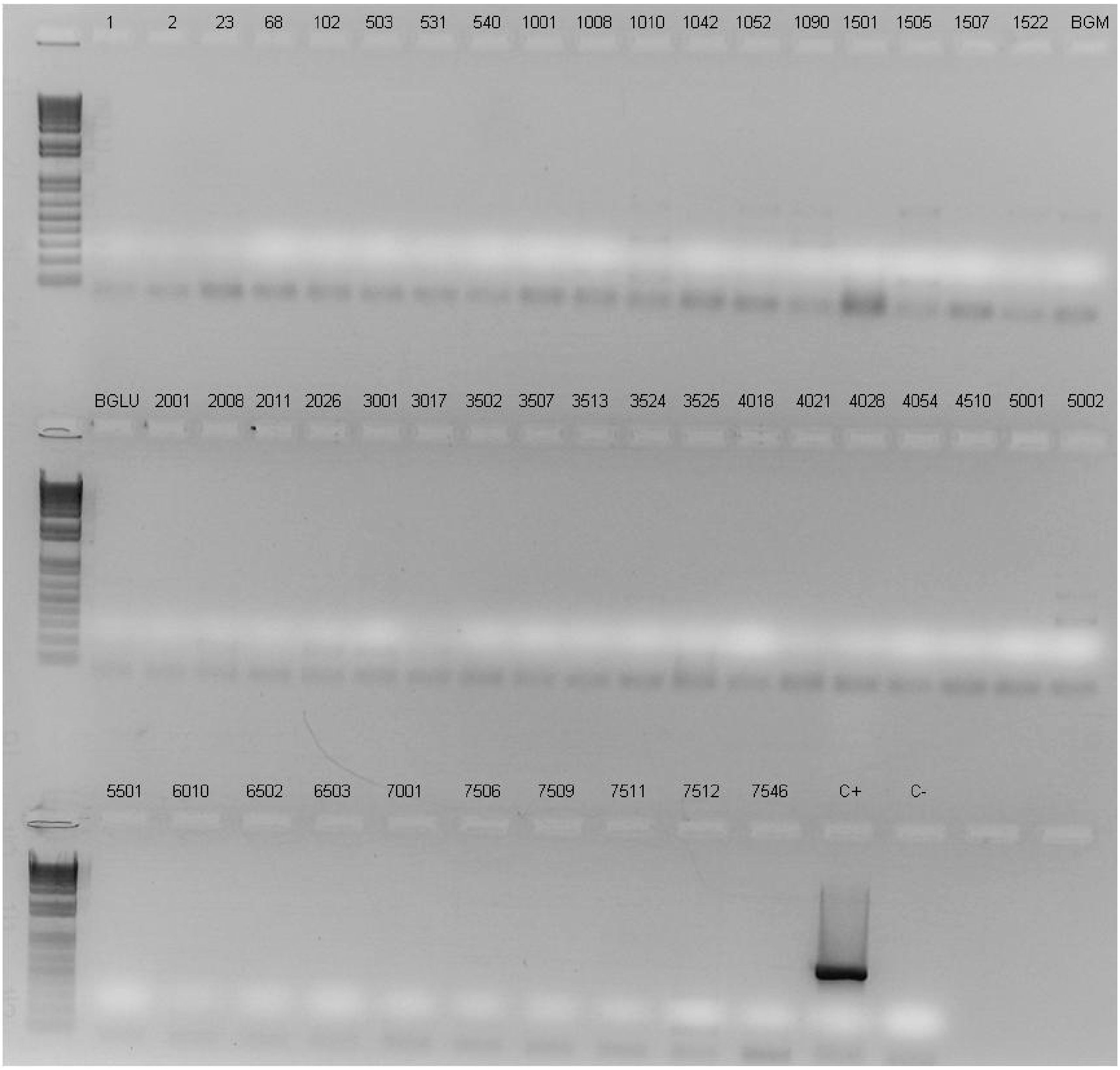

Figure S11: Agarose gel from the PCR amplification of the gene microcystin E GSA-AMT-domain for microcystin in 48 *Azolla* accessions (see table 3 from the manuscript). C+ -: positive control (*M. aeruginosa* LEGE 91094), C- : negative control, 1st line is Ladder

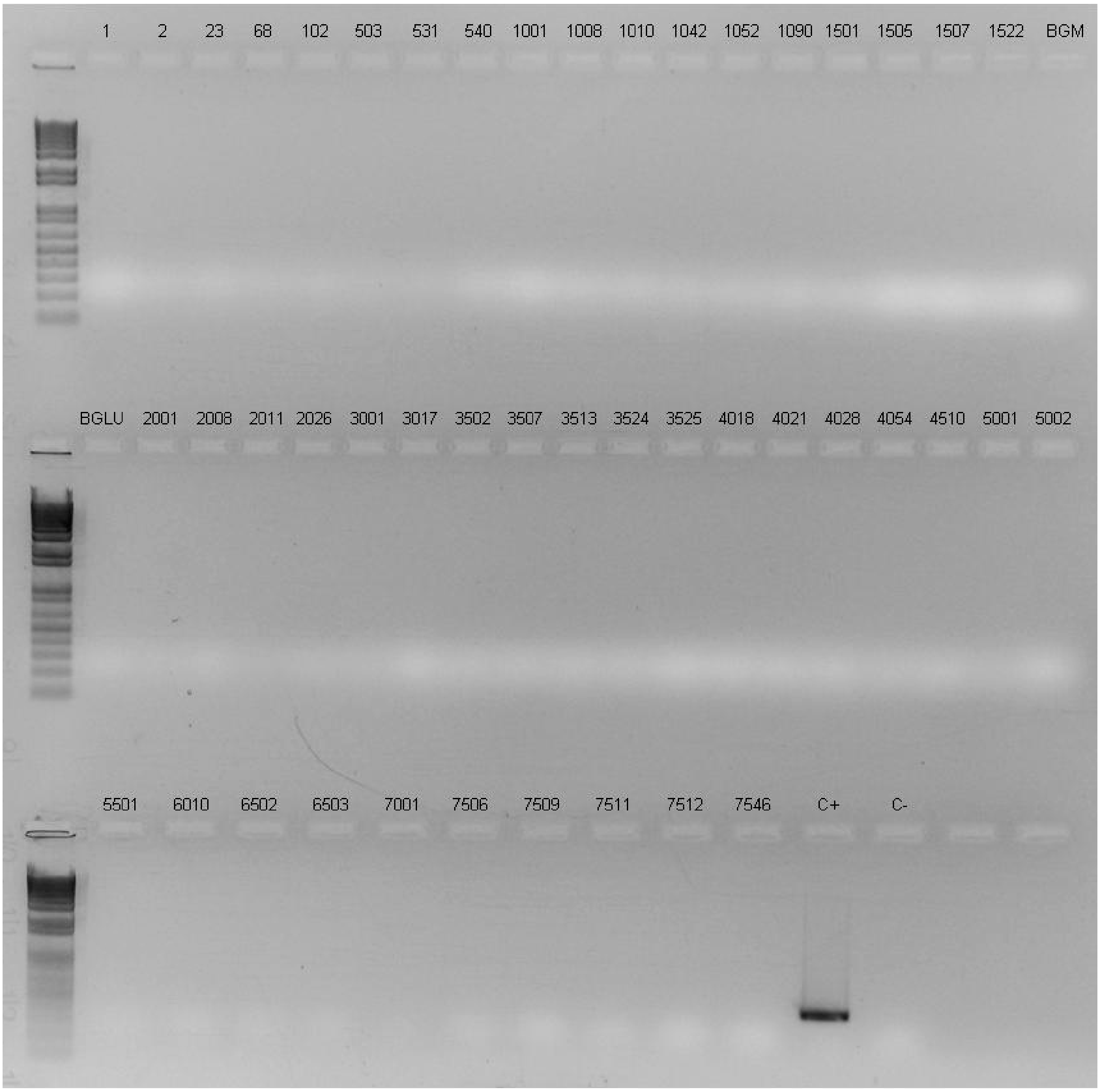

Figure S12: Agarose gel from the PCR amplification of the gene microcystin G CM-domain for microcystin in 48 *Azolla* accessions (see table 3 from the manuscript). C+ -: positive control (*M. aeruginosa* LEGE 91094), C-: negative control, 1st line is Ladder

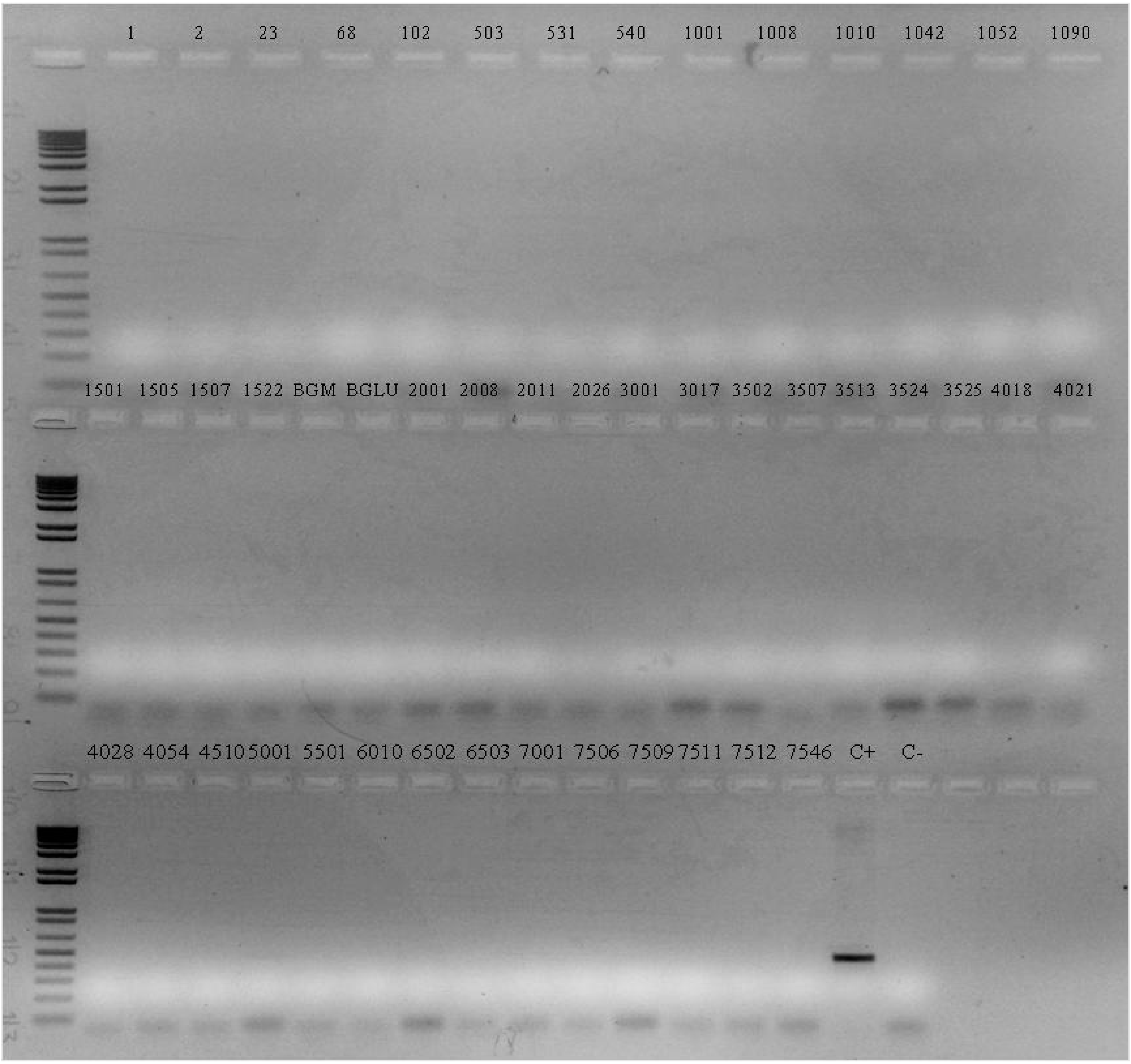

Figure S13: Agarose gel from the PCR amplification of the gene microcystin/nodularin synthetase for nodularin in 47 *Nostoc azollae* isolated from *Azolla* accessions (see table 3 from the manuscript). C+ -: positive control (*M. aeruginosa* LEGE 91094), C- : negative control, 1st line is Ladder

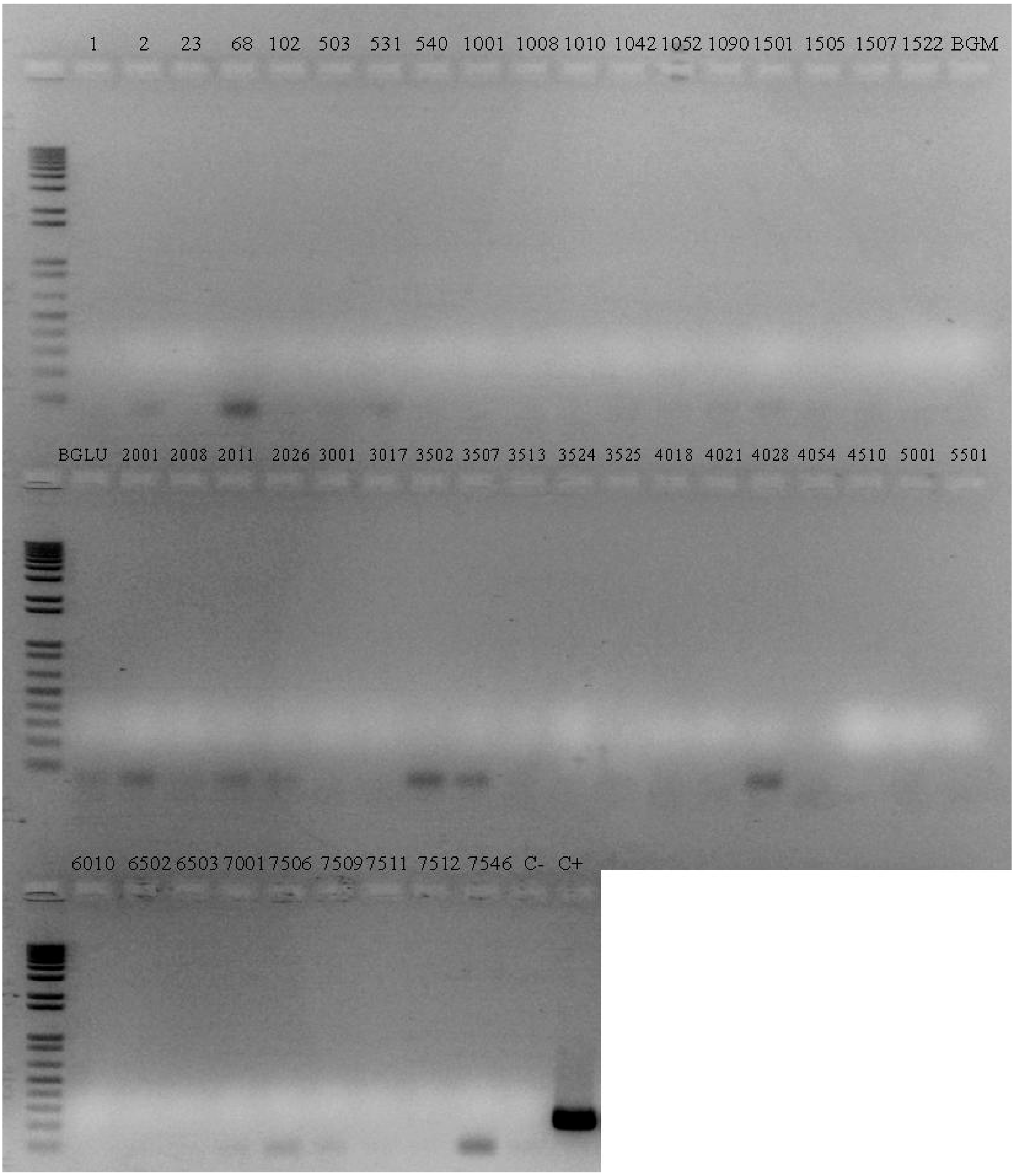

Figure S14: Agarose gel from the PCR amplification of the gene saxitoxin in 47 *Nostoc azollae* isolated from *Azolla* accessions (see table 3 from the manuscript). C+ -: positive control (*A. gracillaris* LMECYA 40), C- : negative control, 1st line is Ladder

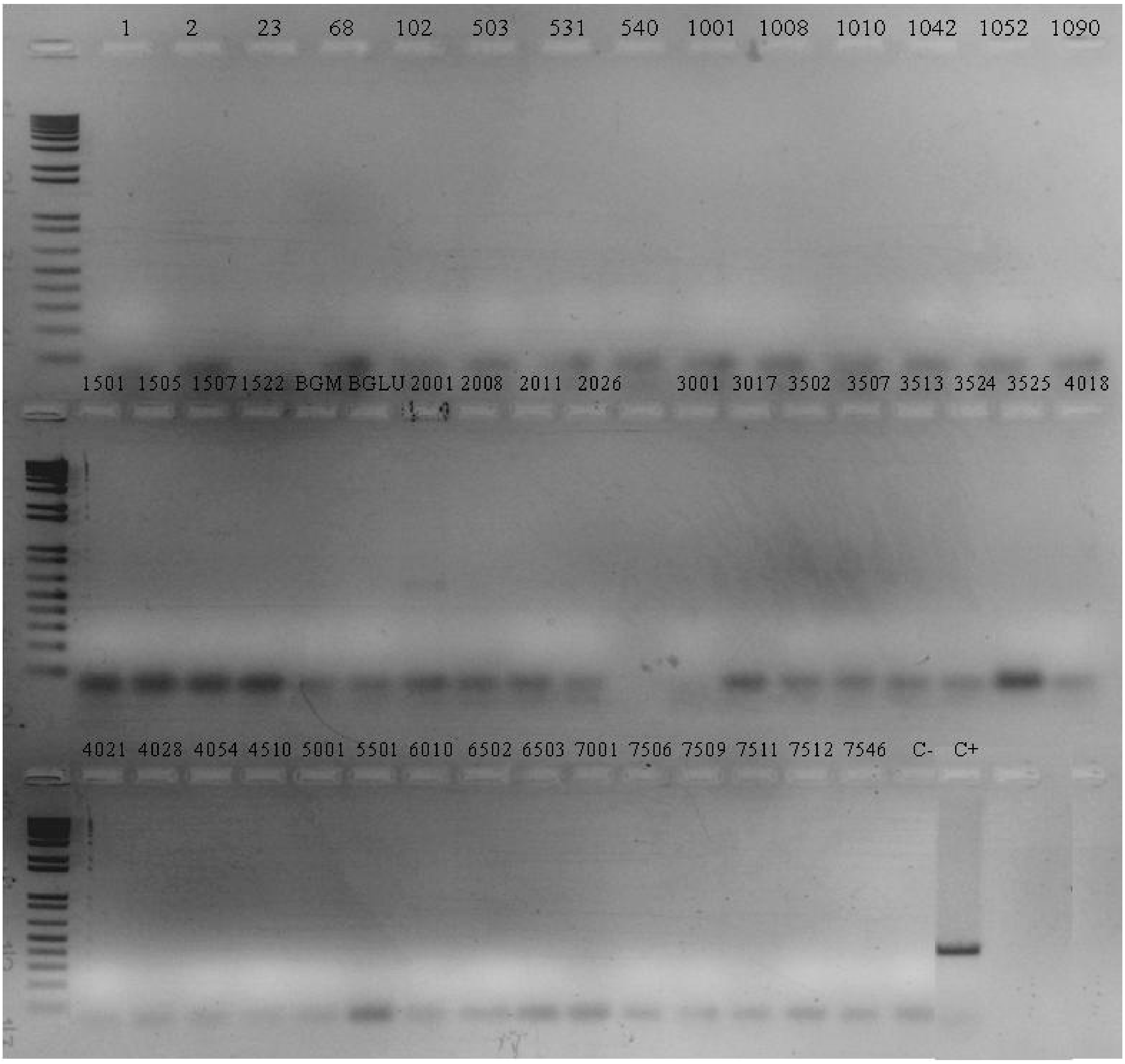

Figure S15: Agarose gel from the multiplex PCR amplification of the genes poliketide synthase and peptide synthase for cylindrospermopsin in 47 *Nostoc azollae* isolated from *Azolla* accessions (see table 3 from the manuscript). C+ -: positive control (*A. ovalisporum*), C- : negative control, 1st line is Ladder

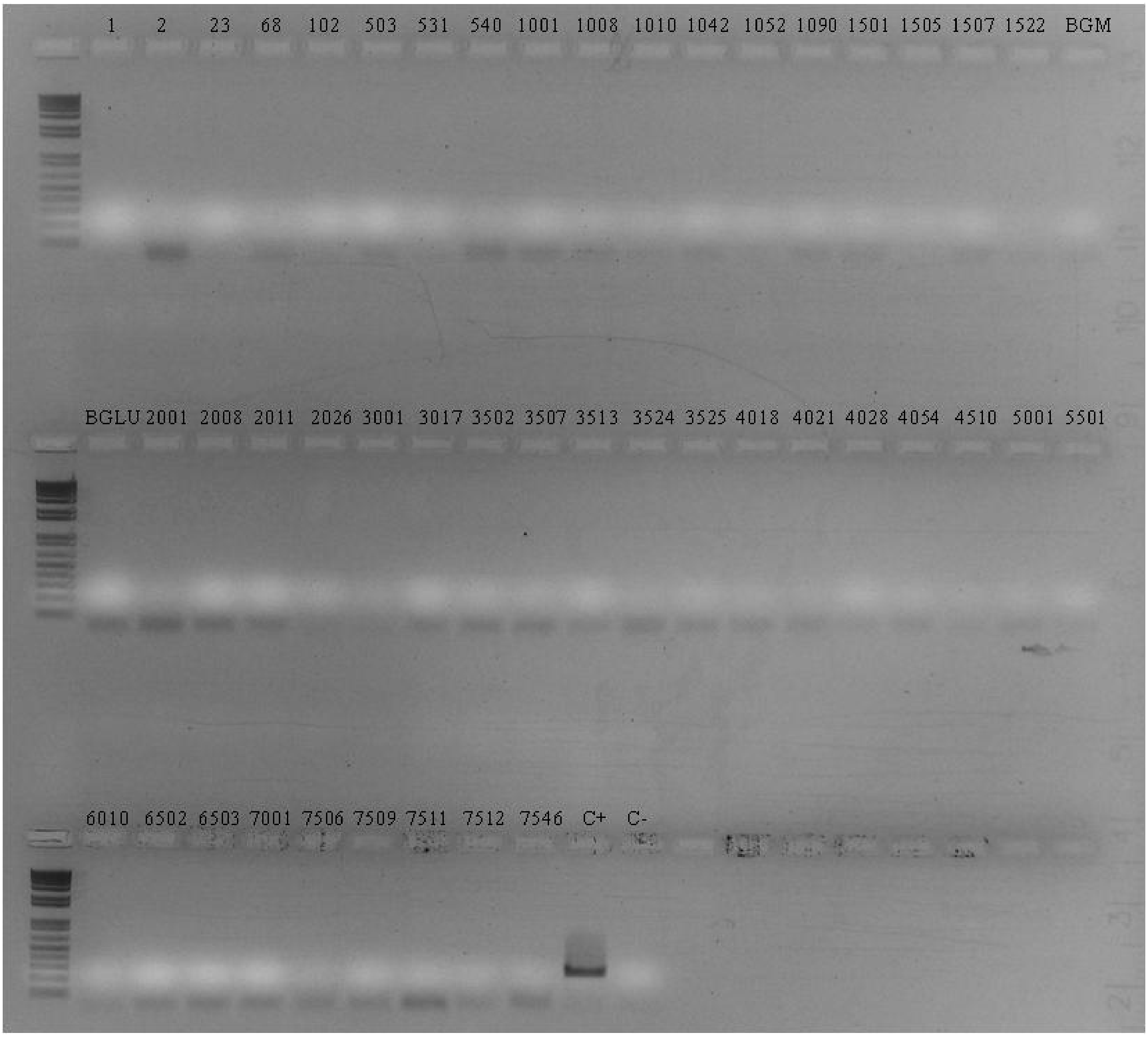

Figure S16: Agarose gel from the PCR amplification of the gene microcystin synthetase (mcy A) for microcystin in 47 *Nostoc azollae* isolated from *Azolla* accessions (see table 3 from the manuscript). C+ -: positive control (*M. aeruginosa* LEGE 91094), C- : negative control, 1st line is Ladder

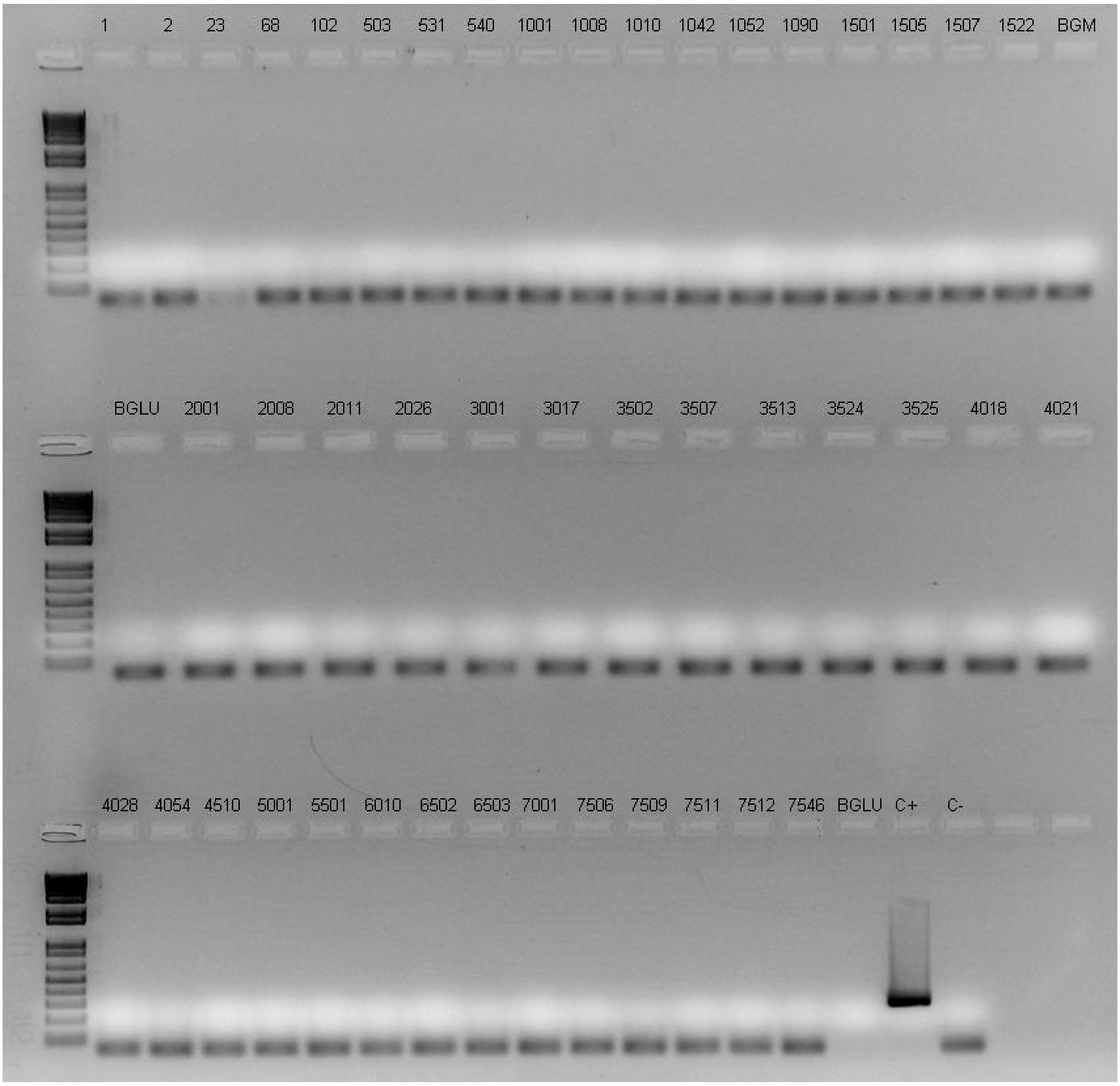

Figure S17: Agarose gel from the PCR amplification of the gene microcystin synthetase (mcy B) for microcystin in 47 *Nostoc azollae* isolated from *Azolla* accessions (see table 3 from the manuscript). C+ -: positive control (*M. aeruginosa* LEGE 91094), C- : negative control, 1st line is Ladder

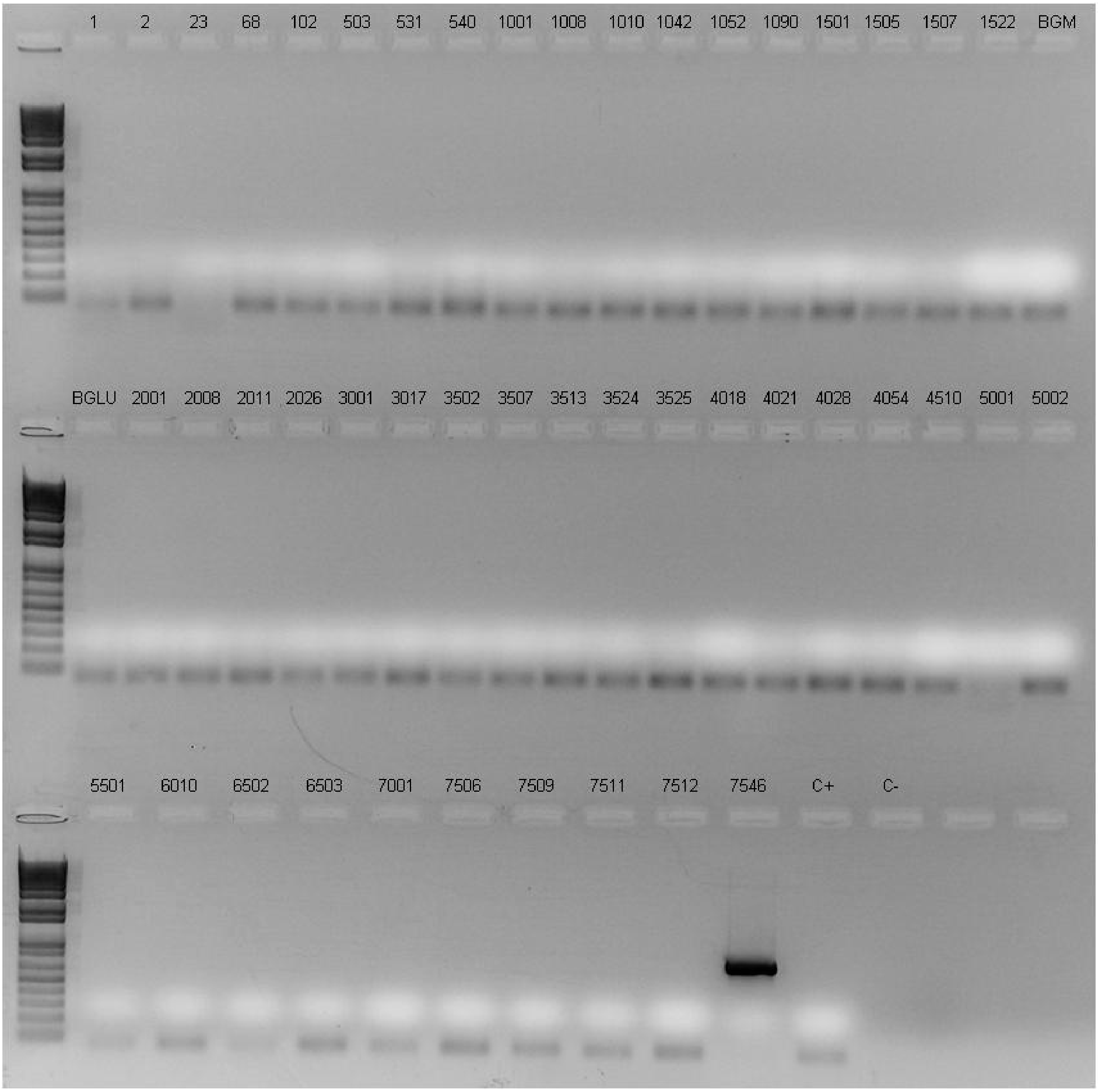

Figure S18: Agarose gel from the PCR amplification of the gene microcystin C for microcystin in 47 *Nostoc azollae* isolated from *Azolla* accessions (see table 3 from the manuscript). C+ -: positive control (*M. aeruginosa* LEGE 91094), C- : negative control, 1st line is Ladder

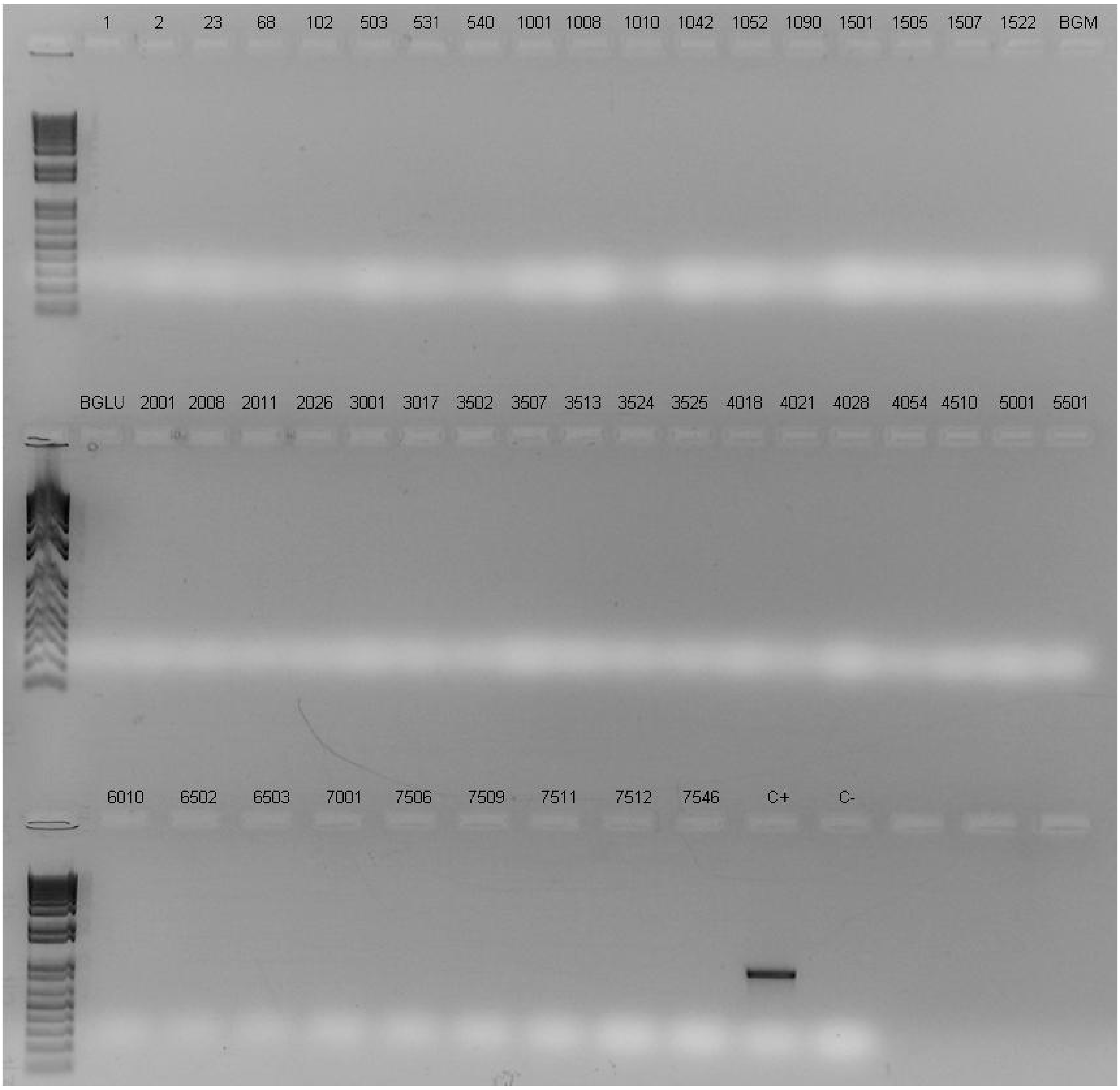

Figure S19: Agarose gel from the PCR amplification of the gene microcystin B A-domain for microcystin in 47 *Nostoc azollae* isolated from *Azolla* accessions (see table 3 from the manuscript). C+ -: positive control (*M. aeruginosa* LEGE 91094), C- : negative control, 1st line is Ladder

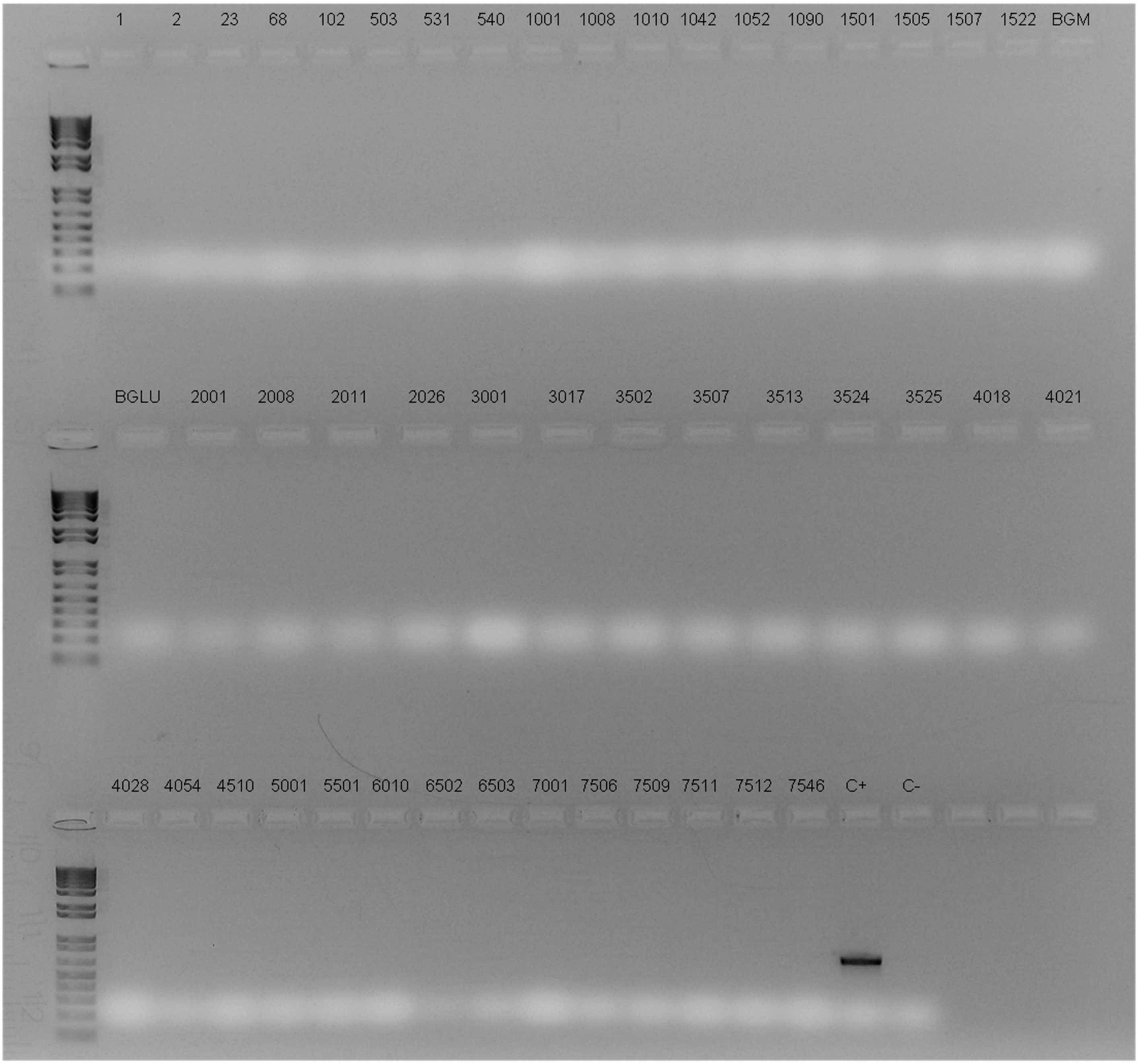

Figure S20: Agarose gel from the PCR amplification of the gene microcystin C A-domain for microcystin in 47 *Nostoc azollae* isolated from *Azolla* accessions (see table 3 from the manuscript). C+ -: positive control (*M. aeruginosa* LEGE 91094), C- : negative control, 1st line is Ladder

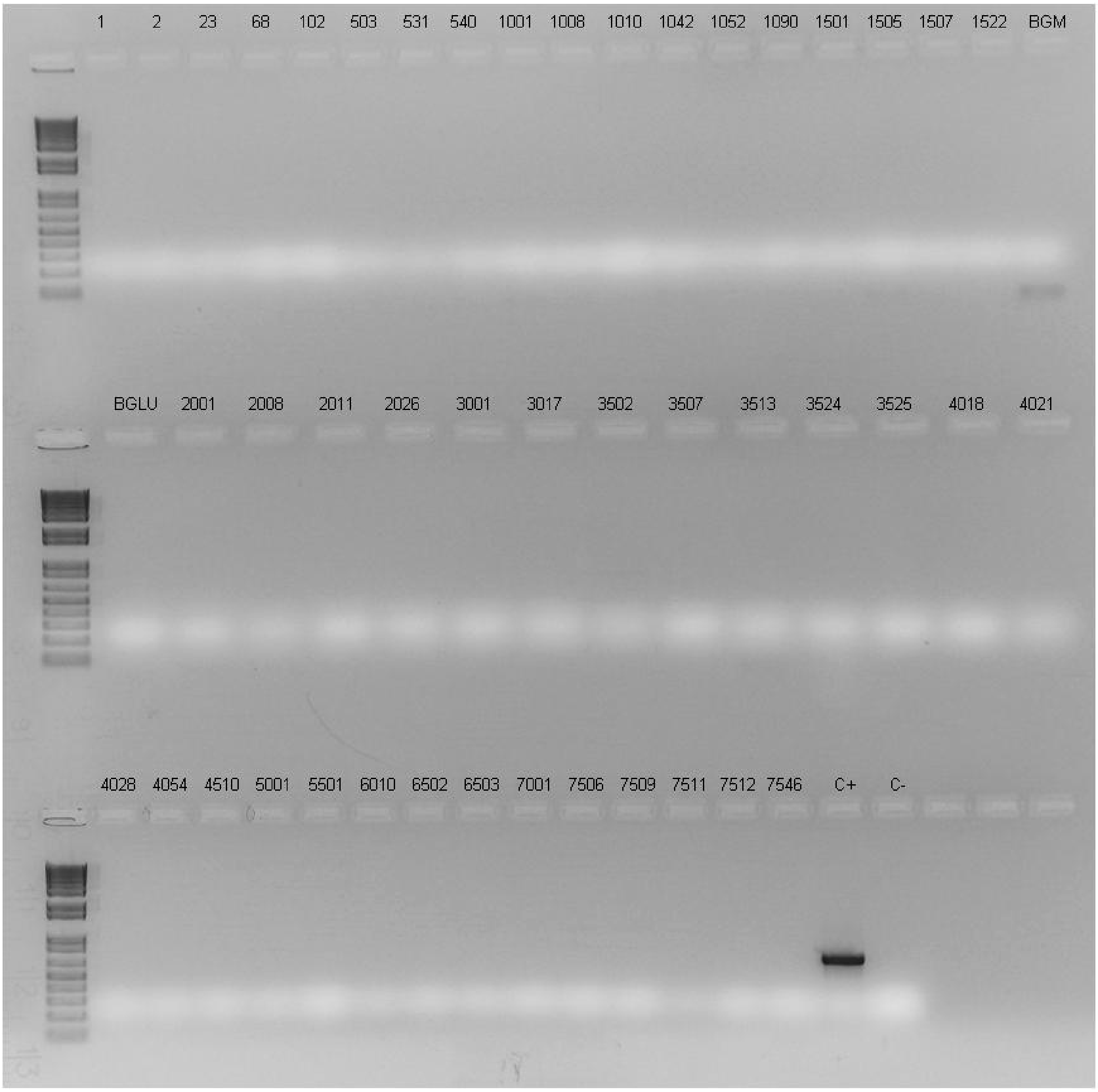

Figure S21: Agarose gel from the PCR amplification of the gene microcystin D ACP-domain for microcystin in 47 *Nostoc azollae* isolated from *Azolla* accessions (see table 3 from the manuscript). C+ -: positive control (*M. aeruginosa* LEGE 91094), C- : negative control, 1st line is Ladder

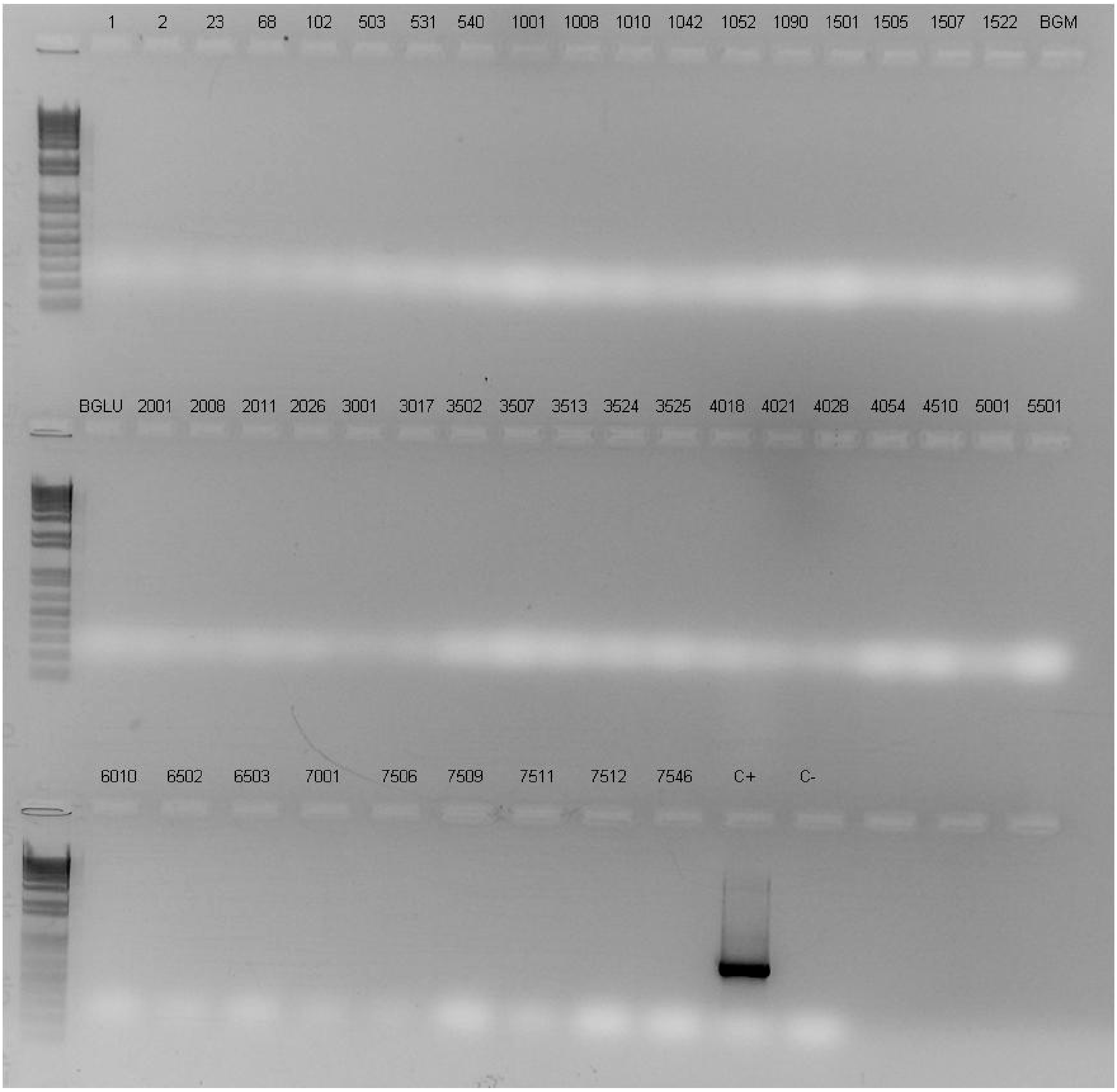

Figure S22: Agarose gel from the PCR amplification of the gene microcystin D KS-domain for microcystin in 47 *Nostoc azollae* isolated from *Azolla* accessions (see table 3 from the manuscript). C+ -: positive control (*M. aeruginosa* LEGE 91094), C- : negative control, 1st line is Ladder

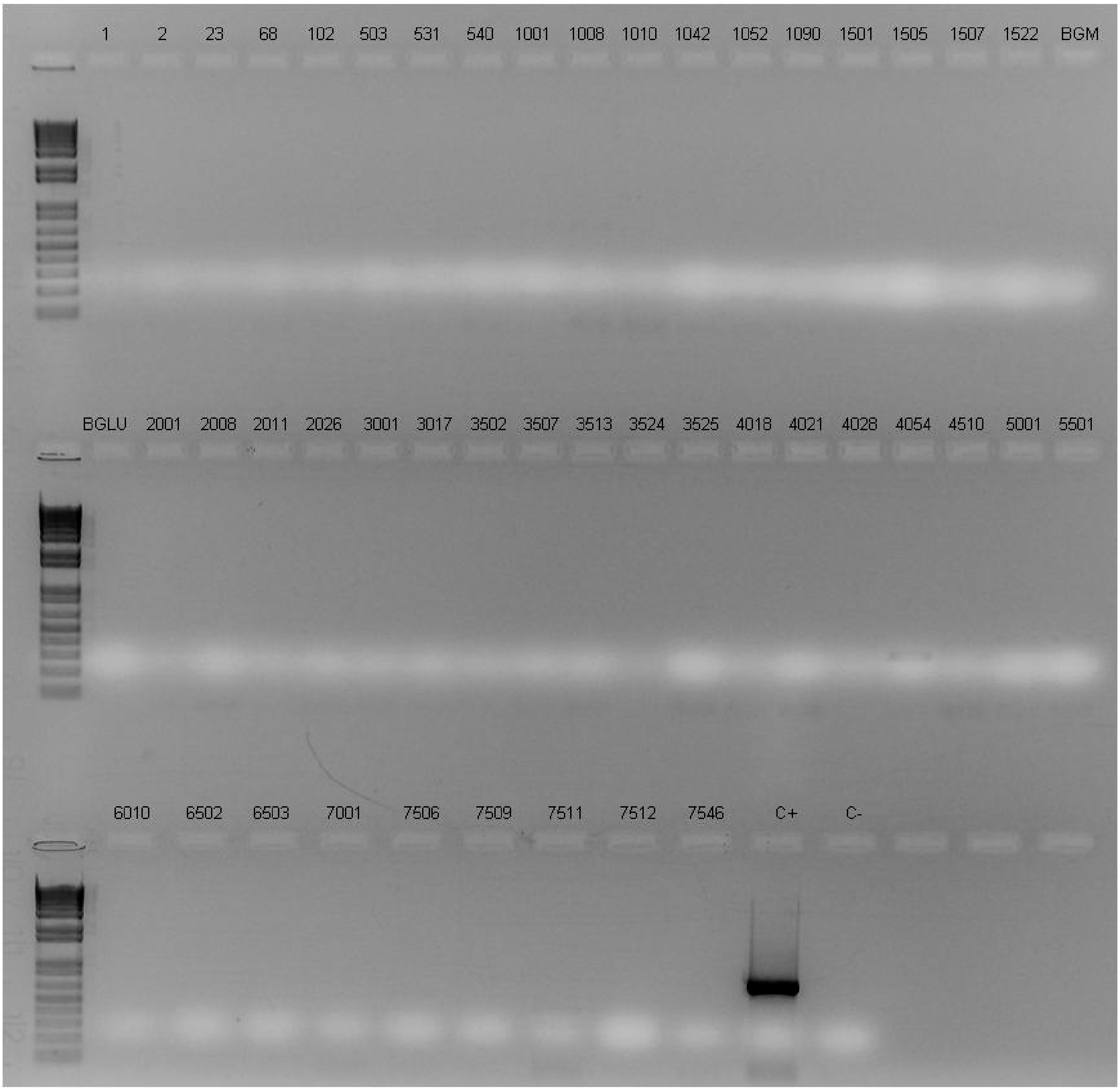

Figure S23: Agarose gel from the PCR amplification of the gene microcystin E GSA-AMT-domain for microcystin in 47 *Nostoc azollae* isolated from *Azolla* accessions (see table 3 from the manuscript). C+ -: positive control (*M. aeruginosa* LEGE 91094), C- : negative control, 1st line is Ladder

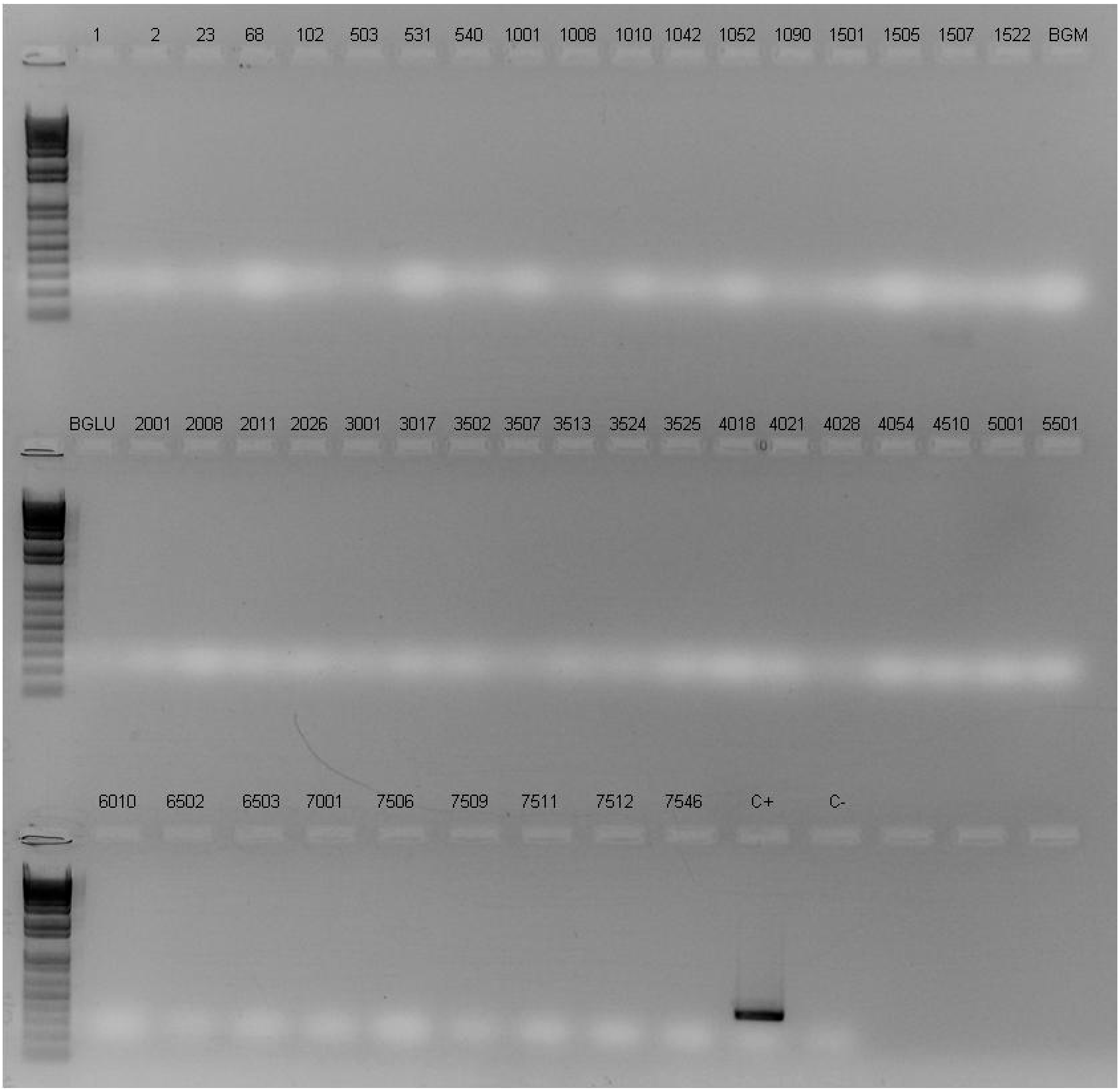

Figure S24: Agarose gel from the PCR amplification of the gene microcystin G CM-domain for microcystin in 47 *Nostoc azollae* isolated from *Azolla* accessions (see table 3 from the manuscript). C+ -: positive control (*M. aeruginosa* LEGE 91094), C- : negative control, 1st line is Ladder

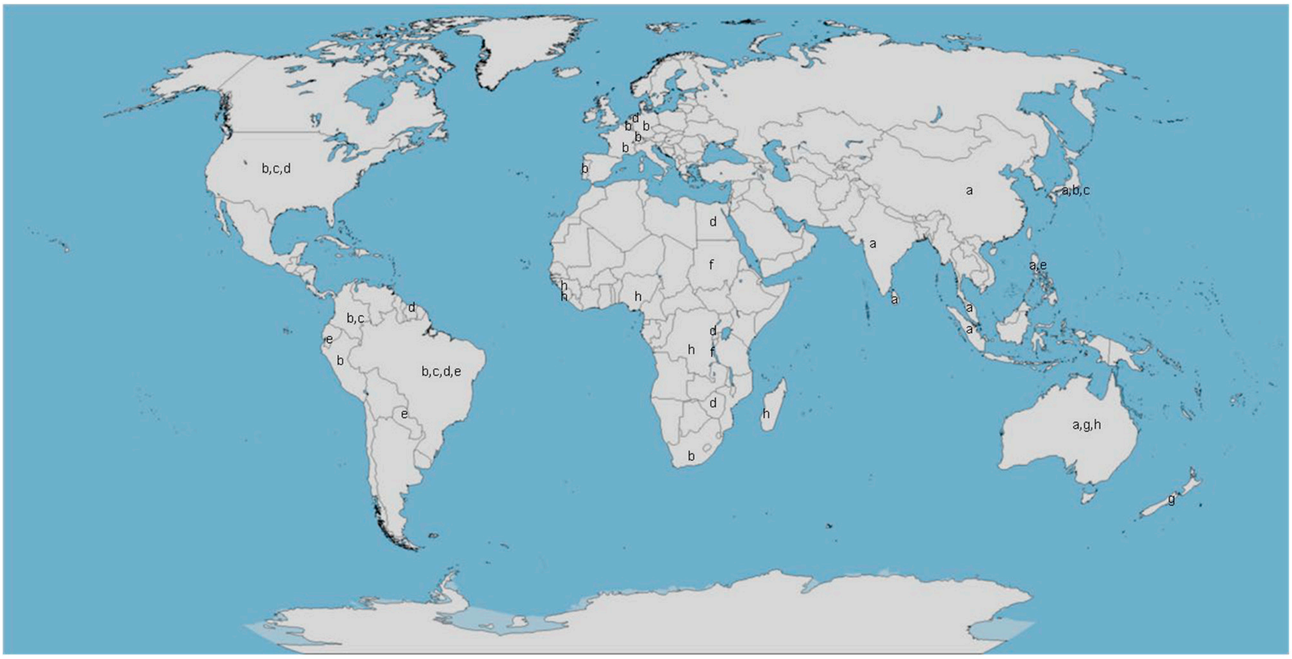

Figure S25: Worldwide map showing the countries of *Azolla* accessions origin used in the present study. Legend: a – *A. pinnata* subsp. *imbricata*; b - *A. filiculoides*; c – *A. mexicana*; d – *A. caroliniana*; e – *A. microphylla*; f - *A. nilotica*; g – *A. rubra*; h – *A. pinnata* subsp. *pinnata*

RID: 9JRTT34C013
Job Title:FJ477836:Oscillatoria sp. PCC 6506 anatoxin-a...
Program: BLASTN
Database: nt Nucleotide collection (nt)
Query #1: Oscillatoria sp. PCC 6506 anatoxin-a and homoanatoxin-a biosynthetic gene cluster, complete sequence Query ID: gb|FJ477836.2 Length: 34682

Sequences producing significant alignments:

```
                                                      Scientific      Common              Max    Total Query   E
Per.   Acc.
Description                                                Name            Name          Taxid     Score  Score cover
Value Ident  Len        Accession
'Nostoc azollae' 0708, complete genome                        'Nostoc azol... NA             551115     119
6465  3%     4e-25 73.04  5354700     CP002059.1
```

Alignments:

>'Nostoc azollae' 0708, complete genome
Sequence ID: CP002059.1 Length: 5354700
Range 1: 4676901 to 4677127

Score:119 bits(131), Expect:4e-25,
Identities:168/230(73%), Gaps:6/230(2%), Strand: Plus/Minus

```
Query  34237    GATTTATATTAGCCACAAATATCTTAGATACTGAAGCAGTAAGCAATCCACAGGTATTAT  34296
                ||||||| ||||| |||||||| ||||| ||  | | | | || || ||| ||  || ||
Sbjct  4677127  GATTTATTTTAGCAACAAATATTTTAGAGACA-AGGGAATTAGAAA-CCATAGAAAT-AT  4677071

Query  34297    TG-GAATATAAAGCG--CAGCAAAGTAATGAGAGAGGATTTAGATTTATCAAAGATCCGT  34353
                || ||||||| || |  ||||||   |  || ||||||||||||||||||||||| ||||
Sbjct  4677070  TGAGAATATATAGAGAACAGCAATCAACAGAAAGAGGATTTAGATTTATCAAAGACCCGT  4677011

Query  34354    TATTTTTTACTTCCAGTGTATTCGTGAAAAAGCCGGAGCGAGTTGAAGCAATCGGAATGA  34413
                ||||||| ||    ||| | || ||||| || || ||  |||| ||  ||||    |||
Sbjct  4677010  TATTTTTCACCGATAGTCTTTTTGTGAAGAATCCAGAAAGAGTAGAGACAATGATGATGT  4676951

Query  34414    TAATGGGACTGTGTTTGTTAGTATATAACCTGGCGCAAAGGAAATTGAGA  34463
                |||||| | ||||| | || || ||||| ||||  ||||   |||| |||
Sbjct  4676950  TAATGGCATTGTGTCTTTTGGTTTATAATCTGGGACAAAAACAATTAAGA  4676901
```

Range 2: 5107612 to 5107834

Score:106 bits(117), Expect:3e-21,
Identities:164/227(72%), Gaps:8/227(3%), Strand: Plus/Minus

```
Query  34237    GATTTATATTAGCCACAAATATCTTAGATACTGAAGC-AGTAAGCAATCCACAGGTATTA  34295
                ||||||| ||||| || ||||| ||||| ||  |||| | | || || ||| ||  || |
Sbjct  5107834  GATTTATTTTAGCAACGAATATTTTAGAGAC--AAGCGAATTAGAAA-CCATAGAAAT-A  5107779

Query  34296    TTG-GAATATA--AAGCGCAGCAAAGTAATGAGAGAGGATTTAGATTTATCAAAGATCCG   34352
                ||| |||||||  |||  | | ||   |  || ||||||||||||||||| ||||| |||
Sbjct  5107778  TTGAGAATATATAAAGAACCGTAATCAACAGAAAGAGGATTTAGATTTATTAAAGACCCG  5107719
```

file:///C/...SUBMISSION%20AUGUST%2021%202024/SUPPLEMENTARY%20MATERIAL/BLASTN_Alignments_anatoxina_Nazollae.txt[22/08/2024 13:13:51]

```
Query  34353    TTATTTTTTACTTCCAGTGTATTCGTGAAAAAGCCGGAGCGAGTTGAAGCAATCGGAATG  34412
                ||||||   |    ||| | || ||||||| || ||  |||| ||  ||||     |||
Sbjct  5107718  TTATTTTCCGCCCATAGTCTTTTTGTGAAAAATCCAGAAAGAGTAGAGACAATGATGATG  5107659

Query  34413    ATAATGGGACTGTGTTTGTTAGTATATAACCTGGCGCAAAGGAAATT  34459
                 |||||| | ||||||| || || ||||| ||||  |||||  ||||
Sbjct  5107658  TTAATGGCATTGTGTTTTTTGGTTTATAATCTGGGACAAAGACAATT  5107612
```

Range 3: 3669515 to 3669746

Score:105 bits(116), Expect:3e-21,
Identities:169/235(72%), Gaps:6/235(2%), Strand: Plus/Minus

```
Query  34232    TGGGAGATTTATATTAGCCACAAATATCTTAGATACTGAAGCAGTAAGCAATCCACAGGT  34291
                ||| ||||||||  |||| || ||||| ||||| ||  | | | | || || ||| ||
Sbjct  3669746  TGGTAGATTTATTCTAGCAACGAATATTTTAGAGACA-AGGGAATTAGAAA-CCATAGAA  3669689

Query  34292    ATTATTG-GAATATAAAGCGCAG-CAAAGTAAT-GAGAGAGGATTTAGATTTATCAAAGA  34348
                || |||| ||||||| || | || ||||  ||  || ||||||||||||||||||||||
Sbjct  3669688  AT-ATTGAGAATATATAGAGAAGACAAATCAACAGAAAGAGGATTTAGATTTATCAAAGA  3669630

Query  34349    TCCGTTATTTTTTACTTCCAGTGTATTCGTGAAAAAGCCGGAGCGAGTTGAAGCAATCGG  34408
                 ||||||||||  |    ||| | || ||||| || || ||  || | | ||  ||||
Sbjct  3669629  CCCGTTATTTTTCGCCCATAGTCTTTTTGTGAAGAATCCAGAAAGACTAGAGACAATGAT  3669570

Query  34409    AATGATAATGGGACTGTGTTTGTTAGTATATAACCTGGCGCAAAGGAAATTGAGA  34463
                 ||| |||||| | ||||| | || ||  |||| ||||  |||||  |||| |||
Sbjct  3669569  GATGTTAATGGCATTGTGTCTTTTGGTTCATAATCTGGGACAAAGACAATTAAGA  3669515
```

Range 4: 2358560 to 2358789

Score:102 bits(112), Expect:3e-20,
Identities:170/236(72%), Gaps:15/236(6%), Strand: Plus/Plus

```
Query  34237    GATTTATATTAGCCACAAATATCTTAGATACTGAAGCAG-TAAGCAATCCACAGGTATTA  34295
                ||||||| ||||| | |||||| |||||  |  |||||  | || || ||| ||  || |
Sbjct  2358560  GATTTATTTTAGCAAAAAAATATTTTAGAGCC--AAGCAAATTAGAAA-CCATAGAAAT-A  2358615

Query  34296    TTG-GAATATA--AAGCGCAGCAAAGTAATGAGAGAGGATTTAGATTTATCAAAGATCCG  34352
                ||| |||||||  |||  ||||||    |  || |||||| |||||||||||||||| | |
Sbjct  2358616  TTGAGAATATATAAAGAACAGCAATCAACAGAAAGAGGACTTAGATTTATCAAAGACCTG  2358675

Query  34353    TTATTTTTTACTTCCAGTGTATTC--GTGAAAAAGCCGGAGCGAGTTGAAG---CAATCG  34407
                |||||||| ||  |||  || |||  || ||||| || ||  |||| | ||    ||||
Sbjct  2358676  TTATTTTTCAC--CCATAGTCTTCTTGTAAAAAATCCAGAAAGAGTAGTAGAGACAATAA  2358733

Query  34408    GAATGATAATGGGACTGTGTTTGTTAGTATATAACCTGGCGCAAAGGAAATTGAGA  34463
                  ||| |||||| | ||||| | || || ||||| ||||  |||||| || | |||
Sbjct  2358734  TGATGTTAATGGCATTGTGTCTTTTGGTTTATAATCTGGGACAAAGGCAACTAAGA  2358789
```

Range 5: 73160 to 73386

Score:100 bits(110), Expect:1e-19,
Identities:165/231(71%), Gaps:8/231(3%), Strand: Plus/Minus

```
Query  34237  GATTTATATTAGCCACAAATATCTTAGATACTGAAGCAG-TAAGCAATCCACAGGTATTA  34295
              ||||||| ||||| |   |||| ||||| ||  ||| || | || |||| | ||| || |
Sbjct  73386  GATTTATTTTAGCAAGGCATATTTTAGAGAC--AAGGAGATTAGAAATC-ATAGGAAT-A  73331

Query  34296  TTG-GAATATAAAGCGCAGCAAAGTAATGAGA--GAGGATTTAGATTTATCAAAGATCCG  34352
              ||| ||||||| || | ||  || | |  |||  |||||||||||||||||| || |||
Sbjct  73330  TTGAGAATATATAGAGAAGAGAAATCAACAGAAAGAGGATTTAGATTTATCAAGGACCCG  73271

Query  34353  TTATTTTTACTTCCAGTGTATTCGTGAAAAAGCCGGAGCGAGTTGAAGCAATCGGAATG  34412
              |||||||| |    ||| | || |||||||  || ||  |||| ||  ||||    |||
Sbjct  73270  TTATTTTTCGCCCATAGTCTTTTTGTGAAAAGTCCAGAAAGAGTAGAGACAATGATGATG  73211

Query  34413  ATAATGGGACTGTGTTTGTTAGTATATAACCTGGCGCAAAGGAAATTGAGA  34463
              |||||| | || || | || || ||||| ||||   ||||  ||||||||
Sbjct  73210  TTAATGGCATTGGGTCTTTTGGTTTATAATCTGGGAGAAAGACAATTGAGA  73160
```

Range 6: 4284799 to 4285025

Score:99.6 bits(109), Expect:4e-19,
Identities:166/232(72%), Gaps:10/232(4%), Strand: Plus/Minus

```
Query  34237    GATTTATATTAGCCACAAATATCTTAGATACTGAAGCAGTAAGCAATCCACAGGTATTAT  34296
                ||||||| ||||| || ||||| ||||| ||  | | | | || || ||| ||  || ||
Sbjct  4285025  GATTTATTTTAGCAACGAATATTTTAGACACA-AGGGAATTAGGAA-CCATAGAAAT-AT  4284969

Query  34297    TG-GAATATAAAGCGCA--GCAAAGTAATGAGAGAGGATTTAGATTTATCAAAGATCCGT  34353
                || ||||||| || | |  ||||   |  || |||||||||| |||||||||||| ||||
Sbjct  4284968  TGAGAATATATAGAGAATAGCAATCAACAGAAAGAGGATTTATATTTATCAAAGACCCGT  4284909

Query  34354    TATTTTTACTTCC--AGTGTATTCGTGAAAAAGCCGGAGCGAGTTGAAGCAATCGGAAT  34411
                |||||||  |  ||  ||| | || |||||| |||  ||  |||| ||  ||||    ||
Sbjct  4284908  TATTTTT--CGACCATAGTCTTTTTGTGAAAGAGCGAGAAAGAGTAGAGACAATGATGAT  4284851

Query  34412    GATAATGGGACTGTGTTTGTTAGTATATAACCTGGCGCAAAGGAAATTGAGA  34463
                 |||||| | ||||| | || || ||||| ||||  |||||  |||| |||
Sbjct  4284850  ATTAATGGCATTGTGTCTTTTGGTTTATAATCTGGGACAAAGACAATTAAGA  4284799
```

Range 7: 3925786 to 3926001

Score:98.7 bits(108), Expect:4e-19,
Identities:158/220(72%), Gaps:8/220(3%), Strand: Plus/Plus

```
Query  34237    GATTTATATTAGCCACAAATATCTTAGATACTGAAGC-AGTAAGCAATCCACAGGTATTA  34295
                ||||||| ||||| || ||||| ||||| ||  |||| | | |  || ||| ||  || |
```

Sbjct 3925786 GATTTATTTTAGCAACGAATATTTTAGAGAC--AAGCGAATTATAAA-CCATAGAAAT-A 3925841

Query 34296 TTG-GAATATA--AAGCGCAGCAAAGTAATGAGAGAGGATTTAGATTTATCAAAGATCCG 34352
||| ||||||| ||| || | | | || |||||||||||||||||||||| |||
Sbjct 3925842 TTGAGAATATATAAAGAACACCTATCAACAGAAAGAGGATTTAGATTTATCAAAGACCCG 3925901

Query 34353 TTATTTTTTACTTCCAGTGTATTCGTGAAAAAGCCGGAGCGAGTTGAAGCAATCGGAATG 34412
| |||||| | ||| | || |||||||| || || |||| || |||| |||
Sbjct 3925902 TCATTTTTCGCCCATAGTCTTTTTGTGAAAAATCCAGAAAGAGTAGAGACAATGATGATG 3925961

Query 34413 ATAATGGGACTGTGTTTGTTAGTATATAACCTGGCGCAAA 34452
|||||| | ||||| | || || || ||||||| ||||
Sbjct 3925962 TTAATGGCATTGTGTCTTTTGGTTTACAACCTGGGACAAA 3926001

Range 8: 234664 to 234880

Score:94.2 bits(103), Expect:2e-17,
Identities:151/217(70%), Gaps:4/217(1%), Strand: Plus/Minus

Query 6897 CAATCCCACCAATAA----CGCTAAATATTGGCTTGTGGCATAAGAAACTCCTGCCCCTA 6952
||||| |||||||| ||| ||||| |||||| || || | ||||| ||
Sbjct 234880 CAATCAAACCAATAATTAGCGCCAAATACTGGCTTAAAGCTGTAGCTAAACCTGCTCCAG 234821

Query 6953 CACTTTCCCATCCCAGGTAGATAATAAGTAGATAGTCAAGTGCGATTTTGCCACCATTTC 7012
|||||||||| ||| | || ||| | || || || || | || |||||| |
Sbjct 234820 CACTTTCCCAACCCCACTTGACAATCATTAAGTAATCCAGCAACACATTAGAACCATTAC 234761

Query 7013 CAAGGATTGACAAAAACACAACTAAGCCAtttttttCTCGCCCTAGAAACCAACCTATCA 7072
||| ||||||| | || |||| ||||||||||| | ||||||||||||| | |
Sbjct 234760 CAACAATTGACATCAGCAGCACTACACCATTTTTTTCCCTTCCTAGAAACCAACCAAATA 234701

Query 7073 GGACAAAATTGAGTAAAATTGCAGGCGCTCCCCAAAT 7109
||||| || ||||||| ||||| || ||||||||
Sbjct 234700 ACACAAAGTTCAGTAAAACAGCAGGTGCACCCCAAAT 234664

Range 9: 2101937 to 2102146

Score:88.7 bits(97), Expect:7e-16,
Identities:150/213(70%), Gaps:6/213(2%), Strand: Plus/Plus

Query 34230 TCTGGGAGATTTATATTAGCCACAAATATCTTAGATACTGAAGCAGTAAGCAATCCACAG 34289
||||||||||||| ||||| || ||||| ||||||| ||| ||| || ||||| ||
Sbjct 2101937 TCTGGGAGATTTATTTTAGCAACTAATATTTTAGATA-TGACAGAGTTAG-AATCCTGAG 2101994

Query 34290 GTATTATTGGAA---TATAAAGCGCAGCAAAGTAATGAGAGAGGATTTAGATTTATCAAA 34346
| |||| || |||||| ||||||| | |||||||||||||||||||
Sbjct 2101995 A-ACTATTTAAAATCTATAAACAGCAGCAATCCTCTTTCTGAGGATTTAGATTTATCAAA 2102053

Query 34347 GATCCGTTATTTTTTACTTCCAGTGTATTCGTGAAAAAGCCGGAGCGAGTTGAAGCAATC 34406
|| || ||||||| | | ||| | | |||||||| || || |||| || ||||
Sbjct 2102054 GACCCTTTATTTTCTGCAGATAGTCTTCTTGTGAAAAATCCCGAAGGAGTAGACACAATT 2102113

```
Query  34407    GGAATGATAATGGGACTGTGTTTGTTAGTATAT  34439
                   ||  ||  || | ||||| | ||||| |||
Sbjct  2102114  ATGATTTTATCGGCATTGTGTCTTTTAGTTTAT  2102146
```

Range 10: 2973703 to 2973929

Score:86.9 bits(95), Expect:2e-15,
Identities:162/231(70%), Gaps:8/231(3%), Strand: Plus/Plus

```
Query  34237    GATTTATATTAGCCACAAATATCTTAGATACTGAAGC-AGTAAGCAATCCACAGGTATTA  34295
                ||||||  ||||| || ||||| ||||| |  |||| | | |  || ||| ||  || |
Sbjct  2973703  GATTTACTTTAGCAACGAATATTTTAGAGAC--AAGCGAATTATAAA-CCATAGAAAT-A  2973758

Query  34296    TTG-GAATATAAAGCGCAGCAAAGTAATGAGA--GAGGATTTAGATTTATCAAAGATCCG  34352
                ||| ||||||| || | ||  || | | ||||  |||||||||||||||||||| |||
Sbjct  2973759  TTGAGAATATATAGAGAAGAGAAATCAAGAGAAAGAGGATTTAGATTTATCAAAGACCCG
2973818

Query  34353    TTATTTTTTACTTCCAGTGTATTCGTGAAAAAGCCGGAGCGAGTTGAAGCAATCGGAATG  34412
                ||||||||  |    ||| | || ||  ||||     |  |||| ||  ||||    |||
Sbjct  2973819  TTATTTTTCGCCCATAGTCTTTTTGTAGAAAATATATAAAGAGTAGAGACAATGATGATG  2973878

Query  34413    ATAATGGGACTGTGTTTGTTAGTATATAACCTGGCGCAAAGGAAATTGAGA  34463
                 |||||| | ||||| | ||  | ||| | ||||  ||||| ||||| |||
Sbjct  2973879  TTAATGGCATTGTGTCTTTTGCTTTATGATCTGGGACAAAGAAAATTAAGA  2973929
```

Range 11: 4252048 to 4252274

Score:86.9 bits(95), Expect:2e-15,
Identities:161/231(70%), Gaps:8/231(3%), Strand: Plus/Minus

```
Query  34237    GATTTATATTAGCCACAAATATCTTAGATACTGAAGCAGTAAGCAATCCACAGGTATTAT  34296
                ||||||| ||||| |||||||| ||||| ||  | | | | || || |||  || ||  |
Sbjct  4252274  GATTTATTTTAGCAACAAATATTTTAGAGACA-AGGGAATTAGAAA-CCATGGGAATATT  4252217

Query  34297    TGGAATATAAAGCG--CAGCAAAGTAATGAGAGAGGATTTAGATTTATCAAAGATCCGTT  34354
                |  ||||||  || |  ||||||   |  || ||||||||||||||||||||||| |||||
Sbjct  4252216  TAAAATATATAGAGAACAGCAATCAACAGAAAGAGGATTTAGATTTATCAAAGACCCGTT  4252157

Query  34355    ATTTTTTACTTCCAGTGTATTCGTGAAAAAGCCGGAGCGAGTTGAAGCAAT--CGGAATG  34412
                ||||||  |    | | |  | || ||| |||  ||   ||| ||  ||||   || ||
Sbjct  4252156  ATTTTTCGCCCATACTCTTGTTGTAAAAGAGCGAGAAATAGTAGAGACAATGATGGTAT-  4252098

Query  34413    ATAATGGGACTGTGTTTGTTAGTATATAACCTGGCGCAAAGGAAATTGAGA  34463
                 |||||| | ||||| | || || ||||| ||||  ||||   |||| |||
Sbjct  4252097  -TAATGGCATTGTGTCTTTTGGTTTATAATCTGGGACAAAAACAATTAAGA  4252048
```

Range 12: 906195 to 906421

Score:82.4 bits(90), Expect:3e-14,
Identities:161/231(70%), Gaps:8/231(3%), Strand: Plus/Plus

```
Query  34237   GATTTATATTAGCCACAAATATCTTAGATACTGAAGC-AGTAAGCAATCCACAGGTATTA  34295
               |||||||  |||| || |||||  ||||  |  |||  | | || || ||| ||| || |
Sbjct  906195  GATTTATTCTAGCAACGAATATTCTAGAGGC--AAGTGAATTAGAAA-CCATAGGAAT-A  906250

Query  34296   TTG-GAATATAAAGCG--CAGCAAAGTAATGAGAGAGGATTTAGATTTATCAAAGATCCG   34352
               ||| ||||||| || |  ||||||   |  ||  ||| ||||||||||||||||| |||
Sbjct  906251  TTGAGAATATATAGAGAACAGCAATCAACAGAAGGAGTATTTAGATTTATCAAAGACCCG  906310

Query  34353   TTATTTTTTACTTCCAGTGTATTCGTGAAAAAGCCGGAGCGAGTTGAAGCAATCGGAATG   34412
               ||||||||  |    ||| | || ||||| ||  | ||  |||| ||    |||    |||
Sbjct  906311  TTATTTTTCGCCCATAGTCTTTTTGTGAAGAATTCAGAAAGAGTAGAGATAATGATAATT  906370

Query  34413   ATAATGGGACTGTGTTTGTTAGTATATAACCTGGCGCAAAGGAAATTGAGA  34463
               |||||| | ||||| | || || ||||| ||||    ||||  |||| |||
Sbjct  906371  TTAATGGCATTGTGTCTTTTGGTTTATAATCTGGGAGAAAGACAATTAAGA  906421
```

Range 13: 2601272 to 2601536

Score:78.8 bits(86), Expect:4e-13,
Identities:181/270(67%), Gaps:6/270(2%), Strand: Plus/Plus

```
Query  33221    AATTTAGACCACTTGGGAATAGTAGCAGGGATAATAGATGAAAT-GGAATTGGTAGAAGA  33279
                || || || |||||||||||| ||  ||| ||||||||||| || |||||| ||||||
Sbjct  2601272  AACTTGGAGCACTTGGGAATAATAAAAGGAATAATAGATGAGATAGGAATT-GTAGAAAG
2601330

Query  33280    AGTCAACAAAAAAGTGGGAATAAAAATTAAAGAAACCCTAAGCCCAGGACAAGTAATGAA  33339
                | ||||  | | | |   |||| | | || ||| |    | |    ||||| || || | ||
Sbjct  2601331  AATCAATGAGATATTCTTAATAGATAGTAGAGAGAAAGTTAATACAGGAGAAATAGTCAA  2601390

Query  33340    AGCAATGATTTTGAACGGACTGGGATTTTTGAGCGCCCCAATCTACCTATTCGATGCTTT  33399
                |||||| |||    | ||||| || ||| |  |     ||| | ||  ||||    |    ||
Sbjct  2601391  AGCAATCATT----ATGGACTAGGTTTTCTATGGAGACCATTATATTTATTTCCTCAATT  2601446

Query  33400    TTTCGTTGGAAAAGCAACAGAACATCTAATTGGAGAAGGAGTGACCCCCGAACAATTAAA  33459
                ||| |  |||||||| | ||||||| || | ||||  ||| |         ||| | |||||
Sbjct  2601447  TTTTGAGGGAAAAGCCATAGAACATTTACTAGGAGCGGGAATTGAAGGGGAAGATTTAAA
2601506

Query  33460    TGATGACAGAATCGGGAGAGCATTAGATAA  33489
                |||||| | ||| || |||| | | |||||
Sbjct  2601507  TGATGATAAAATAGGCAGAGTAATGGATAA  2601536
```

Range 14: 1777325 to 1777558

Score:77.9 bits(85), Expect:1e-12,

```
Identities:162/237(68%),  Gaps:6/237(2%), Strand: Plus/Plus

Query  34230    TCTGGGAGATTTATATTAGCCACAAATATCTTAGATACTGAAGCAGTAAGCAATCCACAG  34289
                ||||| |||||| | ||||| || ||||| |||||||||  || ||| || || ||  ||
Sbjct  1777325  TCTGGTAGATTTGTTTTAGCAACTAATATTTTAGATACTAGAG-AGTTAG-AAGCCCTAG  1777382

Query  34290    GTATTATTGGAA---TATAAAGCGCAGCAAAGTAATGAGAGAGGATTTAGATTTATCAAA  34346
                  |  | || ||   ||||||  |||||||  |    ||  ||||||||||||||||||
Sbjct  1777383  AAAC-ACTGAAAATCTATAAACAGCAGCAATCTTCAGAACGAGGATTTAGATTTATCAAA  1777441

Query  34347    GATCCGTTATTTTTTACTTCCAGTGTATTCGTGAAAAAGCCGGAGCGAGTTGAAGCAATC  34406
                || || |||||| || |    |||   || || | ||| || ||  |||| ||  ||||
Sbjct  1777442  GACCCTTTATTTATTGCAGATAGTTATTTGGTAAGAAATCCCGAAAGAGTAGAGACAATG  1777501

Query  34407    GGAATGATAATGGGACTGTGTTTGTTAGTATATAACCTGGCGCAAAGGAAATTGAGA  34463
                   ||  |||||  | ||| | | || || ||||| || |  || ||| || | |||
Sbjct  1777502  ATGATATTAATGCCATTGTTTCTTTTGGTTTATAATCTAGGACAGAGGCAACTAAGA  1777558
```

Range 15: 3506076 to 3506218

```
Score:74.3 bits(81), Expect:2e-11,
Identities:102/143(71%),  Gaps:0/143(0%), Strand: Plus/Minus

Query  33225    TAGACCACTTGGGAATAGTAGCAGGGATAATAGATGAAATGGAATTGGTAGAAGAAGTCA  33284
                ||||||| || || ||||||||||| |||||||| |  || | | | ||||||  | |||
Sbjct  3506218  TAGACCATTTAGGCATAGTAGCAGGAATAATAGACGCCATAGGAGTCGTAGAAATAATCA
3506159

Query  33285    ACAAAAAAGTGGGAATAAAAATTAAAGAAACCCTAAGCCCAGGACAAGTAATGAAAGCAA  33344
                || ||  | | ||| |  |||||  ||| |    |||| || || || ||| | ||||| |
Sbjct  3506158  ACGAATTAATAGGAGTTGAAATTGGAGAGAAAGTAAGTCCGGGTCATGTAGTAAAAGCCA
3506099

Query  33345    TGATTTTGAACGGACTGGGATTT  33367
                ||||  | |||||  | ||||||
Sbjct  3506098  TGATAATCAACGGGTTAGGATTT  3506076
```

Range 16: 3516757 to 3516899

```
Score:74.3 bits(81), Expect:2e-11,
Identities:102/143(71%),  Gaps:0/143(0%), Strand: Plus/Minus

Query  33225    TAGACCACTTGGGAATAGTAGCAGGGATAATAGATGAAATGGAATTGGTAGAAGAAGTCA  33284
                ||||||| || || ||||||||||| |||||||| |  || | | | ||||||  | |||
Sbjct  3516899  TAGACCATTTAGGCATAGTAGCAGGAATAATAGACGCCATAGGAGTCGTAGAAATAATCA
3516840

Query  33285    ACAAAAAAGTGGGAATAAAAATTAAAGAAACCCTAAGCCCAGGACAAGTAATGAAAGCAA  33344
                || ||  | | ||| |  |||||  ||| |    |||| || || || ||| | ||||| |
Sbjct  3516839  ACGAATTAATAGGAGTTGAAATTGGAGAGAAAGTAAGTCCGGGTCATGTAGTAAAAGCCA
3516780
```

```
Query  33345   TGATTTTGAACGGACTGGGATTT  33367
               ||||  | |||||  | ||||||
Sbjct  3516779  TGATAATCAACGGGTTAGGATTT  3516757
```

Range 17: 5211775 to 5211916

Score:72.5 bits(79), Expect:5e-11,
Identities:101/142(71%), Gaps:0/142(0%), Strand: Plus/Plus

```
Query  34299   GAATATAAAGCGCAGCAAAGTAATGAGAGAGGATTTAGATTTATCAAAGATCCGTTATTT  34358
               |||||||||| || ||   |  ||||||||| |||   ||| ||||||| || ||||||
Sbjct  5211775  GAATATAAAGCTCAACAGTCTTGTGAGAGAGGGTTTGCTTTTCTCAAAGACCCATTATTT  5211834

Query  34359   TTTACTTCCAGTGTATTCGTGAAAAAGCCGGAGCGAGTTGAAGCAATCGGAATGATAATG  34418
               ||| |   |||| | ||| | ||||  || ||| || | ||  |  | |||||||| |||
Sbjct  5211835  TTTGCAGACAGTATTTTCCTAAAAAGTCCAGAGAGAATAGAGTCCCTGGGAATGATTATG
5211894

Query  34419   GGACTGTGTTTGTTAGTATATA  34440
               ||  | ||| || | || ||||
Sbjct  5211895  GGTTTATGTCTGCTGGTTTATA  5211916
```

Range 18: 1381 to 1522

Score:70.7 bits(77), Expect:2e-10,
Identities:103/144(72%), Gaps:4/144(2%), Strand: Plus/Plus

```
Query  34299  GAATATAAAGCGCAGCAAAGTAATG--AGAGAGGATTTAGATTTATCAAAGATCCGTTAT  34356
              |||||||||| ||  | |||  ||  ||||||| |||   ||| ||||||| || ||||
Sbjct  1381   GAATATAAAGCTCA--ACAGTCTTGCCAGAGAGGGTTTGCTTTTCTCAAAGACCCATTAT  1438

Query  34357  TTTTTACTTCCAGTGTATTCGTGAAAAAGCCGGAGCGAGTTGAAGCAATCGGAATGATAA  34416
              ||||| |   |||| | ||| | ||||  || ||| || | ||  || | |||||||| |
Sbjct  1439   TTTTTGCAGACAGTATTTTCCTAAAAAGTCCAGAGAGAATAGAGTCACTGGGAATGATTA  1498

Query  34417  TGGGACTGTGTTTGTTAGTATATA  34440
              ||||  | ||| || | || ||||
Sbjct  1499   TGGGTTTATGTCTGCTGGTTTATA  1522
```

Range 19: 4790 to 4931

Score:70.7 bits(77), Expect:2e-10,
Identities:103/144(72%), Gaps:4/144(2%), Strand: Plus/Minus

```
Query  34299  GAATATAAAGCGCAGCAAAGTAATG--AGAGAGGATTTAGATTTATCAAAGATCCGTTAT  34356
              |||||||||| ||  | |||  ||  ||||||| |||   ||| ||||||| || ||||
Sbjct  4931   GAATATAAAGCTCA--ACAGTCTTGCCAGAGAGGGTTTGCTTTTCTCAAAGACCCATTAT  4874
```

```
Query  34357  TTTTTACTTCCAGTGTATTCGTGAAAAAGCCGGAGCGAGTTGAAGCAATCGGAATGATAA  34416
              ||||| |   |||| | ||| | ||||  || ||| || | ||  || | |||||||| |
Sbjct  4873   TTTTTGCAGACAGTATTTTCCTAAAAAGTCCAGAGAGAATAGAGTCACTGGGAATGATTA  4814

Query  34417  TGGGACTGTGTTTGTTAGTATATA  34440
              ||||  | ||| || | || ||||
Sbjct  4813   TGGGTTTATGTCTGCTGGTTTATA  4790
```

Range 20: 1064783 to 1065047

Score:69.8 bits(76), Expect:2e-10,
Identities:179/270(66%), Gaps:6/270(2%), Strand: Plus/Plus

```
Query  33221    AATTTAGACCACTTGGGAATAGTAGCAGGGATAATAGATGAAAT-GGAATTGGTAGAAGA  33279
                || || || |||||||||| | ||||||| ||||||||||| || |||| | |||| | |
Sbjct  1064783  AAATTGGAGCACTTGGGAAGAATAGCAGGAATAATAGATGAGATAGGAACT-GTAGGAAA
1064841

Query  33280    AGTCAACAAAAAAGTGGGAATAAAAATTAAAGAAACCCTAAGCCCAGGACAAGTAATGAA  33339
                | ||||  | | | |   |||| | | || ||| |    | |   ||||| || || | ||
Sbjct  1064842  AATCAATGAGATATTCTTAATAGATAGTAGAGAGAAAGTGACTACAGGAGAAATAGTCAA
1064901

Query  33340    AGCAATGATTTTGAACGGACTGGGATTTTTGAGCGCCCCAATCTACCTATTCGATGCTTT  33399
                |||||| |||    | ||||| || ||| |  |    ||| | ||  ||||    |    ||
Sbjct  1064902  AGCAATCATT----ATGGACTAGGTTTTCTATGGAGACCATTATATTTATTTCCTCGATT  1064957

Query  33400    TTTCGTTGGAAAAGCAACAGAACATCTAATTGGAGAAGGAGTGACCCCCGAACAATTAAA  33459
                ||  |    |||||| | ||||||| || | ||||  ||| |      |||| | |||||
Sbjct  1064958  TTCTGAGAAAAAAGCCATAGAACATTTATTAGGAGTGGGAATTGAAGGCGAAGATTTAAA
1065017

Query  33460    TGATGACAGAATCGGGAGAGCATTAGATAA  33489
                |||||| | ||| || |||| | | |||||
Sbjct  1065018  TGATGATAAAATAGGCAGAGTAATGGATAA  1065047
```

Range 21: 1075425 to 1075689

Score:69.8 bits(76), Expect:2e-10,
Identities:179/270(66%), Gaps:6/270(2%), Strand: Plus/Plus

```
Query  33221    AATTTAGACCACTTGGGAATAGTAGCAGGGATAATAGATGAAAT-GGAATTGGTAGAAGA  33279
                || || || |||||||||| | ||||||| ||||||||||| || |||| | |||| | |
Sbjct  1075425  AAATTGGAGCACTTGGGAAGAATAGCAGGAATAATAGATGAGATAGGAACT-GTAGGAAA
1075483

Query  33280    AGTCAACAAAAAAGTGGGAATAAAAATTAAAGAAACCCTAAGCCCAGGACAAGTAATGAA  33339
                | ||||  | | | |   |||| | | || ||| |    | |   ||||| || || | ||
Sbjct  1075484  AATCAATGAGATATTCTTAATAGATAGTAGAGAGAAAGTGACTACAGGAGAAATAGTCAA
1075543
```

```
Query  33340    AGCAATGATTTTGAACGGACTGGGATTTTTGAGCGCCCCAATCTACCTATTCGATGCTTT  33399
                |||||| |||    | ||||| || ||| |  |    ||| | ||  ||||   |   ||
Sbjct  1075544  AGCAATCATT----ATGGACTAGGTTTTCTATGGAGACCATTATATTTATTTCCTCGATT  1075599

Query  33400    TTTCGTTGGAAAAGCAACAGAACATCTAATTGGAGAAGGAGTGACCCCCGAACAATTAAA  33459
                ||  |    |||||| | ||||||| || | ||||  ||| |      |||| | |||||
Sbjct  1075600  TTCTGAGAAAAAAGCCATAGAACATTTATTAGGAGTGGGAATTGAAGGCGAAGATTTAAA
1075659

Query  33460    TGATGACAGAATCGGGAGAGCATTAGATAA  33489
                |||||| | ||| || |||| | | |||||
Sbjct  1075660  TGATGATAAAATAGGCAGAGTAATGGATAA  1075689
```

Range 22: 665424 to 665565

Score:68.0 bits(74), Expect:6e-10,
Identities:100/142(70%), Gaps:0/142(0%), Strand: Plus/Plus

```
Query  34299   GAATATAAAGCGCAGCAAAGTAATGAGAGAGGATTTAGATTTATCAAAGATCCGTTATTT  34358
               ||||||||||| || ||   |   |||||||| |||   ||| ||||||| || ||||||
Sbjct  665424  GAATATAAAGCTCAACAGTCTTGCGAGAGAGGGTTTGCTTTTCTCAAAGACCCATTATTT  665483

Query  34359   TTTACTTCCAGTGTATTCGTGAAAAAGCCGGAGCGAGTTGAAGCAATCGGAATGATAATG  34418
               ||| |   |||| | ||| | ||||  || ||| || | ||  |  | |||||||| |||
Sbjct  665484  TTTGCAGACAGTATTTTCCTAAAAAGTCCAGAGAGAATAGAGTCCCTGGGAATGATTATG  665543

Query  34419   GGACTGTGTTTGTTAGTATATA  34440
               ||  | ||| || | || ||||
Sbjct  665544  GGTTTATGTCTGCTGGTTTATA  665565
```

Range 23: 4375978 to 4376130

Score:67.1 bits(73), Expect:2e-09,
Identities:111/156(71%), Gaps:6/156(3%), Strand: Plus/Plus

```
Query  34299    GAATATAAAGCGCAGCAAAGTAATG--AGAGAGGATTTAGATTTATCAAAGATCCGTTAT  34356
                ||||||||||| ||  | |||  ||  ||||||| |||   ||| ||||||| || ||||
Sbjct  4375978  GAATATAAAGCTCA--ACAGTCTTGCCAGAGAGGGTTTGCTTTTCTCAAAGACCCATTAT  4376035

Query  34357    TTTTTACTTCCAGTGTATTCGTGAAAAAGCCGGAGCGAGTTGAAGCAATCGGAATGATAA  34416
                ||||| |   |||| | ||| | ||||  || ||| || | ||  |  | |||||||| |
Sbjct  4376036  TTTTTGCAGACAGTATTTTCCTAAAAAGTCCAGAGAGAATAGAGTCCCTGGGAATGATTA  4376095

Query  34417    TGGGACTGTGTTTGTTAGTATATAACCTG-GCGCAA  34451
                ||||  | ||| || | || ||| || || ||||||
Sbjct  4376096  TGGGTTTATGTCTGCTGGTTTAT-ACTTGAGCGCAA  4376130
```

```
Range 24: 410238 to 410379

Score:66.2 bits(72), Expect:2e-09,
Identities:102/144(71%),  Gaps:4/144(2%), Strand: Plus/Plus

Query  34299   GAATATAAAGCGCAGCAAAGTAATG--AGAGAGGATTTAGATTTATCAAAGATCCGTTAT  34356
               ||||||||||| ||  | |||  ||  ||||||| |||   ||| ||||||| || ||||
Sbjct  410238  GAATATAAAGCTCA--ACAGTCTTGCCAGAGAGGGTTTGCTTTTCTCAAAGACCCATTAT  410295

Query  34357   TTTTTACTTCCAGTGTATTCGTGAAAAAGCCGGAGCGAGTTGAAGCAATCGGAATGATAA  34416
               ||||| |   |||| | ||| | ||||  || ||| || | ||  |  | |||||||| |
Sbjct  410296  TTTTTGCAGACAGTATTTTCCTAAAAATTCCAGAGAGAATAGAGTCCCTGGGAATGATTA  410355

Query  34417   TGGGACTGTGTTTGTTAGTATATA  34440
               ||||  | ||| || | || ||||
Sbjct  410356  TGGGTTTATGTCTGCTGGTTTATA  410379



Range 25: 2251046 to 2251187

Score:66.2 bits(72), Expect:2e-09,
Identities:102/144(71%),  Gaps:4/144(2%), Strand: Plus/Plus

Query  34299    GAATATAAAGCGCAGCAAAGTAATG--AGAGAGGATTTAGATTTATCAAAGATCCGTTAT  34356
                ||||||||||| ||  | |||  ||  ||||||| |||   ||| ||||||| || ||||
Sbjct  2251046  GAATATAAAGCTCA--ACAGTCTTGCTAGAGAGGGTTTGCTTTTCTCAAAGACCCATTAT  2251103

Query  34357    TTTTTACTTCCAGTGTATTCGTGAAAAAGCCGGAGCGAGTTGAAGCAATCGGAATGATAA  34416
                ||||| |   |||| | ||| | ||||  || ||| || | ||  |  | |||||||| |
Sbjct  2251104  TTTTTGCAGACAGTATTTTCCTAAAAAGTCCAGAGAGAATAGAGTCCCTGGGAATGATTA  2251163

Query  34417    TGGGACTGTGTTTGTTAGTATATA  34440
                ||||  | ||| || | || ||||
Sbjct  2251164  TGGGTTTATGTCTGCTGGTTTATA  2251187



Range 26: 2464132 to 2464273

Score:66.2 bits(72), Expect:2e-09,
Identities:102/144(71%),  Gaps:4/144(2%), Strand: Plus/Plus

Query  34299    GAATATAAAGCGCAGCAAAGTAATG--AGAGAGGATTTAGATTTATCAAAGATCCGTTAT  34356
                ||||||||||| ||  | |||  ||  ||||||| |||   ||| ||||||| || ||||
Sbjct  2464132  GAATATAAAGCTCA--ACAGTCTTGCCAGAGAGGGTTTGCTTTTCTCAAAGACCCATTAT  2464189

Query  34357    TTTTTACTTCCAGTGTATTCGTGAAAAAGCCGGAGCGAGTTGAAGCAATCGGAATGATAA  34416
                ||||| |   |||  | ||| | ||||  || ||| || | ||  |  | |||||||| |
Sbjct  2464190  TTTTTGCAGACAGGATTTTCCTAAAAAGTCCAGAGAGAATAGAGTCCCTGGGAATGATTA
2464249

Query  34417    TGGGACTGTGTTTGTTAGTATATA  34440
                ||||  | ||| || |||| ||||
Sbjct  2464250  TGGGTTTATGTCTGCTAGTTTATA  2464273
```

Range 27: 4961118 to 4961355

Score:66.2 bits(72), Expect:2e-09,
Identities:162/243(67%), Gaps:6/243(2%), Strand: Plus/Plus

```
Query  33248    GGGATAATAGATGAAAT-GGAATTGGTAGAAGAAGTCAACAAAAAAGTGGGAATAAAAAT  33306
                || ||||||||||| || |||||| |||||| || |||| || | | |  ||||| | |
Sbjct  4961118  GGAATAATAGATGACATAGGAATT-GTAGAAAAAATCAATAAGATATTCTGAATAGATAG  4961176

Query  33307    TAAAGAAACCCTAAGCCCAGGACAAGTAATGAAAGCAATGATTTTGAACGGACTGGGATT  33366
                || ||| |   | |   ||||| || || | |||||||| |||    | ||||| || ||
Sbjct  4961177  TAGAGAGAAAGTGAATACAGGAGAAATAGTCAAAGCAATCATT----ATGGACTAGGTTT  4961232

Query  33367    TTTGAGCGCCCCAATCTACCTATTCGATGCTTTTTTCGTTGGAAAAGCAACAGAACATCT  33426
                | |  |    ||| | ||  ||||   |    |||| |  | |||||| | |||| || |
Sbjct  4961233  TCTATGGAGACCATTATATTTATTTCCTCAACTTTTTGAGGAAAAAGCTATAGAATATTT  4961292

Query  33427    AATTGGAGAAGGAGTGACCCCCGAACAATTAAATGATGACAGAATCGGGAGAGCATTAGA  33486
                | | ||||  ||| |      |||| | ||||||||||| ||||| || |||| | | ||
Sbjct  4961293  ATTAGGAGCGGGAATTGAAGGCGAAGATTTAAATGATGATAGAATAGGCAGAGTAATGGA
4961352

Query  33487    TAA  33489
                |||
Sbjct  4961353  TAA  4961355
```

Range 28: 377480 to 377549

Score:63.5 bits(69), Expect:3e-08,
Identities:58/71(82%), Gaps:2/71(2%), Strand: Plus/Plus

```
Query  1965     TTAATGTGGTTTCAAAAATCCTCTGTTTGCAGTTTTGACCTCT-GCTTTTTGCAGTTCGC  2023
                ||||| ||||||||||| | |  ||||||||||||| | |||| | | ||||||||||||
Sbjct  377480   TTAATTTGGTTTCAAAATTGCGTTGTTTGCAGTTTT-ATCTCTGGATGTTTGCAGTTCGC  377538

Query  2024     TACACTTAGGG  2034
                || |  |||||
Sbjct  377539   TAAAGATAGGG  377549
```

Range 29: 2231487 to 2231628

Score:63.5 bits(69), Expect:3e-08,
Identities:99/142(70%), Gaps:0/142(0%), Strand: Plus/Minus

```
Query  34299    GAATATAAAGCGCAGCAAAGTAATGAGAGAGGATTTAGATTTATCAAAGATCCGTTATTT  34358
                ||||||||||| ||  |   |   |||||||| |||   ||| ||||||| || ||||||
Sbjct  2231628  GAATATAAAGCTCAATACTCTTGCGAGAGAGGGTTTGCTTTTCTCAAAGACCCATTATTT  2231569
```

Query 34359   TTTACTTCCAGTGTATTCGTGAAAAAGCCGGAGCGAGTTGAAGCAATCGGAATGATAATG 34418
                 ||| | |||| | ||| | |||| || ||| || | || | | |||||||| |||
Sbjct 2231568 TTTGCAGACAGTATTTTCCTAAAAAGTCCAGAGAGAATAGAGTCCCTGGGAATGATTATG
2231509

Query 34419   GGACTGTGTTTGTTAGTATATA 34440
                 || | ||| || | || ||||
Sbjct 2231508 GGTTTATGTCTGCTGGTTTATA 2231487

Range 30: 2498833 to 2498974

Score:63.5 bits(69), Expect:3e-08,
Identities:99/142(70%), Gaps:0/142(0%), Strand: Plus/Minus

Query 34299   GAATATAAAGCGCAGCAAAGTAATGAGAGAGGATTTAGATTTATCAAAGATCCGTTATTT 34358
                 ||||||||||| || || | |||||||| ||| ||| ||||||| || ||||||
Sbjct 2498974 GAATATAAAGCTCAACAGTCTTGCGAGAGAGGGTTTGCTTTTCTCAAAGACCCATTATTT 2498915

Query 34359   TTTACTTCCAGTGTATTCGTGAAAAAGCCGGAGCGAGTTGAAGCAATCGGAATGATAATG 34418
                 ||| | |||| | ||| | |||| || ||| || | || | | |||||| | |||
Sbjct 2498914 TTTGCAGACAGTATTTTCCTAAAAAGTCCAGAGAGAATAGAGTCCCTGGGAATGGTGATG
2498855

Query 34419   GGACTGTGTTTGTTAGTATATA 34440
                 || | ||| || | || ||||
Sbjct 2498854 GGTTTATGTCTGCTGGTTTATA 2498833

Range 31: 3824297 to 3824431

Score:63.5 bits(69), Expect:3e-08,
Identities:97/136(71%), Gaps:2/136(1%), Strand: Plus/Plus

Query 33221   AATTTAGACCACTTGGGAATAGTAGCAGGGATAATAGATGAAAT-GGAATTGGTAGAAGA 33279
                 || ||||| || ||||||||| || |||| ||||||||||| || |||||| ||||||
Sbjct 3824297 AACTTAGATCAATTGGGAATAATAACAGGAATAATAGATGACATAGGAATT-GTAGAAAG 3824355

Query 33280   AGTCAACAAAAAAGTGGGAATAAAAATTAAAGAAACCCTAAGCCCAGGACAAGTAATGAA 33339
                 | |||| | | | | |||| | | || ||| | | | ||||| || || | ||
Sbjct 3824356 AATCAATGAGATATTCTTAATAGAGAGTAGAGAGAAAGTGAATGCAGGAGAAATAGTCAA
3824415

Query 33340   AGCAATGATTTTGAAC 33355
                 |||||| ||| || ||
Sbjct 3824416 AGCAATCATTATGGAC 3824431

Range 32: 2319144 to 2319296

```
Score:62.6 bits(68), Expect:3e-08,
Identities:110/156(71%),  Gaps:6/156(3%), Strand: Plus/Plus

Query  34299    GAATATAAAGCGCAGCAAAGTAATG--AGAGAGGATTTAGATTTATCAAAGATCCGTTAT  34356
                ||||||||||| ||  | |||  ||  ||||||| |||    ||| ||||||| || ||||
Sbjct  2319144  GAATATAAAGCTCA--ACAGTCTTGCCAGAGAGGGTTTGCTTTTCTCAAAGACCCATTAT  2319201

Query  34357    TTTTTACTTCCAGTGTATTCGTGAAAAAGCCGGAGCGAGTTGAAGCAATCGGAATGATAA  34416
                ||||| |   |||| | ||| | ||||  || ||| || | ||  |  | |||||||| |
Sbjct  2319202  TTTTTGCAGACAGTATTTTCCTAAAAAGTCCAGAGAGAATAGAGTCCCTGGGAATGATGA
2319261

Query  34417    TGGGACTGTGTTTGTTAGTATATAACCTG-GCGCAA  34451
                ||||  | | | || | || ||| || || ||||||
Sbjct  2319262  TGGGTTTATCTCTGCTGGTTTAT-ACTTGAGCGCAA  2319296



Range 33: 3578266 to 3578492

Score:62.6 bits(68), Expect:3e-08,
Identities:157/233(67%),  Gaps:12/233(5%), Strand: Plus/Minus

Query  34237    GATTTATATTAGCCACAAATATCTTAGATACTGAAGCAGTAAGCAATCCACAGGTATTAT  34296
                ||||||| ||||| | |||||| |||||  |  | | | | || || ||| ||  || ||
Sbjct  3578492  GATTTATTTTAGCAAAAAAATATTTTAGAGGCA-AGGGAATTAGAAA-CCATAGAAAT-AT  3578436

Query  34297    TG-GAATATAAAGCGCAGCAAAGTAATGAG-----AGAGGATTTAGATTTATCAAAGATC  34350
                || ||||||| || | ||    ||||| |      | |||||| ||| |||||||| | |
Sbjct  3578435  TGAGAATATATAGAGAAGG---GTAATCAACAAAAATAGGATTGAGAGTTATCAAATACC  3578379

Query  34351    CGTTATTTTTTACTTCCAGTGTATTCGTGAAAAAGCCGGAGCGAGTTGAAGCAATCGGAA  34410
                ||||||||||  |    ||| | ||  ||||| | || ||  |||| ||  ||||     |
Sbjct  3578378  CGTTATTTTTCGCCCATAGTCTTTTTATGAAAGATCCAGAAAGAGTAGAGACAATGATGA  3578319

Query  34411    TGATAATGGGACTGTGTTTGTTAGTATATAACCTGGCGCAAAGGAAATTGAGA  34463
                || |||||| | ||||| | || || |||     |||  |||||  |||| |||
Sbjct  3578318  TGTTAATGGCATTGTGTCTTTTGGTTTATTGTGTGGGACAAAGACAATTAAGA  3578266



Range 34: 216492 to 216633

Score:61.7 bits(67), Expect:9e-08,
Identities:101/144(70%),  Gaps:4/144(2%), Strand: Plus/Plus

Query  34299   GAATATAAAGCGCAGCAAAGTAATG--AGAGAGGATTTAGATTTATCAAAGATCCGTTAT  34356
               ||||||||||| ||  | |||  ||  ||||||| |||    ||| ||||||| || ||||
Sbjct  216492  GAATATAAAGCTCA--ACAGTCTTGCCAGAGAGGGTTTGCTTTTCTCAAAGACCCATTAT  216549

Query  34357   TTTTTACTTCCAGTGTATTCGTGAAAAAGCCGGAGCGAGTTGAAGCAATCGGAATGATAA  34416
               ||||| |   |||| | ||| | ||||  || ||| || | ||  |  | || ||||| |
Sbjct  216550  TTTTTGCAGACAGTATTTTCCTAAAAAGTCCAGAGAGAATAGAGTCCCTGGGCATGATGA  216609

Query  34417   TGGGACTGTGTTTGTTAGTATATA  34440
```

```
         |||| | ||| || | || ||||
Sbjct  216610  TGGGTTTATGTCTGCTGGTTTATA  216633
```

Range 35: 2358112 to 2358307

Score:61.7 bits(67), Expect:9e-08,
Identities:143/205(70%), Gaps:17/205(8%), Strand: Plus/Plus

```
Query  33756    ATGAAATGGATAACGAGAGTACCATTAAGCATCAAAGAAGCTCAAAATAAAATCTGCAAG  33815
                ||||||||||||   ||||||| |||  ||| ||| ||||  ||||     ||   |||
Sbjct  2358112  ATGAAATGGATAAGTTGAGTACCTTTATCCATTAAAAAAGCCAAAAACTTGGTCA--AAG  2358169

Query  33816    -ATTGAAGA-TAGTGC-CTGGGAAAACCC--TGAAATAAAAGGATATA---AAATAGCAG  33867
                  ||| || | || | |||   |||||   |||||| ||||||||||    | | || ||
Sbjct  2358170  CATTTAACAGTACAGAACTG---AAACCAGTTGAAATCAAAGGATATAGCTATAAAGAAG  2358226

Query  33868    TCCAAACGAGTGATTATGCTGGCATCAAGCAAAGATGGCTAGTAATAGAAAGTAAAATCA  33927
                    ||| |  |  |||||  || ||  ||||||||||||| || ||||||||  |  ||
Sbjct  2358227  A--AAAAGTATC-TTATGGGGGAATAGAGCAAAGATGGCTATTAGTAGAAAGTGCAGACA  2358283

Query  33928    GAAAGCAAGCAGATCTTaaaaaaaT  33952
                ||||  ||||||| |||||| ||||
Sbjct  2358284  GAAAAAAAGCAGA-CTTAAAGAAAT  2358307
```

Range 36: 5289347 to 5289488

Score:61.7 bits(67), Expect:9e-08,
Identities:101/144(70%), Gaps:4/144(2%), Strand: Plus/Minus

```
Query  34299    GAATATAAAGCGCAGCAAAGTAATG--AGAGAGGATTTAGATTTATCAAAGATCCGTTAT  34356
                |||||||||| ||  | |||  ||  ||||||| |||   ||| ||||||| || ||||
Sbjct  5289488  GAATATAAAGCTCA--ACAGTCTTGCCAGAGAGGGTTTGCTTTTCTCAAAGACCCATTAT  5289431

Query  34357    TTTTTACTTCCAGTGTATTCGTGAAAAAGCCGGAGCGAGTTGAAGCAATCGGAATGATAA  34416
                || || |   |||| | ||| | ||||  || ||| || | ||  |  | |||||||| |
Sbjct  5289430  TTCTTGCAGACAGTATTTTCCTAAAAAGTCCAGAGAGAATAGAGTCCCTGGGAATGATTA  5289371

Query  34417    TGGGACTGTGTTTGTTAGTATATA  34440
                ||||  | ||| || | || ||||
Sbjct  5289370  TGGGTTTATGTCTGCTGGTTTATA  5289347
```

Range 37: 4568585 to 4568705

Score:59.9 bits(65), Expect:3e-07,
Identities:88/123(72%), Gaps:4/123(3%), Strand: Plus/Plus

```
Query  34299    GAATATAAAGCGCAGCAAAGTAATG--AGAGAGGATTTAGATTTATCAAAGATCCGTTAT  34356
                |||||||||| ||  | |||  ||  ||||||| |||   ||| ||||||| || ||||
```

```
Sbjct  4568585  GAATATAAAGCTCA--ACAGTCTTGCCAGAGAGGGTTTGCTTTTCTCAAAGACCCATTAT  4568642

Query  34357    TTTTTACTTCCAGTGTATTCGTGAAAAAGCCGGAGCGAGTTGAAGCAATCGGAATGATAA  34416
                ||||| |   |||| | ||| | ||||  || ||| || | ||  |  | |||||||| |
Sbjct  4568643  TTTTTGCAGACAGTATTTTCCTAAAAAGTCCAGAGAGAATAGATTCCCTGGGAATGATTA  4568702

Query  34417    TGG  34419
                |||
Sbjct  4568703  TGG  4568705
```

Range 38: 1897631 to 1897772

Score:59.0 bits(64), Expect:3e-07,
Identities:98/142(69%), Gaps:0/142(0%), Strand: Plus/Minus

```
Query  34299    GAATATAAAGCGCAGCAAAGTAATGAGAGAGGATTTAGATTTATCAAAGATCCGTTATTT  34358
                ||||||||||| || ||   |   ||||| || |||   ||| ||||||| || ||||||
Sbjct  1897772  GAATATAAAGCTCAACAGTCTTGCGAGAGGGGGTTTGCTTTTCTCAAAGACCCATTATTT  1897713

Query  34359    TTTACTTCCAGTGTATTCGTGAAAAAGCCGGAGCGAGTTGAAGCAATCGGAATGATAATG  34418
                ||| |   |||| | ||| | || |  || ||  || | ||  || | |||||||| |||
Sbjct  1897712  TTTGCAGACAGTATTTTCTTAAATAGTCCAGATAGAATGGAGTCACTGGGAATGATGATG
1897653

Query  34419    GGACTGTGTTTGTTAGTATATA  34440
                ||  | ||| || | || ||||
Sbjct  1897652  GGTTTATGTCTGCTGGTTTATA  1897631
```

Range 39: 3422843 to 3422995

Score:59.0 bits(64), Expect:3e-07,
Identities:107/155(69%), Gaps:4/155(2%), Strand: Plus/Minus

```
Query  34299    GAATATAAAGCGCAGCAAAGTAATG--AGAGAGGATTTAGATTTATCAAAGATCCGTTAT  34356
                ||||||||||| ||  | |||  ||  ||||||| |||    ||| ||||||| || ||||
Sbjct  3422995  GAATATAAAGCTCA--ACAGTCTTGCCAGAGAGGGTTTGCTTTTCTCAAAGACCCATTAT  3422938

Query  34357    TTTTTACTTCCAGTGTATTCGTGAAAAAGCCGGAGCGAGTTGAAGCAATCGGAATGATAA  34416
                ||| | |   |||| | ||| | ||||  || ||| || | ||  |  | |||||||| |
Sbjct  3422937  TTTGTGCAGACAGTATTTTCCTAAAAAGTCCAGAGAGAATAGAGTCCCTGGGAATGATTA
3422878

Query  34417    TGGGACTGTGTTTGTTAGTATATAACCTGGCGCAA  34451
                ||||  | | | || | || ||||   | ||||||
Sbjct  3422877  TGGGTTTATATCTGCTGGTTTATACTTTAGCGCAA  3422843
```

Range 40: 3464726 to 3464878

```
Score:59.0 bits(64), Expect:3e-07,
Identities:107/155(69%),  Gaps:4/155(2%), Strand: Plus/Minus

Query  34299    GAATATAAAGCGCAGCAAAGTAATG--AGAGAGGATTTAGATTTATCAAAGATCCGTTAT  34356
                ||||||||||| ||  | |||  ||  ||||||| |||   ||| ||||||| || ||||
Sbjct  3464878  GAATATAAAGCTCA--ACAGTCTTGCCAGAGAGGGTTTGCTTTTCTCAAAGACCCATTAT  3464821

Query  34357    TTTTTACTTCCAGTGTATTCGTGAAAAAGCCGGAGCGAGTTGAAGCAATCGGAATGATAA  34416
                ||| | |   |||| | ||| | ||||  || ||| || | ||  |  | |||||||| |
Sbjct  3464820  TTTGTGCAGACAGTATTTTCCTAAAAAGTCCAGAGAGAATAGAGTCCCTGGGAATGATTA
3464761

Query  34417    TGGGACTGTGTTTGTTAGTATATAACCTGGCGCAA  34451
                ||||  | | | || | || ||||   | ||||||
Sbjct  3464760  TGGGTTTATATCTGCTGGTTTATACTTTAGCGCAA  3464726
```

Range 41: 4597311 to 4597432

```
Score:59.0 bits(64), Expect:3e-07,
Identities:86/122(70%),  Gaps:0/122(0%), Strand: Plus/Plus

Query  34299    GAATATAAAGCGCAGCAAAGTAATGAGAGAGGATTTAGATTTATCAAAGATCCGTTATTT  34358
                |||||||||||  | ||   |   |||||||| ||| | ||| ||||||| || ||||||
Sbjct  4597311  GAATATAAAGCTGAACAGTCTTGCGAGAGAGGGTTTGGTTTTCTCAAAGACCCATTATTT
4597370

Query  34359    TTTACTTCCAGTGTATTCGTGAAAAAGCCGGAGCGAGTTGAAGCAATCGGAATGATAATG  34418
                ||  |   |||| | ||| | ||||  || ||| || | ||  |  | |||||||| |||
Sbjct  4597371  TTCGCAGACAGTATTTTCCTAAAAAGTCCAGAGAGAATAGAGTCCCTGGGAATGATGATG
4597430

Query  34419    GG  34420
                ||
Sbjct  4597431  GG  4597432
```

Range 42: 373574 to 373642

```
Score:58.1 bits(63), Expect:1e-06,
Identities:54/69(78%),  Gaps:0/69(0%), Strand: Plus/Minus

Query  1965    TTAATGTGGTTTCAAAAATCCTCTGTTTGCAGTTTTGACCTCTGCTTTTTGCAGTTCGCT  2024
               ||||| ||||||||||| | |  |||||||||||||  | |  | | ||||||||| |||
Sbjct  373642  TTAATTTGGTTTCAAAATTGCCTTGTTTGCAGTTTTATCTTTGGATGTTTGCAGTTTGCT  373583

Query  2025    ACACTTAGG  2033
               |||  ||||
Sbjct  373582  ACATCTAGG  373574
```

Range 43: 904038 to 904158

Score:58.1 bits(63), Expect:1e-06,
Identities:89/124(72%), Gaps:5/124(4%), Strand: Plus/Plus

```
Query  34299   GAATATAAAGCGCAGCAAAGTAATG--AGAGAGGATTTAGATTTATCAAAGATCCGTTAT  34356
               ||||||||||| ||  | |||  ||  ||||||| |||   ||| |||||||||| |||
Sbjct  904038  GAATATAAAGCTCA--ACAGTCTTGCCAGAGAGGGTTTGCTTTTCTCAAAGATCCATTA-  904094

Query  34357   TTTTTACTTCCAGTGTATTCGTGAAAAAGCCGGAGCGAGTTGAAGCAATCGGAATGATAA  34416
               ||||| |   |||| | ||| | ||||  || ||| || | ||  |  | |||||||| |
Sbjct  904095  TTTTTGCAGACAGTATTTTCCTAAAAAGTCCAGAGAGAATAGAGTCCCTGGGAATGATTA  904154

Query  34417   TGGG  34420
               ||||
Sbjct  904155  TGGG  904158
```

Range 44: 2009554 to 2009612

Score:58.1 bits(63), Expect:1e-06,
Identities:48/59(81%), Gaps:0/59(0%), Strand: Plus/Plus

```
Query  13970    ATTTGGCTTATATTCTTTATACATCTGGCTCGACAGGAGTCCCGAAAGGGGTTATGATT  14028
                |||| || |||||| ||||||||||||| || ||||||   || ||||| ||||| |||
Sbjct  2009554  ATTTAGCCTATATTATTTATACATCTGGATCAACAGGAAAGCCCAAAGGAGTTATCATT  2009612
```

Range 45: 4056303 to 4056455

Score:58.1 bits(63), Expect:1e-06,
Identities:109/156(70%), Gaps:6/156(3%), Strand: Plus/Plus

```
Query  34299    GAATATAAAGCGCAGCAAAGTAATG--AGAGAGGATTTAGATTTATCAAAGATCCGTTAT  34356
                ||||||||||| ||  | |||  ||  ||||||| |||   ||| ||||||| || ||||
Sbjct  4056303  GAATATAAAGCTCA--ACAGTCTTGCCAGAGAGGGTTTGCTTTTCTCAAAGACCCATTAT  4056360

Query  34357    TTTTTACTTCCAGTGTATTCGTGAAAAAGCCGGAGCGAGTTGAAGCAATCGGAATGATAA  34416
                ||| | |   |||| | ||| | ||||  || ||| || | ||  |  | |||||||| |
Sbjct  4056361  TTTGTGCAGACAGTATTTTCCTAAAAAGTCCAGAGAGAATAGAGTCCCTGGGAATGATTA
4056420

Query  34417    TGGGACTGTGTTTGTTAGTATATAACCTG-GCGCAA  34451
                ||||  | | | || | || ||| || || ||||||
Sbjct  4056421  TGGGTTTATATCTGCTGGTTTAT-ACTTGAGCGCAA  4056455
```

Range 46: 5350806 to 5350926

Score:58.1 bits(63), Expect:1e-06,
Identities:89/124(72%), Gaps:5/124(4%), Strand: Plus/Minus

```
Query  34299    GAATATAAAGCGCAGCAAAGTAATG--AGAGAGGATTTAGATTTATCAAAGATCCGTTAT  34356
                |||||||||| ||  | |||  ||  |||||| |||   ||| |||||||||| |||
Sbjct  5350926  GAATATAAAGCTCA--ACAGTCTTGCCAGAGAGGGTTTGCTTTTCTCAAAGATCCATTA-  5350870

Query  34357    TTTTTACTTCCAGTGTATTCGTGAAAAAGCCGGAGCGAGTTGAAGCAATCGGAATGATAA  34416
                ||||| |   |||| | ||| | ||||  || ||| || | ||  |  | |||||||| |
Sbjct  5350869  TTTTTGCAGACAGTATTTTCCTAAAAAGTCCAGAGAGAATAGAGTCCCTGGGAATGATTA  5350810

Query  34417    TGGG  34420
                ||||
Sbjct  5350809  TGGG  5350806
```

Range 47: 905075 to 905312

Score:57.2 bits(62), Expect:1e-06,
Identities:160/243(66%), Gaps:6/243(2%), Strand: Plus/Plus

```
Query  33248   GGGATAATAGATGAAAT-GGAATTGGTAGAAGAAGTCAACAAAAAAGTGGGAATAAAAAT  33306
               || ||||||||||| || ||||||| |||||||| | || || | | |  ||||| | |
Sbjct  905075  GGAATAATAGATGAGATAGGAATTG-TAGAAGAAATAAATAAGATATTCTGAATAGATAG  905133

Query  33307   TAAAGAAACCCTAAGCCCAGGACAAGTAATGAAAGCAATGATTTTGAACGGACTGGGATT  33366
               || ||| |   | |   ||||| || || | |||||||| ||| ||    |||| || |
Sbjct  905134  TAGAGAGAAAGTGAATACAGGAGAAATAGTCAAAGCAATCATTATG----GACTAGGTTC  905189

Query  33367   TTTGAGCGCCCCAATCTACCTATTCGATGCTTTTTTCGTTGGAAAAGCAACAGAACATCT  33426
               | |  |    ||| | ||  ||||    |    ||||||  | |||||| | ||||||| |
Sbjct  905190  TCTATGGAGACCATTATATTTATTTCCTCAACTTTTCGAGGAAAAAGCCATAGAACATTT  905249

Query  33427   AATTGGAGAAGGAGTGACCCCCGAACAATTAAATGATGACAGAATCGGGAGAGCATTAGA  33486
               | | ||||  | | |       |||| | ||||||||||| | ||| || |||  | | ||
Sbjct  905250  ATTAGGAGCGGTAATTGAAGGCGAAGATTTAAATGATGATAAAATAGGCAGACTAATGGA  905309

Query  33487   TAA  33489
               |||
Sbjct  905310  TAA  905312
```

Range 48: 791365 to 791525

Score:56.3 bits(61), Expect:4e-06,
Identities:111/162(69%), Gaps:2/162(1%), Strand: Plus/Plus

```
Query  34299   GAATATAAAGCGCAGCAAAGTAATGAGAGAGGATTTAGATTTATCAAAGATCCGTTATTT  34358
               ||||||| ||||||||||      ||| ||||||||||| ||| |  || | || ||||||
Sbjct  791365  GAATATACAGCGCAGCAATCGTGTGAAAGAGGATTTAGTTTTCTAGAAAACCCCTTATTT  791424

Query  34359   TTTACTTCCAGTGTATTCGTGAAAAAGCCGGAGCGAGTTGAAGCAATCGGAATGATAATG   34418
               |       ||||| ||  || |||  || ||   | | |||||| | | | ||| ||||
Sbjct  791425  CTAGGGCATAGTGT-TTTTTGGAAAGTCCAGAAACAATAGAAGCATTAGCAGTGAGAATG  791483
```

```
Query  34419   GGACTGTGTTTGTTAGTATATAACCTGGCGCAAAG-GAAATT  34459
               ||  | |||||  |||| || |  || || ||||| ||||||
Sbjct  791484  GGGTTTTGTTTACTAGTTTACACTCTAGCACAAAGAGAAATT  791525
```

Range 49: 2973384 to 2973454

Score:55.4 bits(60), Expect:4e-06,
Identities:56/72(78%), Gaps:1/72(1%), Strand: Plus/Plus

```
Query  33881    TTATGCTGGCATCAAGCAAAGATGGCTAGTAATAGAAAGTAAAATCAGAAAGCAAGCAGA  33940
                |||||  || ||  ||||||||||||||  | ||||||||  |  ||||||  |||||||
Sbjct  2973384  TTATGGGGGAATAGAGCAAAGATGGCTATGAGTAGAAAGTGCAGACAGAAAAAAAGCAGA
2973443

Query  33941    TCTTaaaaaaaT  33952
                 |||||| ||||
Sbjct  2973444  -CTTAAAGAAAT  2973454
```

Range 50: 4412687 to 4412807

Score:55.4 bits(60), Expect:4e-06,
Identities:87/123(71%), Gaps:4/123(3%), Strand: Plus/Plus

```
Query  34299    GAATATAAAGCGCAGCAAAGTAATG--AGAGAGGATTTAGATTTATCAAAGATCCGTTAT  34356
                ||||||||||| ||  | |||  ||  ||||||| |||   ||| ||||||| || ||||
Sbjct  4412687  GAATATAAAGCTCA--ACAGTCTTGCCAGAGAGGGTTTGCTTTTCTCAAAGACCCATTAT  4412744

Query  34357    TTTTTACTTCCAGTGTATTCGTGAAAAAGCCGGAGCGAGTTGAAGCAATCGGAATGATAA  34416
                ||||  |   |||| | ||| | ||||  || ||| || | ||  |  | |||||||| |
Sbjct  4412745  TTTTCGCAGACAGTATTTTCCTAAAAAGTCCAGAGAGAATAGAGTCCCTTGGAATGATTA  4412804

Query  34417    TGG  34419
                |||
Sbjct  4412805  TGG  4412807
```

Range 51: 1816648 to 1816769

Score:54.5 bits(59), Expect:1e-05,
Identities:85/122(70%), Gaps:0/122(0%), Strand: Plus/Minus

```
Query  34299    GAATATAAAGCGCAGCAAAGTAATGAGAGAGGATTTAGATTTATCAAAGATCCGTTATTT  34358
                ||||||||||| || ||   |   |||||||| |||   ||| ||||| | |  ||||||
Sbjct  1816769  GAATATAAAGCTCAACAGTCTTGCGAGAGAGGGTTTGCTTTTCTCAAATACCAATTATTT  1816710

Query  34359    TTTACTTCCAGTGTATTCGTGAAAAAGCCGGAGCGAGTTGAAGCAATCGGAATGATAATG  34418
                ||| |   |||| | ||| | ||||  || ||| || | ||  |  | |||||||| |||
Sbjct  1816709  TTTGCAGACAGTATTTTCCTAAAAAGTCCAGAGAGAATAGAGTCCCTGGGAATGATTATG
1816650
```

Query 34419 GG 34420
||
Sbjct 1816649 GG 1816648

Range 52: 3638515 to 3638636

Score:54.5 bits(59), Expect:1e-05,
Identities:85/122(70%), Gaps:0/122(0%), Strand: Plus/Plus

Query 34299 GAATATAAAGCGCAGCAAAGTAATGAGAGAGGATTTAGATTTATCAAAGATCCGTTATTT 34358
||||||||||| || || | |||||||| ||| ||| ||||| | | ||||||
Sbjct 3638515 GAATATAAAGCTCAACAGTCTTGCGAGAGAGGGTTTGCTTTTCTCAAATACCAATTATTT 3638574

Query 34359 TTTACTTCCAGTGTATTCGTGAAAAAGCCGGAGCGAGTTGAAGCAATCGGAATGATAATG 34418
||| | |||| | ||| | |||| || ||| || | || | | |||||||| |||
Sbjct 3638575 TTTGCAGACAGTATTTTCCTAAAAAGTCCAGAGAGAATAGAGTCCCTGGGAATGATTATG
3638634

Query 34419 GG 34420
||
Sbjct 3638635 GG 3638636

Range 53: 1177574 to 1177612

Score:53.6 bits(58), Expect:1e-05,
Identities:35/39(90%), Gaps:0/39(0%), Strand: Plus/Minus

Query 34230 TCTGGGAGATTTATATTAGCCACAAATATCTTAGATACT 34268
|||||||||||||| ||||| || ||||| |||||||||
Sbjct 1177612 TCTGGGAGATTTATTTTAGCAACTAATATTTTAGATACT 1177574

Range 54: 1532357 to 1532477

Score:53.6 bits(58), Expect:1e-05,
Identities:88/124(71%), Gaps:5/124(4%), Strand: Plus/Minus

Query 34299 GAATATAAAGCGCAGCAAAGTAATG--AGAGAGGATTTAGATTTATCAAAGATCCGTTAT 34356
||||||||||| || | ||| || ||||||| ||| ||| ||||||| || |||
Sbjct 1532477 GAATATAAAGCTCA--ACAGTCTTGCCAGAGAGGGTTTGCTTTTCTCAAAGACCCATTA- 1532421

Query 34357 TTTTTACTTCCAGTGTATTCGTGAAAAAGCCGGAGCGAGTTGAAGCAATCGGAATGATAA 34416
||||| | |||| | ||| | |||| || ||| || | || | | |||||||| |
Sbjct 1532420 TTTTTGCAGACAGTATTTTCCTAAAAAGTCCAGAGAGAATAGAGTCCCTGGGAATGATTA 1532361

Query 34417 TGGG 34420
||||
Sbjct 1532360 TGGG 1532357

Range 55: 3578747 to 3578816

Score:53.6 bits(58), Expect:1e-05,
Identities:55/71(77%), Gaps:1/71(1%), Strand: Plus/Minus

```
Query  33881    TTATGCTGGCATCAAGCAAAGATGGCTAGTAATAGAAAGTAAAATCAGAAAGCAAGCAGA  33940
                |||||  || ||  ||||||||||||| || |||| |||  |  ||||||  |||||||
Sbjct  3578816  TTATGGGGGAATAGAGCAAAGATGGCTATTAGTAGACAGTGCAGACAGAAAAAAAGCAGA
3578757

Query  33941    TCTTaaaaaaa  33951
                 ||||||| ||
Sbjct  3578756  -CTTAAAAGAA  3578747
```

Range 56: 4252527 to 4252722

Score:53.6 bits(58), Expect:1e-05,
Identities:141/204(69%), Gaps:15/204(7%), Strand: Plus/Minus

```
Query  33756    ATGAAATGGATAACGAGAGTACCATTAAGCATCAAAGAAGCTCAAAATAAAATCTGCAAG   33815
                ||||||||||||   ||||||| |||| ||| ||| |||| |||||  |  |    ||||
Sbjct  4252722  ATGAAATGGATAAGTCGAGTACCTTTAACCATTAAAAAAGC-CAAAA--ACTTGGTCAAG  4252666

Query  33816    --ATTGAAGA-TAGTGC-CTGGGAAAACCC--TGAAATAAAAGGATATAAAATAGCAGTC  33869
                  ||| |  | ||| |  ||    |||||    |||||| ||||||||||    || ||
Sbjct  4252665  GCATTTACCAGTAGAGAACTC---AAACCAAGTGAAATCAAAGGATATAGC-TATCAAGA  4252610

Query  33870    CAAACGAGTGA-TTATGCTGGCATCAAGCAAAGATGGCTAGTAATAGAAAGTAAAATCAG   33928
                  ||  |||   |||||  || ||  |||||||||||||  | ||||||||  |  |||
Sbjct  4252609  AGAAAAAGTATCTTATGGGGGAATAGAGCAAAGATGGCTATGAGTAGAAAGTGCAGACAG
4252550

Query  33929    AAAGCAAGCAGATCTTaaaaaaaT  33952
                |||  ||||| | |||||| ||||
Sbjct  4252549  AAAAAAAGCA-AACTTAAAGAAAT  4252527
```

Range 57: 4285284 to 4285393

Score:52.7 bits(57), Expect:5e-05,
Identities:81/112(72%), Gaps:3/112(2%), Strand: Plus/Minus

```
Query  33842    TGAAATAAAAGGATATAAAATAGCAGTCCAAACGAGTGA-TTATGCTGGCATCAAGCAAA  33900
                |||||| ||||||||||    || ||  |  ||  |||    |||||  || ||  ||||||
Sbjct  4285393  TGAAATCAAAGGATATAGC-TATCAAGCAGAAAAAGTATCTTATGAGGGAATAGAGCAAA  4285335

Query  33901    GATGGCTAGTAATAGAAAGTAAAATCAGAAAGCAAGCAGATCTTaaaaaaaT  33952
                ||||||||  | ||||||||  |  ||||||  ||||||| |||| | ||||
```

file:///C/...SUBMISSION%20AUGUST%2021%202024/SUPPLEMENTARY%20MATERIAL/BLASTN_Alignments_anatoxina_Nazollae.txt[22/08/2024 13:13:51]

```
Sbjct  4285334  GATGGCTATCAGTAGAAAGTGCAGACAGAAAAAAAGCAGA-CTTACAGAAAT  4285284
```

Range 58: 4677380 to 4677489

Score:52.7 bits(57), Expect:5e-05,
Identities:81/112(72%), Gaps:3/112(2%), Strand: Plus/Minus

```
Query  33842    TGAAATAAAAGGATATAAAATAGCAGTCCAAACGAGTGA-TTATGCTGGCATCAAGCAAA  33900
                |||||| ||||||||||   || ||  |  ||  |||   |||||  || ||  ||||||
Sbjct  4677489  TGAAATCAAAGGATATAGC-TATCAAGCAGAAAAAGTATCTTATGGGGGAATAGAGCAAA  4677431

Query  33901    GATGGCTAGTAATAGAAAGTAAAATCAGAAAGCAAGCAGATCTTaaaaaaaT  33952
                ||||||||  | ||||||||  |  ||||||  ||||||| |||| | ||||
Sbjct  4677430  GATGGCTATGAGTAGAAAGTGCAGACAGAAAAAAAGCAGA-CTTACACAAAT  4677380
```

Range 59: 3671237 to 3671306

Score:51.8 bits(56), Expect:5e-05,
Identities:56/72(78%), Gaps:2/72(2%), Strand: Plus/Minus

```
Query  33881    TTATGCTGGCATCAAGCAAAGATGGCTAGTAATAGAAAGTAAAATCAGAAAGCAAGCAGA  33940
                ||||| || ||  || ||||||||||| || ||||||||  |  ||||||  |||||||
Sbjct  3671306  TTATGGGGGAATAGAGAAAAGATGGCTATTAGTAGAAAGTGCAGACAGAAA-AAAGCAGA
3671248

Query  33941    TCTTaaaaaaaT  33952
                 |||||| ||||
Sbjct  3671247  -CTTAAAGAAAT  3671237
```

Range 60: 905872 to 905942

Score:50.9 bits(55), Expect:2e-04,
Identities:55/72(76%), Gaps:1/72(1%), Strand: Plus/Plus

```
Query  33881   TTATGCTGGCATCAAGCAAAGATGGCTAGTAATAGAAAGTAAAATCAGAAAGCAAGCAGA  33940
               ||||| || ||  |||||||||||||  | |||| |||  |  ||||||  |||||||
Sbjct  905872  TTATGGGGGAATAGAGCAAAGATGGCTATGAGTAGACAGTGGAGACAGAAAAAAAGCAGA  905931

Query  33941   TCTTaaaaaaaT  33952
                |||||| ||||
Sbjct  905932  -CTTAAAGAAAT  905942
```

Range 61: 2098463 to 2098583

Score:50.9 bits(55), Expect:2e-04,

```
Identities:85/122(70%),  Gaps:1/122(0%), Strand: Plus/Minus

Query  34299    GAATATAAAGCGCAGCAAAGTAATGAGAGAGGATTTAGATTTATCAAAGATCCGTTATTT  34358
                ||||||||||| || ||   |   |||||||| |||   ||| ||||||| || ||| ||
Sbjct  2098583  GAATATAAAGCTCAACAGTCTTGCGAGAGAGGGTTTGCTTTTCTCAAAGACCCATTA-TT  2098525

Query  34359    TTTACTTCCAGTGTATTCGTGAAAAAGCCGGAGCGAGTTGAAGCAATCGGAATGATAATG  34418
                ||| |   |||| | ||| | ||||   | ||| || | ||  |  | |||||||| |||
Sbjct  2098524  TTTGCAGACAGTATTTTCCTAAAAAGTTCAGAGAGAATAGATTCCCTGGGAATGATTATG  2098465

Query  34419    GG  34420
                ||
Sbjct  2098464  GG  2098463



Range 62: 2131790 to 2131829

Score:50.9 bits(55), Expect:2e-04,
Identities:35/40(88%),  Gaps:0/40(0%), Strand: Plus/Minus

Query  10894    ATTTGGATAAGCCATTTGCGATCTTTGGTCATAGTATGGG  10933
                || ||||||| || ||||| || ||||||||||||||||
Sbjct  2131829  ATCTGGATAAACCTTTTGCTATTTTTGGTCATAGTATGGG  2131790



Range 63: 3925475 to 3925545

Score:50.9 bits(55), Expect:2e-04,
Identities:55/72(76%),  Gaps:1/72(1%), Strand: Plus/Plus

Query  33881    TTATGCTGGCATCAAGCAAAGATGGCTAGTAATAGAAAGTAAAATCAGAAAGCAAGCAGA  33940
                |||||  || ||  |||||||||||||  | ||||||||  |  ||||||  ||||| |
Sbjct  3925475  TTATGGGGGAATAGAGCAAAGATGGCTATGAGTAGAAAGTGCAGACAGAAAAAAAGCA-A
3925533

Query  33941    TCTTaaaaaaaT  33952
                 |||||| ||||
Sbjct  3925534  ACTTAAAGAAAT  3925545



Range 64: 4584975 to 4585045

Score:50.9 bits(55), Expect:2e-04,
Identities:55/72(76%),  Gaps:1/72(1%), Strand: Plus/Minus

Query  33881    TTATGCTGGCATCAAGCAAAGATGGCTAGTAATAGAAAGTAAAATCAGAAAGCAAGCAGA  33940
                |||||  || ||  |||||||||||||  | ||||||||  |  ||||||  ||||| |
Sbjct  4585045  TTATGGGGGAATAGAGCAAAGATGGCTATGAGTAGAAAGTGCAGACAGAAAAAAAGCA-A
4584987

Query  33941    TCTTaaaaaaaT  33952
```

```
                |||||| ||||
Sbjct  4584986  ACTTAAAGAAAT  4584975
```

Range 65: 5251808 to 5251930

Score:50.9 bits(55), Expect:2e-04,
Identities:88/125(70%), Gaps:5/125(4%), Strand: Plus/Plus

```
Query  34299    GAATATAAAGCGCAGCAAAGTAATG--AGAGAGGATTTAGATTTATCAAAGATCCGTTAT  34356
                ||||||||||| ||  | |||  ||  ||||||| |||   ||| ||||||| || ||||
Sbjct  5251808  GAATATAAAGCTCA--ACAGTCTTGCCAGAGAGGGTTTGCTTTTCTCAAAGACCCATTAT  5251865

Query  34357    TTTTTACTTCCAGTGTATTCGTGAAAAAGCCGGAGCGAGTTGAAGCAATCGGAAT-GATA  34415
                ||||| |   |||| | ||| |  |||  || ||| || | ||  |  | ||||| |||
Sbjct  5251866  TTTTTGCAGACAGTATTTTCCTACAAAGTCCAGAGAGAATAGAGTCCCTGGGAATGGATT
5251925

Query  34416    ATGGG  34420
                |||||
Sbjct  5251926  ATGGG  5251930
```

Range 66: 469454 to 469687

Score:50.0 bits(54), Expect:2e-04,
Identities:156/239(65%), Gaps:6/239(2%), Strand: Plus/Plus

```
Query  33252    TAATAGATGAAAT-GGAATTGGTAGAAGAAGTCAACAAAAAAGTGGGAATAAAAATTAAA  33310
                |||||||||| || ||||||| ||||| || | ||  | | | |  ||||| | | || |
Sbjct  469454   TAATAGATGAGATAGGAATTG-TAGAAAAAATTAATGAGATATTCTGAATAGATAGTAGA  469512

Query  33311    GAAACCCTAAGCCCAGGACAAGTAATGAAAGCAATGATTTTGAACGGACTGGGATTTTTG  33370
                || |   | |   ||||| || || | |||||||| ||| ||    |||| || ||| |
Sbjct  469513   GAGAAAGTGAATACAGGAGAAATAGTCAAAGCAATCATTATG----GACTAGGTTTTCTA  469568

Query  33371    AGCGCCCCAATCTACCTATTCGATGCTTTTTTCGTTGGAAAAGCAACAGAACATCTAATT  33430
                 |    ||| | ||  ||||   |    |||| |  | |||| | | | |||||  | |
Sbjct  469569   TGGAGACCATTATATTTATTTCCTCAACTTTTTGAGGAAAAAACCATATAACATTGATTA  469628

Query  33431    GGAGAAGGAGTGACCCCCGAACAATTAAATGATGACAGAATCGGGAGAGCATTAGATAA  33489
                ||||  ||| |      |||| | ||||||||||| ||||| ||||||| | | |||||
Sbjct  469629   GGAGTGGGAATTGAAGGCGAAGATTTAAATGATGATAGAATAGGGAGAGTAATGGATAA  469687
```

Range 67: 2381227 to 2381265

Score:49.1 bits(53), Expect:6e-04,
Identities:34/39(87%), Gaps:0/39(0%), Strand: Plus/Minus

```
Query  33220    AAATTTAGACCACTTGGGAATAGTAGCAGGGATAATAGA  33258
```

file:///C/...SUBMISSION%20AUGUST%2021%202024/SUPPLEMENTARY%20MATERIAL/BLASTN_Alignments_anatoxina_Nazollae.txt[22/08/2024 13:13:51]

||| |||||||| || || ||||||||||| ||||||||
Sbjct 2381265 AAACTTAGACCATTTAGGCATAGTAGCAGGAATAATAGA 2381227

Range 68: 4055058 to 4055096

Score:49.1 bits(53), Expect:6e-04,
Identities:34/39(87%), Gaps:0/39(0%), Strand: Plus/Plus

Query 33220 AAATTTAGACCACTTGGGAATAGTAGCAGGGATAATAGA 33258
||| |||||||| || || ||||||||||| ||||||||
Sbjct 4055058 AAACTTAGACCATTTAGGCATAGTAGCAGGAATAATAGA 4055096

Range 69: 4585592 to 4585829

Score:49.1 bits(53), Expect:6e-04,
Identities:162/247(66%), Gaps:14/247(5%), Strand: Plus/Minus

Query 33248 GGGATAATAGATGAAAT-GGAATTGGTAGAAGAAGTCAACAAAAAAGTGGGAATAAAAAT 33306
|| |||| |||||| || ||||||| ||||| | |||| | | | | ||||| | |
Sbjct 4585829 GGAATAAGAGATGAGATAGGAATTG-TAGAAATAATCAATAGGATATTCTGAATAGATAG 4585771

Query 33307 TAAAGAAACCCTAAGCCCAGGACAAGTAATGAAAGCAATGATTTTGAACGGACTGGGATT 33366
|| |||| | | ||||| || || | |||||||| ||| || |||| || ||
Sbjct 4585770 TAGCGAAAAAGTGAATACAGGAGAAATAGTCAAAGCAATCATTATG----GACTAGGTTT 4585715

Query 33367 TTTGAGCGCCCCAATCTACCTATTCGATGCTTTTTTCGTT---GGAAAAG-CAACAGAAC 33422
| | | ||| | || |||| |||| || || ||||||| | | ||||
Sbjct 4585714 TCTATGGAGACCATTATATTTATT----GCTTCGATCTTTTGAGGAAAAGTCCATAGAAT 4585659

Query 33423 ATCTAATTGGAGAAGGAGTGACCCCCGAACAATTAAATGATGACAGAATCGGGAGAGCAT 33482
|| | | |||| ||| | |||| | ||||||||||| ||||| |||||||| |
Sbjct 4585658 ATTGATTAGGAGCGGGAATTGAAGGCGAAGATTTAAATGATGATAGAATAGGGAGAGTAA
4585599

Query 33483 TAGATAA 33489
| |||||
Sbjct 4585598 TGGATAA 4585592

Range 70: 3671853 to 3672087

Score:48.2 bits(52), Expect:6e-04,
Identities:154/239(64%), Gaps:4/239(1%), Strand: Plus/Minus

Query 33251 ATAATAGATGAAATGGAATTGGTAGAAGAAGTCAACAAAAAAGTGGGAATAAAAATTAAA 33310
|||||| |||| | | | | |||||||| |||| || | | | ||||| | | || |
Sbjct 3672087 ATAATATATGAGAGAGCAATTCTAGAAGAAATCAATAAGATATTCTGAATAGATAGTAGA 3672028

Query 33311 GAAACCCTAAGCCCAGGACAAGTAATGAAAGCAATGATTTTGAACGGACTGGGATTTTTG 33370

||| | | | |||| || || | |||||||| ||| || |||| || ||| |
Sbjct 3672027 GAGAAAGTGAATATAGGAGAAATAGTCAAAGCAATCATTATG----GACTAGGTTTTCTA 3671972

Query 33371   AGCGCCCCAATCTACCTATTCGATGCTTTTTTCGTTGGAAAAGCAACAGAACATCTAATT 33430
| ||| | || |||| | |||| | | |||||| | ||||||| | |
Sbjct 3671971 TGGAGACCATTATATTTATTTCCTCAACTTTTTGAGGAAAAAGCCATAGAACATTGATTA 3671912

Query 33431   GGAGAAGGAGTGACCCCCGAACAATTAAATGATGACAGAATCGGGAGAGCATTAGATAA 33489
|||| ||| | | ||| | ||||||||||| ||||| || |||| | | |||||
Sbjct 3671911 GGAGCGGGAATTAAAGGTGAAGATTTAAATGATGATAGAATAGGCAGAGTAATGGATAA 3671853

Range 71: 148 to 200

Score:47.3 bits(51), Expect:0.002,
Identities:42/53(79%), Gaps:0/53(0%), Strand: Plus/Plus

Query 33225 TAGACCACTTGGGAATAGTAGCAGGGATAATAGATGAAATGGAATTGGTAGAA 33277
||||||| || || ||||||||||| |||||||| | || | | | ||||||
Sbjct 148   TAGACCATTTAGGCATAGTAGCAGGAATAATAGACGCCATAGGAGTAGTAGAA 200

Range 72: 6108 to 6160

Score:47.3 bits(51), Expect:0.002,
Identities:42/53(79%), Gaps:0/53(0%), Strand: Plus/Minus

Query 33225 TAGACCACTTGGGAATAGTAGCAGGGATAATAGATGAAATGGAATTGGTAGAA 33277
||||||| || || ||||||||||| |||||||| | || | | | ||||||
Sbjct 6160   TAGACCATTTAGGCATAGTAGCAGGAATAATAGACGCCATAGGAGTAGTAGAA 6108

Range 73: 408994 to 409046

Score:47.3 bits(51), Expect:0.002,
Identities:42/53(79%), Gaps:0/53(0%), Strand: Plus/Plus

Query 33225   TAGACCACTTGGGAATAGTAGCAGGGATAATAGATGAAATGGAATTGGTAGAA 33277
||||||| || || ||||||||||| |||||||| | || | | | ||||||
Sbjct 408994 TAGACCATTTAGGCATAGTAGCAGGAATAATAGACGCCATAGGAGTAGTAGAA 409046

Range 74: 664184 to 664236

Score:47.3 bits(51), Expect:0.002,
Identities:42/53(79%), Gaps:0/53(0%), Strand: Plus/Plus

Query 33225   TAGACCACTTGGGAATAGTAGCAGGGATAATAGATGAAATGGAATTGGTAGAA 33277
||||||| || || ||||||||||| |||||||| | || | | | ||||||

Sbjct 664184 TAGACCATTTAGGCATAGTAGCAGGAATAATAGACGCCATAGGAGTAGTAGAA 664236

Range 75: 902798 to 902850

Score:47.3 bits(51), Expect:0.002,
Identities:42/53(79%), Gaps:0/53(0%), Strand: Plus/Plus

```
Query  33225   TAGACCACTTGGGAATAGTAGCAGGGATAATAGATGAAATGGAATTGGTAGAA  33277
               ||||||| || || ||||||||||| |||||||| |  || | | | ||||||
Sbjct  902798  TAGACCATTTAGGCATAGTAGCAGGAATAATAGACGCCATAGGAGTAGTAGAA  902850
```

Range 76: 1195025 to 1195077

Score:47.3 bits(51), Expect:0.002,
Identities:42/53(79%), Gaps:0/53(0%), Strand: Plus/Plus

```
Query  33225    TAGACCACTTGGGAATAGTAGCAGGGATAATAGATGAAATGGAATTGGTAGAA  33277
                ||||||| || || ||||||||||| |||||||| |  || | | | ||||||
Sbjct  1195025  TAGACCATTTAGGCATAGTAGCAGGAATAATAGACGCCATAGGAGTAGTAGAA  1195077
```

Range 77: 1277927 to 1277979

Score:47.3 bits(51), Expect:0.002,
Identities:42/53(79%), Gaps:0/53(0%), Strand: Plus/Minus

```
Query  33225    TAGACCACTTGGGAATAGTAGCAGGGATAATAGATGAAATGGAATTGGTAGAA  33277
                ||||||| || || ||||||||||| |||||||| |  || | | | ||||||
Sbjct  1277979  TAGACCATTTAGGCATAGTAGCAGGAATAATAGACGCCATAGGAGTAGTAGAA  1277927
```

Range 78: 1317141 to 1317193

Score:47.3 bits(51), Expect:0.002,
Identities:42/53(79%), Gaps:0/53(0%), Strand: Plus/Plus

```
Query  33225    TAGACCACTTGGGAATAGTAGCAGGGATAATAGATGAAATGGAATTGGTAGAA  33277
                ||||||| || || ||||||||||| |||||||| |  || | | | ||||||
Sbjct  1317141  TAGACCATTTAGGCATAGTAGCAGGAATAATAGACGCCATAGGAGTAGTAGAA  1317193
```

Range 79: 1534601 to 1534653

Score:47.3 bits(51), Expect:0.002,
Identities:42/53(79%), Gaps:0/53(0%), Strand: Plus/Minus

```
Query  33225    TAGACCACTTGGGAATAGTAGCAGGGATAATAGATGAAATGGAATTGGTAGAA  33277
                ||||||| || || ||||||||||| |||||||| |  || | | | ||||||
Sbjct  1534653  TAGACCATTTAGGCATAGTAGCAGGAATAATAGACGCCATAGGAGTAGTAGAA  1534601
```

Range 80: 2838809 to 2838861

Score:47.3 bits(51), Expect:0.002,
Identities:42/53(79%), Gaps:0/53(0%), Strand: Plus/Plus

```
Query  33225    TAGACCACTTGGGAATAGTAGCAGGGATAATAGATGAAATGGAATTGGTAGAA  33277
                ||||||| || || ||||||||||| |||||||| |  || | | | ||||||
Sbjct  2838809  TAGACCATTTAGGCATAGTAGCAGGAATAATAGACGCCATAGGAGTAGTAGAA  2838861
```

Range 81: 3005150 to 3005202

Score:47.3 bits(51), Expect:0.002,
Identities:42/53(79%), Gaps:0/53(0%), Strand: Plus/Minus

```
Query  33225    TAGACCACTTGGGAATAGTAGCAGGGATAATAGATGAAATGGAATTGGTAGAA  33277
                ||||||| || || ||||||||||| |||||||| |  || | | | ||||||
Sbjct  3005202  TAGACCATTTAGGCATAGTAGCAGGAATAATAGACGCCATAGGAGTAGTAGAA  3005150
```

Range 82: 3262945 to 3262997

Score:47.3 bits(51), Expect:0.002,
Identities:42/53(79%), Gaps:0/53(0%), Strand: Plus/Minus

```
Query  33225    TAGACCACTTGGGAATAGTAGCAGGGATAATAGATGAAATGGAATTGGTAGAA  33277
                ||||||| || || ||||||||||| |||||||| |  || | | | ||||||
Sbjct  3262997  TAGACCATTTAGGCATAGTAGCAGGAATAATAGACGCCATAGGAGTAGTAGAA  3262945
```

Range 83: 3424177 to 3424229

Score:47.3 bits(51), Expect:0.002,
Identities:42/53(79%), Gaps:0/53(0%), Strand: Plus/Minus

```
Query  33225    TAGACCACTTGGGAATAGTAGCAGGGATAATAGATGAAATGGAATTGGTAGAA  33277
                ||||||| || || ||||||||||| |||||||| |  || | | | ||||||
Sbjct  3424229  TAGACCATTTAGGCATAGTAGCAGGAATAATAGACGCCATAGGAGTAGTAGAA  3424177
```

Range 84: 3466060 to 3466112

Score:47.3 bits(51), Expect:0.002,
Identities:42/53(79%), Gaps:0/53(0%), Strand: Plus/Minus

```
Query  33225    TAGACCACTTGGGAATAGTAGCAGGGATAATAGATGAAATGGAATTGGTAGAA  33277
                ||||||| || || ||||||||||| |||||||| |  || | | | ||||||
Sbjct  3466112  TAGACCATTTAGGCATAGTAGCAGGAATAATAGACGCCATAGGAGTAGTAGAA  3466060
```

Range 85: 4566957 to 4567009

Score:47.3 bits(51), Expect:0.002,
Identities:42/53(79%), Gaps:0/53(0%), Strand: Plus/Minus

```
Query  33225    TAGACCACTTGGGAATAGTAGCAGGGATAATAGATGAAATGGAATTGGTAGAA  33277
                ||||||| || || ||||||||||| |||||||| |  || | | | ||||||
Sbjct  4567009  TAGACCATTTAGGCATAGTAGCAGGAATAATAGACGCCATAGGAGTAGTAGAA  4566957
```

Range 86: 4567345 to 4567397

Score:47.3 bits(51), Expect:0.002,
Identities:42/53(79%), Gaps:0/53(0%), Strand: Plus/Plus

```
Query  33225    TAGACCACTTGGGAATAGTAGCAGGGATAATAGATGAAATGGAATTGGTAGAA  33277
                ||||||| || || ||||||||||| |||||||| |  || | | | ||||||
Sbjct  4567345  TAGACCATTTAGGCATAGTAGCAGGAATAATAGACGCCATAGGAGTAGTAGAA  4567397
```

Range 87: 4596070 to 4596122

Score:47.3 bits(51), Expect:0.002,
Identities:42/53(79%), Gaps:0/53(0%), Strand: Plus/Plus

```
Query  33225    TAGACCACTTGGGAATAGTAGCAGGGATAATAGATGAAATGGAATTGGTAGAA  33277
                ||||||| || || ||||||||||| |||||||| |  || | | | ||||||
Sbjct  4596070  TAGACCATTTAGGCATAGTAGCAGGAATAATAGACGCCATAGGAGTAGTAGAA  4596122
```

Range 88: 4599705 to 4599757

Score:47.3 bits(51), Expect:0.002,
Identities:42/53(79%), Gaps:0/53(0%), Strand: Plus/Minus

```
Query  33225    TAGACCACTTGGGAATAGTAGCAGGGATAATAGATGAAATGGAATTGGTAGAA  33277
                ||||||| || || ||||||||||| |||||||| |  || | | | ||||||
Sbjct  4599757  TAGACCATTTAGGCATAGTAGCAGGAATAATAGACGCCATAGGAGTAGTAGAA  4599705
```

Range 89: 4755512 to 4755564

Score:47.3 bits(51), Expect:0.002,
Identities:42/53(79%), Gaps:0/53(0%), Strand: Plus/Minus

```
Query  33225    TAGACCACTTGGGAATAGTAGCAGGGATAATAGATGAAATGGAATTGGTAGAA  33277
                ||||||| || || ||||||||||| |||||||| |  || | | | ||||||
Sbjct  4755564  TAGACCATTTAGGCATAGTAGCAGGAATAATAGACGCCATAGGAGTAGTAGAA  4755512
```

Range 90: 4851258 to 4851310

Score:47.3 bits(51), Expect:0.002,
Identities:42/53(79%), Gaps:0/53(0%), Strand: Plus/Plus

```
Query  33225    TAGACCACTTGGGAATAGTAGCAGGGATAATAGATGAAATGGAATTGGTAGAA  33277
                ||||||| || || ||||||||||| |||||||| |  || | | | ||||||
Sbjct  4851258  TAGACCATTTAGGCATAGTAGCAGGAATAATAGACGCCATAGGAGTAGTAGAA  4851310
```

Range 91: 5254817 to 5254869

Score:47.3 bits(51), Expect:0.002,
Identities:42/53(79%), Gaps:0/53(0%), Strand: Plus/Minus

```
Query  33225    TAGACCACTTGGGAATAGTAGCAGGGATAATAGATGAAATGGAATTGGTAGAA  33277
                ||||||| || || ||||||||||| |||||||| |  || | | | ||||||
Sbjct  5254869  TAGACCATTTAGGCATAGTAGCAGGAATAATAGACGCCATAGGAGTAGTAGAA  5254817
```

Range 92: 5352125 to 5352177

Score:47.3 bits(51), Expect:0.002,
Identities:42/53(79%), Gaps:0/53(0%), Strand: Plus/Minus

```
Query  33225    TAGACCACTTGGGAATAGTAGCAGGGATAATAGATGAAATGGAATTGGTAGAA  33277
                ||||||| || || ||||||||||| |||||||| |  || | | | ||||||
Sbjct  5352177  TAGACCATTTAGGCATAGTAGCAGGAATAATAGACGCCATAGGAGTAGTAGAA  5352125
```

Range 93: 2325405 to 2325471

Score:45.5 bits(49), Expect:0.007,
Identities:50/67(75%), Gaps:0/67(0%), Strand: Plus/Plus

```
Query  1965     TTAATGTGGTTTCAAAAATCCTCTGTTTGCAGTTTTGACCTCTGCTTTTTGCAGTTCGCT  2024
                ||| ||||||||||||| | |  ||||||| |||    |     |   | ||||||||||| |
```

Sbjct 2325405 TTATTGTGGTTTCAAAATTGCCTTGTTTGCGGTTCGAAGGGTTTATATTTGCAGTTCGTT 2325464

Query 2025 ACACTTA 2031
|||||||
Sbjct 2325465 ACACTTA 2325471

Range 94: 1799889 to 1799922

Score:44.6 bits(48), Expect:0.007,
Identities:30/34(88%), Gaps:0/34(0%), Strand: Plus/Minus

Query 33225 TAGACCACTTGGGAATAGTAGCAGGGATAATAGA 33258
||||||| || || ||||||||||| ||||||||
Sbjct 1799922 TAGACCATTTAGGTATAGTAGCAGGAATAATAGA 1799889

Range 95: 1809690 to 1809723

Score:44.6 bits(48), Expect:0.007,
Identities:30/34(88%), Gaps:0/34(0%), Strand: Plus/Plus

Query 33225 TAGACCACTTGGGAATAGTAGCAGGGATAATAGA 33258
||||||| || || ||||||||||| ||||||||
Sbjct 1809690 TAGACCATTTAGGTATAGTAGCAGGAATAATAGA 1809723

Range 96: 1817975 to 1818008

Score:44.6 bits(48), Expect:0.007,
Identities:30/34(88%), Gaps:0/34(0%), Strand: Plus/Minus

Query 33225 TAGACCACTTGGGAATAGTAGCAGGGATAATAGA 33258
||||||| || || ||||||||||| ||||||||
Sbjct 1818008 TAGACCATTTAGGTATAGTAGCAGGAATAATAGA 1817975

Range 97: 2391700 to 2391733

Score:44.6 bits(48), Expect:0.007,
Identities:30/34(88%), Gaps:0/34(0%), Strand: Plus/Minus

Query 33225 TAGACCACTTGGGAATAGTAGCAGGGATAATAGA 33258
||||||| || || ||||||||||| ||||||||
Sbjct 2391733 TAGACCATTTAGGCATAGTAGCAGGAATAATAGA 2391700

Range 98: 3637267 to 3637325

Score:44.6 bits(48), Expect:0.007,
Identities:45/59(76%), Gaps:0/59(0%), Strand: Plus/Plus

```
Query  33225    TAGACCACTTGGGAATAGTAGCAGGGATAATAGATGAAATGGAATTGGTAGAAGAAGTC  33283
                ||||||| || |  ||||||||||| |||||||| |  || | | | ||||||  ||||
Sbjct  3637267  TAGACCATTTAGTCATAGTAGCAGGAATAATAGACGCCATAGGAGTCGTAGAAATAGTC  3637325
```

Range 99: 4253286 to 4253394

Score:44.6 bits(48), Expect:0.007,
Identities:78/110(71%), Gaps:3/110(2%), Strand: Plus/Minus

```
Query  33248    GGGATAATAGATGAAAT-GGAATTGGTAGAAGAAGTCAACAAAAAAGTGGGAATA-AAAA  33305
                || ||||||||||| || |||||| ||||||  | |||| || | | |  ||||| | ||
Sbjct  4253394  GGAATAATAGATGAGATAGGAATT-GTAGAAAGAATCAATAAGACATTCTGAATAGATAA  4253336

Query  33306    TTAAAGAAACCCTAAGCCCAGGACAAGTAATGAAAGCAATGATTTTGAAC  33355
                ||| ||| |   | |    |||| || || | |||| ||| ||| |||||
Sbjct  4253335  TTAGAGAGAAAGTGAATATAGGAGAAATAGTTAAAGGAATCATTATGAAC  4253286
```

Range 100: 4374735 to 4374768

Score:44.6 bits(48), Expect:0.007,
Identities:30/34(88%), Gaps:0/34(0%), Strand: Plus/Plus

```
Query  33225    TAGACCACTTGGGAATAGTAGCAGGGATAATAGA  33258
                ||||||| || || ||||||||||| ||||||||
Sbjct  4374735  TAGACCATTTAGGTATAGTAGCAGGAATAATAGA  4374768
```

Range 101: 4799474 to 4799507

Score:44.6 bits(48), Expect:0.007,
Identities:30/34(88%), Gaps:0/34(0%), Strand: Plus/Plus

```
Query  33225    TAGACCACTTGGGAATAGTAGCAGGGATAATAGA  33258
                ||||||| || || ||||||||||| ||||||||
Sbjct  4799474  TAGACCATTTAGGCATAGTAGCAGGAATAATAGA  4799507
```

Range 102: 2199446 to 2199499

Score:43.7 bits(47), Expect:0.025,
Identities:44/55(80%), Gaps:2/55(3%), Strand: Plus/Minus

Query 33230   CACTTGGGAATAGTAGCAGGGATAATAGATGAAAT-GGAATTGGTAGAAGAAGTC 33283
|| || | || | ||||||| ||||||||||| || |||||| ||||| |||||
Sbjct 2199499 CAATTAGAAAAAATAGCAGGAATAATAGATGAGATAGGAATT-GTAGACAAAGTC 2199446

Range 103: 3003823 to 3003964

Score:43.7 bits(47), Expect:0.025,
Identities:97/144(67%), Gaps:4/144(2%), Strand: Plus/Minus

Query 34299   GAATATAAAGCGCAGCAAAGTAATG--AGAGAGGATTTAGATTTATCAAAGATCCGTTAT 34356
|||||||||| | | ||| || ||||||| ||| ||| ||||||| || ||||
Sbjct 3003964 GAATATAAAGCTTA--ACAGTCTTGCCAGAGAGGGTTTGCTTTTCTCAAAGACCCATTAT 3003907

Query 34357   TTTTTACTTCCAGTGTATTCGTGAAAAAGCCGGAGCGAGTTGAAGCAATCGGAATGATAA 34416
| ||| | |||| | | | | ||| || ||| || | || | | ||||||||| |
Sbjct 3003906 TCTTTGCAGACAGTATTTACCTAGAAAGTCCAGAGAGAATAGAGTCCCTGGGAATGATTA
3003847

Query 34417   TGGGACTGTGTTTGTTAGTATATA 34440
||| | ||| || | || ||||
Sbjct 3003846 TGGTTTTATGTCTGCTGGTTTATA 3003823

Range 104: 74266 to 74346

Score:42.8 bits(46), Expect:0.025,
Identities:60/82(73%), Gaps:2/82(2%), Strand: Plus/Minus

Query 33409 AAAAGCAACAGAACATCTAATTGGAGAAGGAG-TGACCCCCGAACAATTAAATGATGACA 33467
|||||| | ||||||| || | |||| ||| ||| | ||| | ||||||||||| |
Sbjct 74346 AAAAGCTATAGAACATTTATTAGGAGCGGGAACTGAAGGC-GAAGATTTAAATGATGATA 74288

Query 33468 GAATCGGGAGAGCATTAGATAA 33489
|||| || | || | | |||||
Sbjct 74287 GAATAGGCACAGTAATGGATAA 74266

Range 105: 2317904 to 2317956

Score:42.8 bits(46), Expect:0.025,
Identities:41/53(77%), Gaps:0/53(0%), Strand: Plus/Plus

Query 33225   TAGACCACTTGGGAATAGTAGCAGGGATAATAGATGAAATGGAATTGGTAGAA 33277
||||||| | || ||||||||||| |||||||| | || | | | ||||||
Sbjct 2317904 TAGACCATTGAGGCATAGTAGCAGGAATAATAGACGCCATAGGAGTAGTAGAA 2317956

Range 106: 2350382 to 2350434

Score:42.8 bits(46), Expect:0.025,
Identities:41/53(77%), Gaps:0/53(0%), Strand: Plus/Plus

```
Query  33225    TAGACCACTTGGGAATAGTAGCAGGGATAATAGATGAAATGGAATTGGTAGAA  33277
                ||||||| |  || ||||||||||| |||||||| |  || | | | ||||||
Sbjct  2350382  TAGACCATTGAGGCATAGTAGCAGGAATAATAGACGCCATAGGAGTAGTAGAA  2350434
```

Range 107: 2462903 to 2462955

Score:42.8 bits(46), Expect:0.025,
Identities:41/53(77%), Gaps:0/53(0%), Strand: Plus/Plus

```
Query  33225    TAGACCACTTGGGAATAGTAGCAGGGATAATAGATGAAATGGAATTGGTAGAA  33277
                ||||||| |  || ||||||||||| |||||||| |  || | | | ||||||
Sbjct  2462903  TAGACCATTGAGGCATAGTAGCAGGAATAATAGACGCCATAGGAGTAGTAGAA  2462955
```

Range 108: 2500173 to 2500225

Score:42.8 bits(46), Expect:0.025,
Identities:41/53(77%), Gaps:0/53(0%), Strand: Plus/Minus

```
Query  33225    TAGACCACTTGGGAATAGTAGCAGGGATAATAGATGAAATGGAATTGGTAGAA  33277
                ||||||| |  || ||||||||||| |||||||| |  || | | | ||||||
Sbjct  2500225  TAGACCATTGAGGCATAGTAGCAGGAATAATAGACGCCATAGGAGTAGTAGAA  2500173
```

Range 109: 3977603 to 3977640

Score:42.8 bits(46), Expect:0.025,
Identities:32/38(84%), Gaps:0/38(0%), Strand: Plus/Minus

```
Query  17180    TTGTTCACCGGACAAGGTTCGCAATACGTTGATATGGG  17217
                |||||| | ||||||||||| |||||| | || |||||
Sbjct  3977640  TTGTTCTCTGGACAAGGTTCTCAATACCTAGAAATGGG  3977603
```

Range 110: 4819178 to 4819230

Score:42.8 bits(46), Expect:0.025,
Identities:41/53(77%), Gaps:0/53(0%), Strand: Plus/Plus

```
Query  33225    TAGACCACTTGGGAATAGTAGCAGGGATAATAGATGAAATGGAATTGGTAGAA  33277
                ||||||| |  || ||||||||||| |||||||| |  || | | | ||||||
Sbjct  4819178  TAGACCATTGAGGCATAGTAGCAGGAATAATAGACGCCATAGGAGTAGTAGAA  4819230
```
